# Luminescent Platinum(II) Complexes with Terdentate N∧C∧C Ligands

**DOI:** 10.1021/acs.inorgchem.3c02399

**Published:** 2023-12-05

**Authors:** Dionisio Poveda, Ángela Vivancos, Delia Bautista, Pablo González-Herrero

**Affiliations:** † Departamento de Química Inorgánica, Facultad de Química, Universidad de Murcia, Campus de Espinardo, 19, 30100 Murcia, Spain; ‡ Área Científica y Técnica de Investigación, Universidad de Murcia, Campus de Espinardo, 21, 30100 Murcia, Spain

## Abstract

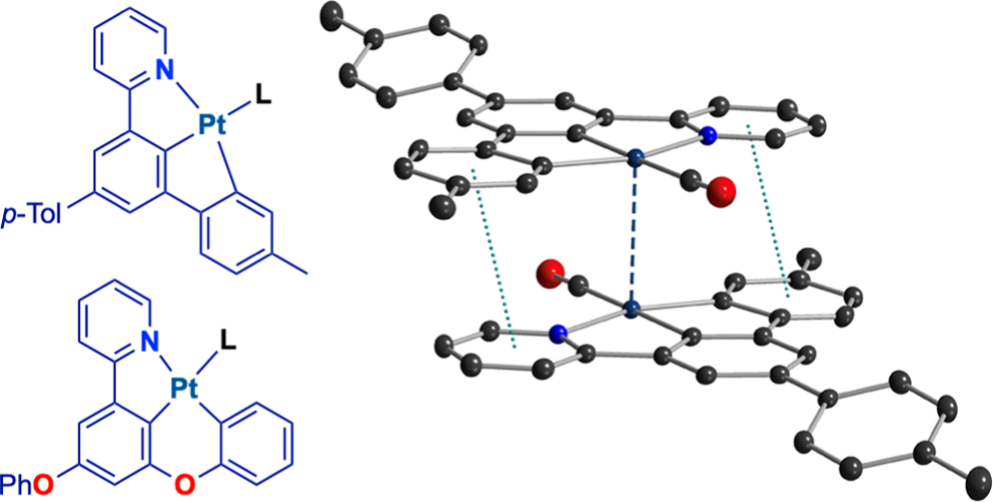

The synthesis, structure, and luminescence of Pt(II) complexes of the type [Pt(N∧C∧C)(L)] are reported, where N∧C∧C is a terdentate ligand resulting from the cycloplatination of 2-(3,5-diphenoxyphenyl)pyridine or 2-(4,4″-dimethyl-[1,1′:3′,1″-terphenyl]-5′-yl)pyridine, and L represents a monodentate ancillary ligand, which can be γ-picoline, 4-pyridinecarboxaldehyde, PPh_3_, *n-*butyl or 2,6-dimethylphenyl isocyanide, CO, or the N-heterocyclic carbenes 1-butyl-3-methylimidazol-2-ylidene or 4-butyl-3-methyl-1-phenyl-1*H*-1,2,3-triazol-5-ylidene. Derivatives bearing CO, isocyanides, or carbenes showed the highest stabilities in solution, whereas the pyridine and PPh_3_ derivatives establish ligand-exchange equilibria in acetonitrile. Different supramolecular structures are observed in the solid state, which largely depend on the nature of the ancillary ligand. Isocyanides and CO favor π interactions between the aromatic rings, metallophilic Pt···Pt contacts, or a combination of both. In contrast, pyridine ligands may lead to bimolecular assemblies driven by C–H···O, C–H···Pt, or C–H/π hydrogen bonds. Luminescence was examined in fluid solution, poly(methyl methacrylate) matrices, and the solid state at 298 K, and in 2-methyltetrahydrofuran glasses at 77 K. The majority of derivatives show highly efficient emissions from ^3^ILCT/MLCT or ^3^ILCT/MLCT/LLCT excited states of monomeric species. The formation of excimers and different types of emissive aggregates are demonstrated, which lead to red-shifted emissions of different origins and characteristics depending on the involved noncovalent interactions.

## Abstract 2

Pt(II) complexes with terdentate N∧C∧C ligands are demonstrated as strong phosphorescent emitters with valuable possibilities for luminescence modulation, including the formation of excimers and different types of emissive molecular assemblies.

## Introduction

Pt(II) complexes with chelating heteroaromatic ligands have been intensively studied for their excited-state properties, which make them suitable for diverse photochemical, analytical, and optoelectronic applications.^1,2^ Thus, they often exhibit highly efficient phosphorescent emissions with tunable characteristics, which can be conveniently adjusted for application as phosphors for organic light-emitting devices (OLEDs),^3−8^ probes for bioimaging,^9−11^ or chemosensors.^12−15^ They also exhibit an interesting reactivity in the excited state^16^ and can function as photoredox catalysts for organic synthesis.^17−20^ Cyclometalated 2-arylpyridines (C∧N) have long been the most frequently employed ligands for the design of luminescent Pt(II) complexes because the metalated aryl is a strong σ-donor that induces a large ligand-field splitting, resulting in higher energies of dissociative metal-centered (MC or d–d) excited states and enhanced stabilities and emission efficiencies with respect to complexes with 2,2′-bipyridines (N∧N) and related ligands.^4^ Therefore, many efficient Pt(II) emitters are of the type [Pt(C∧N)(L∧X)], where L∧X is a monoanionic chelating ligand, often a β-diketonate.^21,22^ Increasing the number of C-donor moieties is desirable to achieve higher emission efficiencies, but bis-cyclometalated complexes of the type *cis*-[Pt(C∧N)_2_] are generally not emissive,^23^ which has been attributed to molecular flexibility and the geometrical distortions caused by the steric hindrance between the C∧N ligands.^24^ The development of more efficient Pt(II) emitters has often been addressed by using terdentate N∧N∧C,^25−27^ N∧C∧N,^28−35^ or C∧N∧C^36−38^ heteroaromatic ligands, which impart rigidity and reduce nonradiative deactivation. However, the N∧C∧N and N∧N∧C ligand classes provide only one C-donor moiety, and C∧N∧C ligands often lead to weakly emissive complexes because the trans arrangement of metalated carbon atoms causes significant distortions in the excited state.^39^ Different types of Pt(II) emitters bearing cis-disposed metalated aryl rings have been developed by introducing biaryl ligands, which may produce intense luminescence.^40−44^ Additionally, heteroaromatic tetradentate C∧N∧N∧C,^24,45−49^ N∧C∧C∧N,^14,15,50−54^ or C∧C∧N∧N^55^ ligands have been designed to provide increased rigidity and, at the same time, a cis arrangement of metalated aryls, leading to very high emission efficiencies; however, their synthesis usually involves laborious procedures and their cycloplatination can be challenging.

An aspect of great importance to the development of Pt(II) emitters is the possibility to modulate their luminescence through the formation of aggregates or molecular assemblies,^2,35,64−69,56−63^ which can occur via π interactions between the aromatic systems of the ligands and/or metallophilic Pt···Pt contacts, that usually fall in the range 3.0–3.5 Å.^59,60^ These interactions, either separately or in combination, can cause significant modifications of frontier orbitals or favor the formation of supramolecular entities, such as aggregates or excimers, which usually produce emissions that are red-shifted with respect to those from the monomeric complexes. Several environmental factors can affect the formation of these entities, such as the solvent or solid matrix, the concentration, and the presence of other molecules that may interact with the complex. Aggregation phenomena can be used as a basis for the development of functional supramolecular architectures^60,70^ and chemical sensors capable of responding to various stimuli, such as the presence of volatile organic compounds,^71−73^ metal ions,^74,75^ or even mechanical processes.^76−78^

Terdentate heteroaromatic N∧C∧C ligands are relatively uncommon, yet they have been demonstrated as excellent platforms for the development of robust Au(III) emitters, such as those derived from the auration of 2-(3,5-diarylphenyl)pyridines reported by the groups of Nevado^79^ and Yam.^80^ The introduction of the simplest heteroaromatic N∧C∧C ligand, namely, 2-([1,1′-biphenyl]-3-yl)pyridine, into the coordination sphere of Au(III)^81^ and Pd(II)^82^ has been shown by Breher and co-workers to afford highly luminescent complexes; this research group has also recently reported a luminescent Pt(II) complex bearing this ligand.^83^ Several Ir(III) N∧C∧C complexes have also been reported.^84^ N∧C∧C ligands meet significant characteristics that make them promising for the design of versatile and efficient Pt(II) emitters since they provide a strong σ donation from cis-disposed aryl moieties, present largely planar structures to enable aggregation phenomena, and, like other terdentate ligands, offer additional possibilities for excited-state modulation through the introduction of different ancillary ligands at the fourth coordination position. Therefore, it is surprising that N∧C∧C ligands have not yet been systematically exploited to develop luminescent Pt(II) complexes.

In a previous communication, we have reported the cycloplatination of 2-(3,5-diphenoxyphenyl)pyridine (dPhOppyH_2_) and 2-(4,4″-dimethyl-[1,1′:3′,1″-terphenyl]-5′-yl)pyridine (dmtppyH_2_) using a photochemical protocol, and demonstrated the synthesis of complexes of the types [PtCl(N∧C∧C)]^−^ and [Pt(N∧C∧C)(L)] (L = NCPh, γ-picoline).^85^ In this article, we present the synthesis, structure, and luminescence of an extended series of derivatives [Pt(N∧C∧C)(L)], which can reach high emission efficiencies. Luminescence modulation is demonstrated by varying the electronic and steric properties of the monodentate ancillary ligand (L), which can affect excited-state energies or determine the formation of different types of molecular assemblies.

## Results and Discussion

### Synthesis

**Scheme 1:**
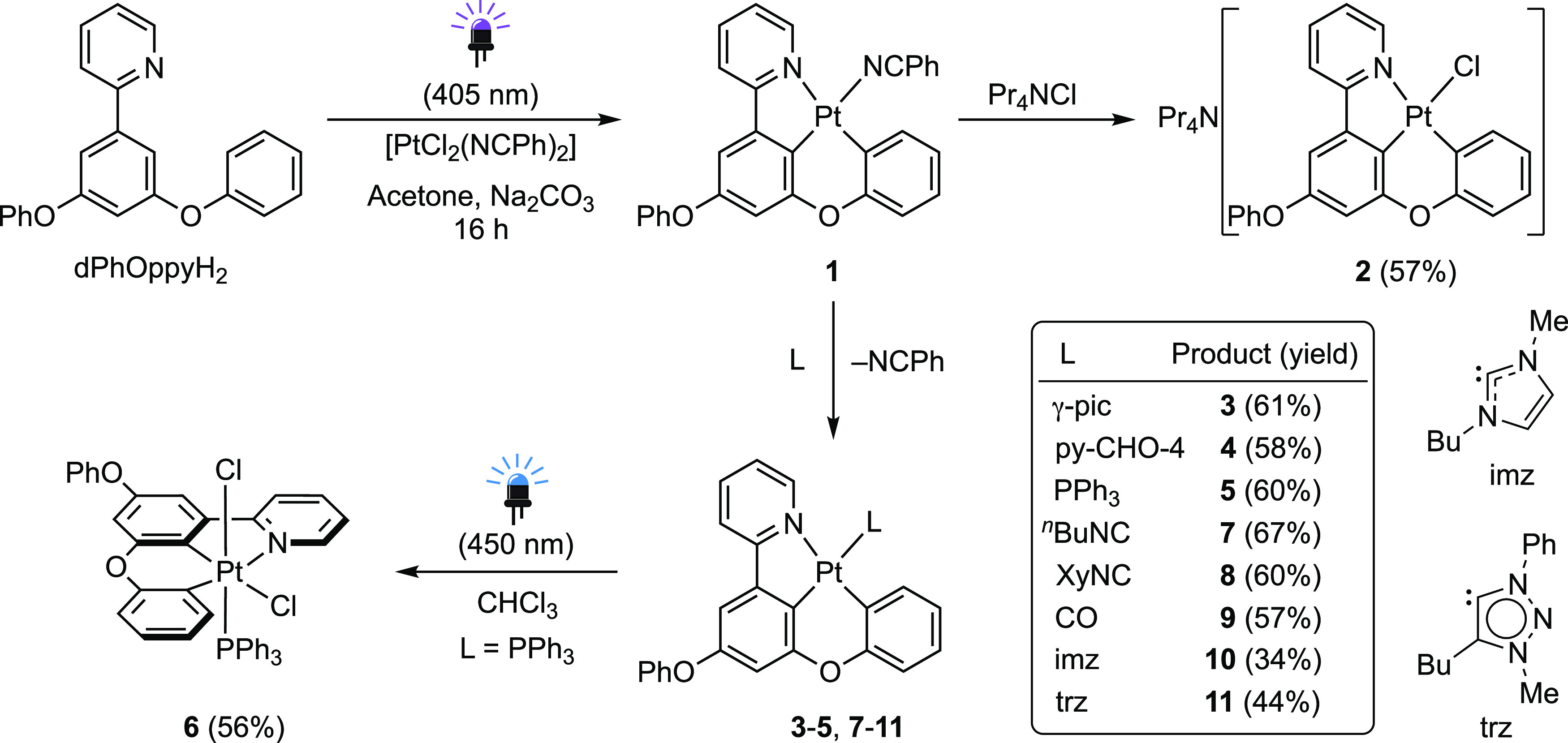
Synthesis of Pt(II) and Pt(IV) Derivatives Bearing the dPhOppy Ligand

In our previous report,^85^ we showed that cycloplatination of dPhOppyH_2_ or dmtppyH_2_ can be easily achieved under irradiation with violet light (λ_max_ = 405 nm) using (Bu_4_N)_2_[Pt_2_Cl_6_] or [PtCl_2_(NCPh)_2_] as metal precursors in the presence of a base. In the case of dPhOppyH_2_, the best option was to use [PtCl_2_(NCPh)_2_], which led to neutral complex [Pt(dPhOppy)(NCPh)] (**1**, Scheme 1). To synthesize a series of derivatives bearing dPhOppy, complex **1** was photogenerated and treated in situ with different ligands to avoid the losses associated with isolation. Substitution of the benzonitrile ligand by chloride succeeded by treating **1** with Pr_4_NCl in CH_2_Cl_2_ at room temperature to give the anionic complex Pr_4_N[PtCl(dPhOppy)] (**2**), which was found to be only moderately stable in solution, reasonably because the strong kinetic trans effect of the metalated central aryl ring labilizes the Pt–Cl bond, leading to dissociation. Treatment of **1** with γ-picoline (γ-pic) or 4-pyridinecarboxaldehyde (py-CHO-4) led to the corresponding neutral complexes [Pt(dPhOppy)(L)] (**3** or **4**, respectively), which were stable in acetone and CH_2_Cl_2_ solutions for several days. However, in MeCN solution, they engage in ligand-exchange equilibria with the solvent. This was demonstrated by the ^1^H NMR spectrum of **3** in CD_3_CN, which showed the presence of free γ-picoline and a new complex bearing the dPhOppy ligand that was identified as [Pt(dPhOppy)(NCCD_3_)] (Figure S15). Similarly to the case for **3** and **4**, the complex [Pt(dPhOppy)(PPh_3_)] (**5**) was easily obtained by treating **1** with PPh_3_ in acetone. Although it showed higher stability toward dissociation, small amounts of [Pt(dPhOppy)(NCCD_3_)] and free PPh_3_ were detected in its ^1^H or ^31^P NMR spectra, respectively, in CD_3_CN. Complex **5** was found to be sensitive to ambient light in CDCl_3_ solution, undergoing oxidation to [PtCl_2_(dPhOppy)(PPh_3_)] (**6**). The deliberate synthesis of this complex could be achieved by irradiating a solution of **5** in CHCl_3_ with blue LEDs (λ_max_ = 450 nm), and its identity was confirmed by a single-crystal X-ray diffraction analysis (see below). Considering that chloroform does not absorb in the visible region (cutoff wavelength: 250 nm^86^), a solvent-initiated radical pathway can be ruled out. We hypothesize that the formation of **6** proceeds through a radical mechanism triggered by the reduction of chloroform by photoexcited **5**; related photoreductions of halogenated solvents^87,88^ or iodobenzene^89^ by cyclometalated Pt(II) complexes have been previously reported.

Treatment of **1** with *n*-butyl isocyanide (BuNC), 2,6-dimethylphenyl isocyanide (XyNC), or CO afforded the corresponding neutral [Pt(dPhOppy)(L)] complexes (**7**–**9**) at room temperature in acetone. These complexes are much more stable toward dissociation compared to **3**–**5**, indicating that stronger π acceptor ligands are better suited for the available coordination position, certainly because they partially relieve the excess electronic density at the metal caused by the strong σ and π donations from the metalated aryls of the dPhOppy ligand. The N-heterocyclic carbenes 1-butyl-3-methylimidazol-2-ylidene (imz) and 4-butyl-3-methyl-1-phenyl-1*H*-1,2,3-triazol-5-ylidene (trz) could be successfully introduced via transmetalation from the in situ generated silver carbenes at 40 °C in 1,2-dichloroethane and gave very stable complexes (**10** and **11**, respectively).

**Scheme 2:**
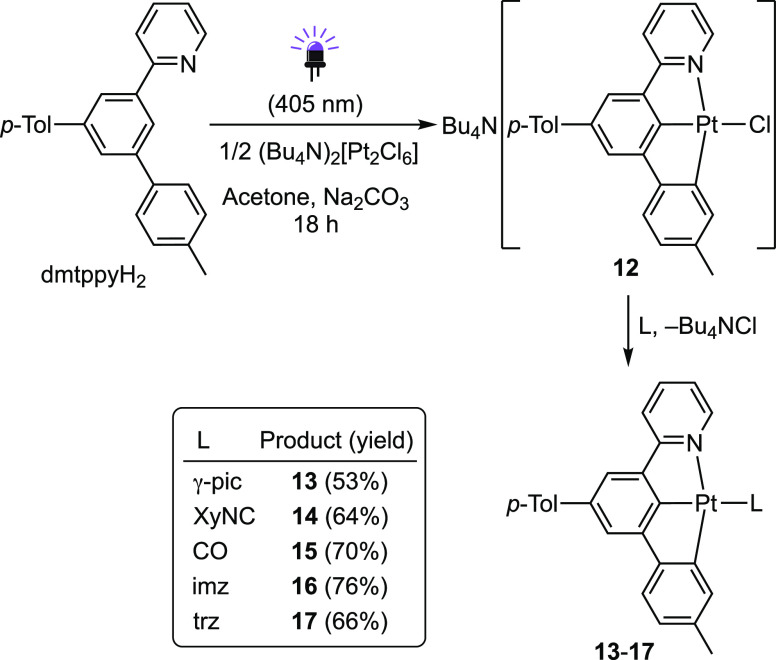
Synthesis of Pt(II) Complexes Bearing the dmtppy Ligand

To synthesize Pt(II) derivatives bearing the dmtppy ligand, the precursor Bu_4_N[PtCl(dmtppy)] (**12**) was employed, which can be conveniently obtained upon photochemical cycloplatination of dmtppyH_2_ using (Bu_4_N)_2_[Pt_2_Cl_6_] (Scheme 2).^85^ As observed for **2**, the chlorido ligand in **12** is rather labile and was easily substituted by γ-picoline, XyNC or CO in dichloromethane at room temperature to give the corresponding [Pt(dmtppy)(L)] complexes (**13**–**15**). The carbenes imz and trz were also introduced (complexes **16** and **17**, respectively) through the same methodology employed for the dPhOppy complexes.

### Crystal Structures

**Figure 1:**
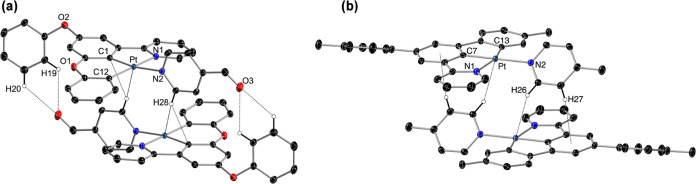
Assembled centrosymmetric pairs in the crystal structures of **4** (a) and **13** (b) (thermal ellipsoids at 50% probability). Hydrogen atoms are omitted except those involved in hydrogen bonding.

**Figure 2:**
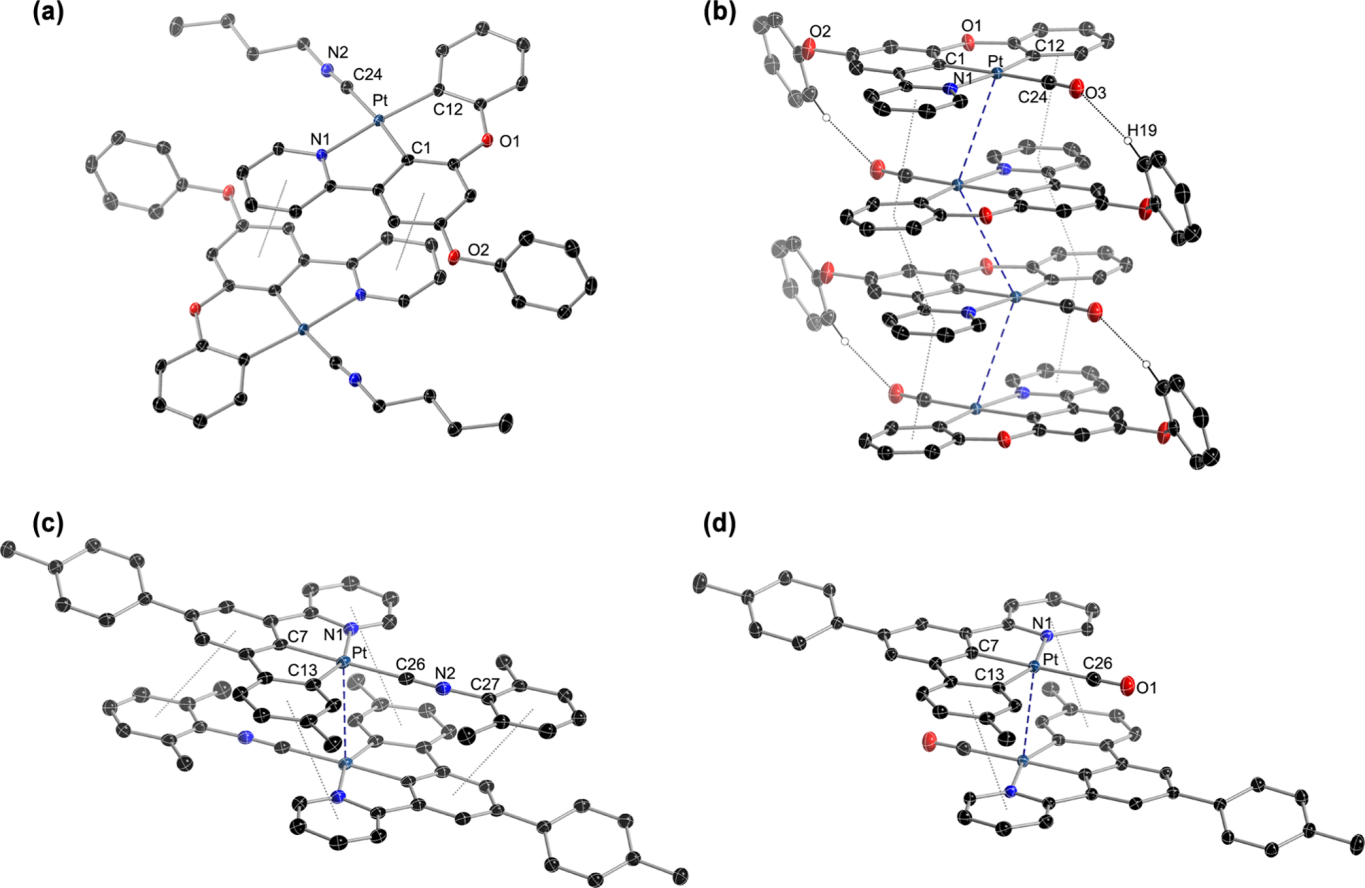
Structures of **7** (a), **9** (b), **14** (c), and **15** (d) in the crystal, showing π stacking, Pt···Pt or C–H···O interactions (thermal ellipsoids at 50% probability). Hydrogen atoms are omitted, except those involved in C–H···O interactions. π stacking interactions are indicated as dotted lines between ring centroids.

**Figure 3:**
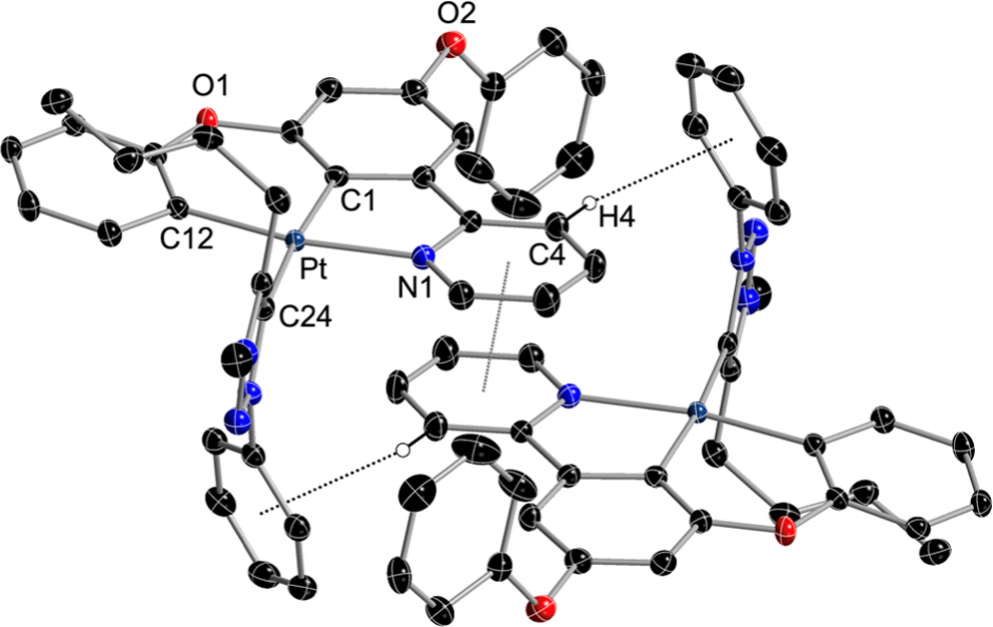
Structure of **11** in the crystal (stacked dimer; thermal ellipsoids at 50% probability). Hydrogen atoms are omitted except for those involved in CH/π interactions.

**Figure 4:**
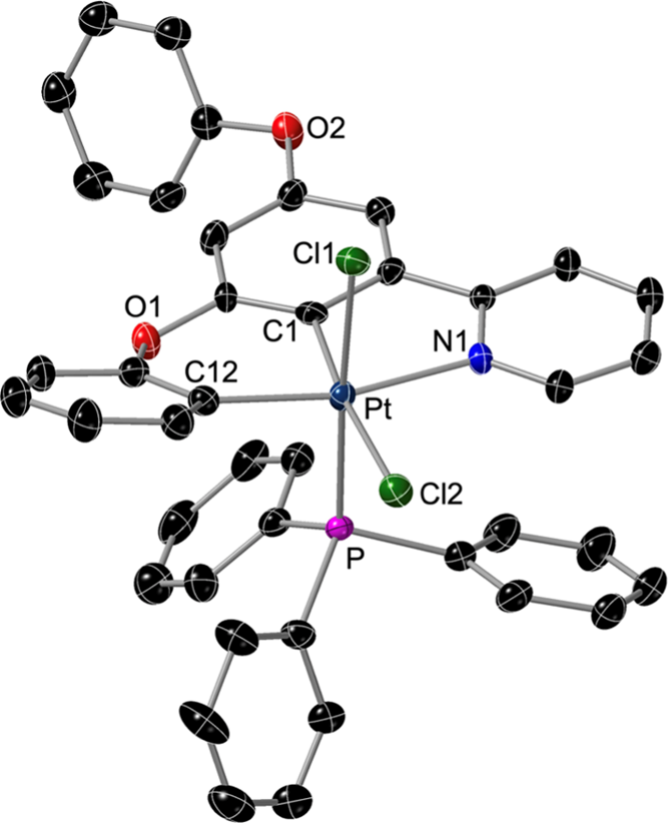
Structure of **6** in the crystal (thermal ellipsoids at 50% probability). Hydrogen atoms are omitted.

The crystal structures of complexes **4**, **6**, **7**, **9**, **11**, **14**, and **15** have been solved by X-ray diffraction (Figures 1–4), while those of **3** and **13** have been previously reported^85^ and are analyzed here for comparison. Selected bond distances and angles are compiled in the Supporting Information. Both dPhOppy and dmtppy can lead to highly planar geometries in their Pt(II) complexes, excluding the pendant, nonmetalated phenoxy or tolyl group, and show small root-mean-square deviations (RMSD) from the mean plane formed by the metal and the three bonded aromatic rings for complexes **7**, **9**, **13**–**15** (range 0.021–0.079 Å); the corresponding RMSDs in complexes **3** (0.185 Å), **4** (0.125 Å), and **11** (0.100 Å) are higher, possibly because of their particular packing or intermolecular interactions (see below). As expected, the angles around the Pt atom for the dmtppy derivatives show considerable deviations from the ideal square-planar values because of the constraints imposed by the 5-membered chelate rings, with N1–Pt–C7 and C7–Pt–C13 angles around 80° and N1–Pt–C13 angles around 160°. The dPhOppy ligand leads to less strained coordination environments due to the 6-membered chelate ring with C1–Pt–C12 angles around 90° and N1–Pt–C12 angles around 170°. Also, the dmtppy ligand forces a shorter Pt–C bond distance for the central aromatic ring (Pt–C7, range 1.934–1.977 Å) compared to dPhOppy (Pt–C1, range 1.959–1.993 Å), which is only slightly affected by the ancillary ligand in trans.

The crystal packing in the structures of the Pt(II) derivatives was analyzed to determine the existence of π stacking, metallophilic Pt···Pt contacts, or other noncovalent interactions that could affect their luminescence. The crystal structure of **3** did not show any π stacking or Pt···Pt contacts, but only intermolecular C–H···O hydrogen bonds^90^ that do not lead to an easily recognizable assembly pattern (Figure S16). In contrast, the molecules of **4** assemble in centrosymmetric pairs (Figure 1), in which the {Pt(dPhOppy)} subunits are arranged in an antiparallel orientation. The 4-formylpyridine ring plane is almost perpendicular to the coordination mean plane (88.9°), and its N atom sticks out from this plane by ca. 0.725 Å, apparently to allow a closer approximation between the molecules, thereby causing a significant deviation from planarity of the coordination environment. A close look at this assembly showed that π interactions between the aromatic rings of the dPhOppy ligand are not possible because they are too far apart. The intermetallic distance of 4.506 Å is too long for a metallophilic interaction. However, there are contacts between one of the hydrogens ortho to the N atom of the 4-formylpyridine ligand (H28) and the Pt and C1 atoms of the adjacent molecule (2.932 or 2.681 Å, respectively), that can be described as bifurcated C–H···Pt/C hydrogen bonds.^91−93^ Reasonably, atom H28 has significant partial positive charge, and there must be an electrostatic attraction to the region along the Pt–C1 bond. There are also C–H···O hydrogen bonds between the formyl group (O3) and two H atoms of the pendant phenoxy group (H19, H20) of the other molecule (2.623 or 2.610 Å, respectively) that may significantly contribute to determine the observed assembly.

Similarly to **4**, the molecules of **13** assemble in centrosymmetric pairs (Figure 1) and do not show π stacking involving the aromatic rings of the dmtppy ligand or Pt···Pt interactions, with the shortest intermetallic distance being 4.662 Å. However, the γ-picoline ring is rotated by only 48.5° with respect to the main coordination plane, allowing for a better approximation between the molecules that make up the pair without causing too much deviation from planarity (RMSD of 0.060 Å from the mean plane of the metal and the bonded aromatic rings). Significant intermolecular C–H···Pt hydrogen bonds are observed involving the H atom ortho to the N atom of the γ-picoline ligand (distance Pt···H26: 2.724 Å), whereas the meta H atom (H27) establishes a C–H/π interaction^94,95^ with the central aromatic ring of the dmtppy ligand of the other molecule (distance H27–centroid: 2.661 Å, angle C27–H27–centroid: 133.3°). It is likely that these interactions determine the observed arrangement.

The CO and isocyanide ligands led to diverse stacking patterns that can involve π interactions and Pt···Pt contacts (Figure 2). The parameters that characterize the observed π interactions between individual aromatic rings are given in the Supporting Information (Table S10). The molecules of **7** stack in pairs through π interactions involving the pyridyl and the central ring of the dPhOppy ligand (distance between mean planes formed by the metal and the bonded aromatic rings: 3.321 Å), but there are no Pt···Pt contacts since the closest distance between metal atoms is 6.628 Å. The molecules of **9** form infinite stacks along the *a* axis through π interactions involving the pyridyl and metalated phenoxy rings (Figure 2); within these stacks, dimeric assemblies can be identified that feature Pt···Pt contacts of 3.591 Å and intermolecular C–H···O hydrogen bonds involving the ortho H atom of the pendant phenoxy group (H19) and the CO ligand (distance O3···H19, 2.634 Å), whereas interdimer metal–metal distances are longer (3.805 Å); the distances between mean coordination planes are 3.414 (dimer) and 3.383 Å (interdimer). Complexes **14** and **15** stack in pairs through Pt···Pt interactions (3.290 or 3.222 Å, respectively) and π interactions involving the pyridyl and the metalated tolyl ring (distance between mean coordination planes: 3.379 or 3.344 Å, respectively). In the structure of **15**, an additional π interaction is established between the xylyl ring and the central aromatic ring of the dmtppy ligand. In all cases, adjacent stacked molecules are related by inversion symmetry.

Only one of the two possible rotamers due to restricted rotation about the Pt–NHC bond is observed in the crystal structure of **11** (Figure 3). The triazolylidene ring is rotated by 88.4° with respect to the coordination mean plane (distance between mean ring planes: 3.388 Å). Centrosymmetric pairs are formed through π-stacking interactions between the pyridyl rings. There is also a CH/π interaction between the H4 atom of the pyridyl ring and the Ph ring of the trz ligand of the other molecule (distance H4–centroid: 2.686 Å; angle C4–H4–centroid: 150.1°).

The crystal structure of **6** (Figure 4) revealed mutually cis chlorido ligands and the PPh_3_ ligand at a coordination position orthogonal to the Pt(N∧C∧C) mean plane. The coordination environment around the Pt atom deviates from the ideal octahedral geometry because of the 5-membered chelate ring [C1–Pt–N1, 80.54(16)°] and the steric bulk of the PPh_3_ ligand, which pushes the metalated phenoxy and coordinated pyridyl away from the Pt–P bond, leading to a significant RMSD from the mean plane formed by the metal and the three bonded aromatic rings of the dPhOppy ligand (0.241 Å).

### Absorption Spectra

**Figure 5:**
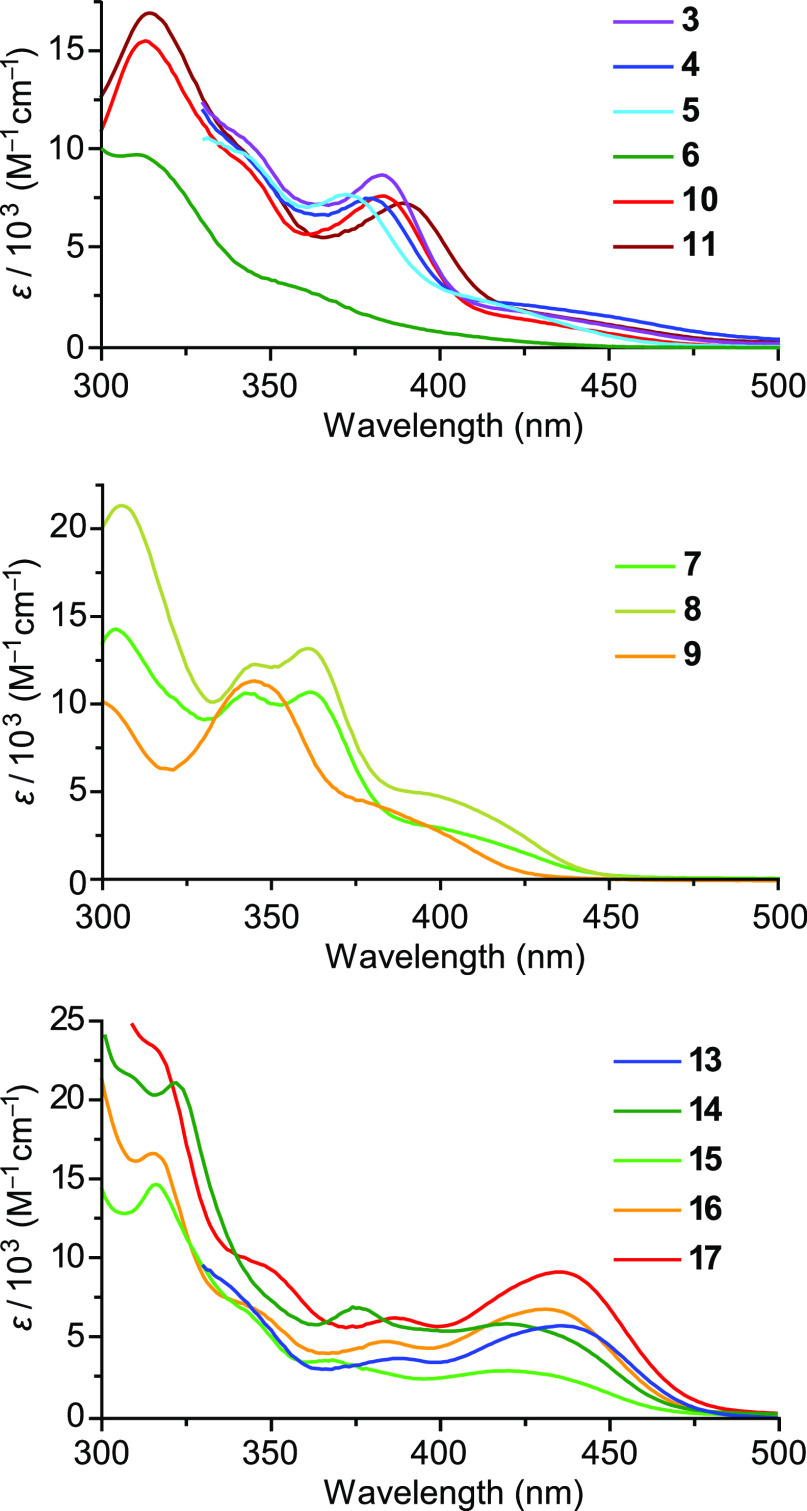
Electronic absorption spectra of the studied complexes in acetone (**3**, **4**, **5**, **13**) or MeCN (rest of complexes) solution (ca. 5 × 10^–5^ M) at 298 K.

The electronic absorption spectra were recorded in acetone solution for complexes **3**, **4**, **5**, and **13** to avoid dissociation and in MeCN for the rest of complexes. Complex **2** was not studied because of its poor stability in solution. The absorption data are collected in Table 1 and the spectra are shown in Figure 5.

**Table 1:** Electronic Absorption Data of the Studied Complexes in Acetone (**3**, **4**, **5**, **13**) or MeCN (Rest of Complexes) Solution (ca. 5 × 10^–5^ M) at 298 K

complex	λ_max_ (nm) (ε/10^2^ (M^–1^ cm^–1^))
**3**	337 (110), 382 (87), 416 (19)
**4**	334 (108), 380 (75), 416 (21)
**5**	340 (98), 372 (77), 403 (27)
**6**	310 (97), 346(36), 386(12)
**7**	304 (143), 343 (106), 362 (107), 393 (31)
**8**	306 (213), 344 (122), 361 (132), 390 (50)
**9**	298 (103), 344 (113), 376 (45)
**10**	313 (155), 335 (101), 383 (76), 416 (15)
**11**	314 (169), 337 (107), 389 (72), 425 (17)
**13**	330 (95), 387 (36), 436 (57)
**14**	306 (218), 322 (210), 375 (68), 419 (58)
**15**	316 (146), 338 (73), 367 (35), 421 (28)
**16**	315 (166), 336 (75), 383 (47), 430 (67)
**17**	313 (236), 342 (100), 386 (62), 435 (91)

Four main absorption bands are distinguishable for the Pt(II) derivatives with the dPhOppy ligand, whose maxima fall around 310, 340, 360–390, and 390–430 nm. The highest-energy band is not observed in the spectra of **3**, **4**, and **5** because they were registered in acetone. The lowest-energy band is very broad and, in most cases, extends as a tail into the visible region, which is responsible for the yellow color of the solutions. The two lowest-energy bands show some dependence on the ancillary ligand, shifting to higher energies for the stronger π acceptors (PPh_3_, isocyanides, and CO). This is consistent with a significant metal-to-ligand charge-transfer (MLCT) character of the responsible electronic transitions, which decreases as the π acceptor character of the ancillary ligand increases, associated with a decrease in the energy of the metal d orbitals. Based on the computational results (see below), these transitions have mixed intraligand charge-transfer (ILCT) and MLCT characters involving the dPhOppy ligand. Compared with **5**, complex **6** shows notably blue-shifted absorptions, which can be ascribed to the loss of MLCT character, as typically observed for cyclometalated Pt(IV) complexes.^89,96^

The series of complexes with dmtppy also give rise to four main absorption bands, with maxima in regions similar to those of the previous series: 310, 340, 360–390, and 420–440 nm. The two lowest-energy bands are blue-shifted for complexes with XyNC and CO, so they must possess significant MLCT character. The lowest-energy band is more intense compared to the dPhOppy derivatives.

To corroborate the charge-transfer character of the lowest-energy absorptions, the absorption spectra of two of the complexes (**11** and **17**) were recorded in solvents of different polarity (Figure S24). For the dPhOppy derivative **11**, the two lowest-energy bands show solvatochromism, although this is most evident for the most intense band. In the case of dmtppy derivative **17**, significant variations are only observed for the lowest-energy band. Although the correlation with solvent polarity was not consistent, the observed behavior demonstrates that the stabilization of the excited and ground states due to solvation undergo significant variations, which is typical of charge-transfer transitions involving a change in the polarity of the molecule.

**Figure 6:**
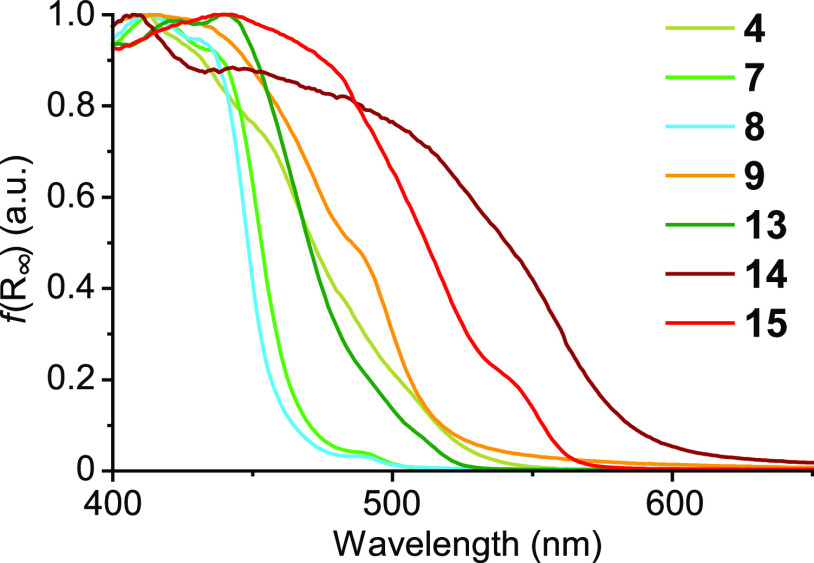
Diffuse reflectance spectra of representative complexes converted by using the Kubelka–Munk function, *f*(*R*_∞_).

Although all of the studied complexes give yellow solutions in acetone or MeCN, some of them are orange (**4**, **15**) or red (**14**) in the solid state, indicating absorption by aggregates. To characterize these absorptions, diffuse reflectance spectra were registered for representative complexes. The reflectance data were converted by using the Kubelka–Munk function and normalized to facilitate comparisons (Figure 6). The contribution from aggregates to absorption in the solid state is important for complexes **4**, **9**, **14**, and **15**, for which the lowest-energy absorption is significantly red-shifted with respect to the solution phase.

### Luminescence at Ambient Temperature

The emission properties were studied for the stable Pt(II) derivatives to determine the influence of the terdentate and ancillary ligands and the effects of aggregation and excimer formation. The Pt(IV) complex **6** was excluded from this study because it underwent partial decomposition upon excitation at the lowest-energy absorption band, probably because of the population of a dissociative ligand-to-metal charge-transfer (LMCT) excited state.^97^ Although complex **5** is photoreactive in CHCl_3_, it was found to be photostable in acetone and therefore could be included in the luminescence study. Excitation and emission spectra were registered in acetone (complexes **3**, **4**, **5**, and **13**, to avoid dissociation) or MeCN (rest of complexes) solutions, poly(methyl methacrylate) (PMMA) matrices (2 wt %), and the solid state (neat films) at 298 K. For a subset of the complexes, measurements in fluid solution were carried out at different concentrations using acetone (**4**, **5**) or MeCN and 2-methyltetrahydrofuran (MeTHF) (**7**–**9**, **14**, **15**) to examine the formation of aggregates or excimers. The measurements in solution and PMMA were carried out in the absence of oxygen. The emission data are listed in Table 2. The complete set of excitation and emission spectra in all media and a chromaticity diagram (CIE coordinates) for the emissions in PMMA matrices are given in the Supporting Information.

**Table 2:** Emission Data of **3**–**5**, **7**–**11**, and **13**–**17**

complex	medium (*T* (K))	λ_em_ (nm)^a^	Φ^b^	τ (μs)^c^
**3**	acetone (298)	548, 570	0.59	6.3
	PMMA (298)	546	0.82	9.4
	solid (298)	557, 579	0.31	1.2 (30%), 3.4 (70%)
	MeTHF (77)	512, 545, 582		5.9
**4**	acetone (298)	581	0.26	1.8 (13%), 6.3 (87%)
	PMMA (298)	561	0.06	8.6 (41%), 19 (59%)
	solid (298)	664	<0.01	
	MeTHF (77)	512, 544, 583		6.0
**5**	acetone (298)	589	0.10	0.9
	PMMA (298)	540	0.13	0.4 (29%), 1.8 (71%)
	solid (298)	567	0.01	0.5 (28%), 1.8 (72%)
	MeTHF (77)	502, 534		10
**7**	MeCN (298)	523, 538	0.60	9.6
	MeTHF (298)	517, 546	0.61	7.4
	PMMA (298)	507, 532	0.81	9.6
	solid (298)	505, 554, 586	0.43	1.1 (15%), 4.9 (85%)
	MeTHF (77)	490, 520, 556		9.0
**8**	MeCN (298)	523, 536	0.79	11
	MeTHF (298)	516, 542	0.60	8.1
	PMMA (298)	507, 528	0.84	10
	solid (298)	638	0.47	3.0 (41%), 7.0 (59%)
	MeTHF (77)	487, 523, 557		11
**9**	MeCN (298)	545	0.75	7.1
	MeTHF(298)	501, 529	0.34	16
	PMMA (298)	492, 526, 560	0.75	10 (13%), 31 (87%)
	solid (298)	500, 612	0.48	0.7 (31%), 1.7 (69%)
	MeTHF (77)	479, 514, 551		40
**10**	MeCN (298)	555	0.64	5.9
	PMMA (298)	545	0.82	8.5
	solid (298)	559	0.30	1.3 (26%), 3.0 (74%)
	MeTHF (77)	509, 545, 583		5.5
**11**	MeCN (298)	550	0.66	6.7
	PMMA (298)	547	0.81	7.8
	solid (298)	563	0.46	2.9 (75%), 10 (25%)
	MeTHF (77)	512, 550, 586		5.3
**13**	acetone (298)	527, 552	0.59	7.8
	PMMA (298)	523, 552	0.71	11
	solid (298)	526, 686	0.08	0.9 (27%), 3.4 (73%)
	MeTHF (77)	514, 551, 589		10
**14**	MeCN (298)	532, 556	0.56	14
	MeTHF(298)	536, 572, 616	0.42	8.7
	PMMA (298)	532, 570, 684	0.48	13 (53%), 8.8 (47%)
	solid (298)	680	0.06	0.6 (35%), 1.2 (65%)
	MeTHF (77)	524, 564, 609		18
**15**	MeCN (298)	525, 547	0.66	12
	MeTHF(298)	543, 582, 629	0.24	14
	PMMA (298)	539, 622, 730	0.52	2.7 (30%), 12 (70%)
	solid (298)	618	0.35	0.6 (24%), 1.2 (76%)
	MeTHF (77)	532, 579, 607		25^d^ 8.5 (26%), 23 (74%)^e^
**16**	MeCN (298)	522, 549	0.57	3.1
	PMMA (298)	521, 548	0.71	14
	solid (298)	561	0.05	0.5 (55%), 2.5 (45%)
	MeTHF (77)	513, 550, 589		11
**17**	MeCN (298)	524, 549	0.50	7.3
	PMMA (298)	522, 545	0.67	13
	solid (298)	561	0.06	0.9 (33%), 3.8 (67%)
	MeTHF (77)	509, 546, 585		9.6

a Main peak maxima.

b Quantum yield; for complexes that form excimers or aggregates in solution, it corresponds to the most diluted solution.

c Lifetime measured at the highest-energy emission peak, except where noted; in the cases of complexes that form excimers in fluid solution (see text), lifetimes correspond to the monomeric emission measured at a concentration for which no excimeric emission is observed; for biexponential decays, relative amplitudes (%) are given in parentheses.

d At the monomeric emission wavelength.

e At the aggregate emission wavelength.

**Figure 7:**
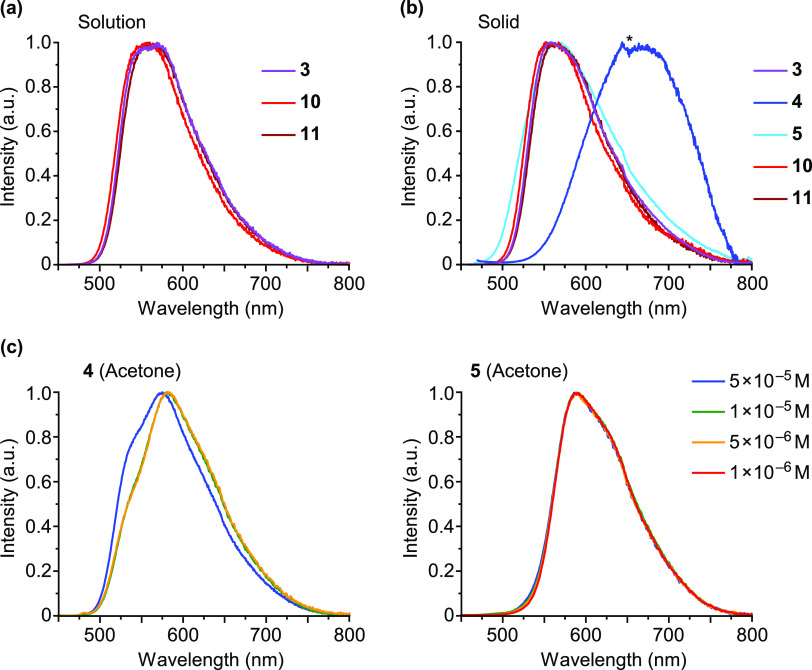
(a) Emission spectra of complexes **3**, **10**, and **11** in solution (5 × 10^–5^ M) at 298 K; the solvent is acetone for **3** and MeCN for **10** and **11**. (b) Emission spectra of **3**, **4**, **5**, **10**, and **11** in the solid state at 298 K; the discontinuity marked with an asterisk is due to imperfect correction of detector sensitivity. (c) Emission spectra of **4** and **5** in acetone at different concentrations at 298 K.

The emission spectra of the dPhOppy derivatives bearing γ-picoline (**3**) or an NHC ligand (**10**, **11**) in fluid solution are virtually identical (Figure 7a), showing a relatively broad band in the yellow region (548–555 nm) with little vibronic structure. This suggests a high degree of MLCT character in the emissive state and little involvement of the ancillary ligand orbitals. Therefore, the emissive excited state must involve orbitals of the dPhOppy ligand and the metal. This is consistent with the computational results, which predict a mixed ILCT/MLCT character for the emissive excited state (see below). The corresponding excitation spectra coincide with the absorption profiles.

The emissions of derivatives **4** and **5** in acetone solution are significantly red-shifted with respect to those of **3**, **10**, or **11**, with a maximum at 581 or 589 nm, respectively. In the case of **4**, the shape of this emission underwent some changes upon varying the concentration in the range from 5 × 10^–5^ to 5 × 10^–6^ M (Figure 7c); the corresponding excitation spectra show differences with respect to the absorption spectrum in acetone (Figure S42). These data are indicative of the presence of emissive molecular aggregates in the acetone solution. The shoulder at 543 nm suggests that emission arising from individual molecules also contributes to the observed spectrum. The emission of **5** in acetone solution did not change in the concentration range from 5 × 10^–5^ to 1 × 10^–6^ M (Figure 7c). In this case, the excitation spectrum is significantly red-shifted with respect to the absorption spectrum (Figure S43), indicating that only molecular aggregates contribute to the observed emission.

The excitation and emission spectra of **3**, **4**, **10**, and **11** in the PMMA matrix are very similar to those observed in acetone or MeCN solution (Figure S37). The emission of **5** in this medium is similar to those of **3**, **10**, and **11** and the corresponding excitation spectrum coincides with the absorption spectrum in acetone. In the solid state, the emissions of **3**, **5**, **10**, and **11** remain almost unchanged with respect to those in PMMA, whereas **4** gives rise to a much more red-shifted emission centered at 664 nm (Figure 7b); the respective excitation spectra can be clearly related to the absorption spectra in solution, excepting **4**, for which a red-shifted band is observed, that resembles the absorption in the solid state obtained from diffuse reflectance data (Figure S47). Therefore, the luminescence of **3**, **5**, **10**, and **11** arises from individual molecules in PMMA and the solid state, while **4** emits exclusively from aggregates in the solid state and partially in PMMA.

**Figure 8:**
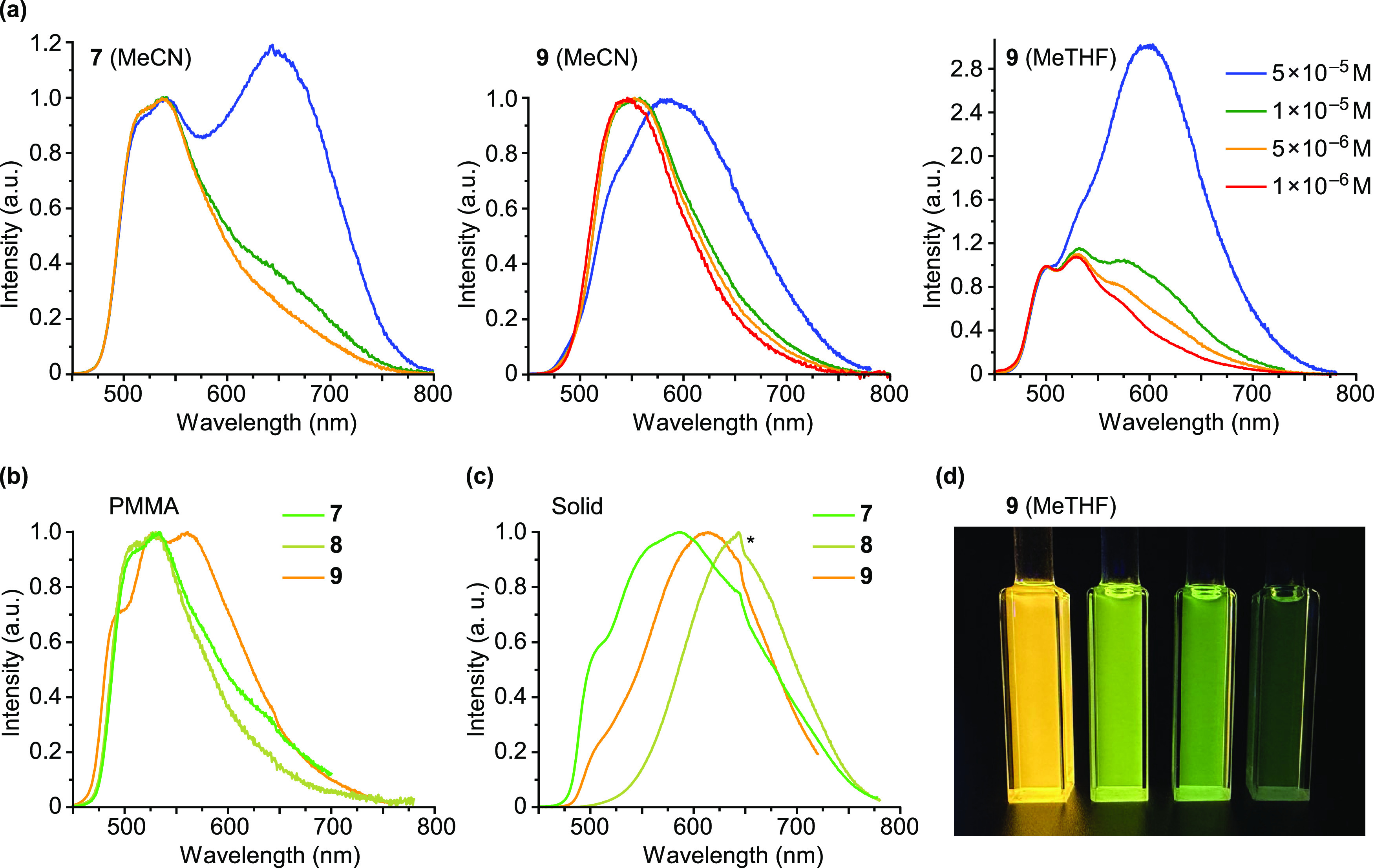
(a) Emission spectra of **7** in MeCN and **9** in MeCN and MeTHF solutions at different concentrations at 298 K. (b) Emission spectra of **7**, **8**, and **9** in PMMA matrices (2 wt %) at 298 K. (c) Emission spectra of **7**, **8**, and **9** in the solid state at 298 K; the discontinuity marked with an asterisk is due to imperfect correction of detector sensitivity. (d) Photograph of MeTHF solutions of **9** at 5 × 10^–5^, 1 × 10^–5^, 5 × 10^–6^, and 1 × 10^–6^ M concentrations (from left to right) under UV irradiation at 298 K.

The BuNC (**7**) and XyNC (**8**) derivatives give rise to two distinct emission bands in fluid MeCN or MeTHF solution at a 5 × 10^–5^ M concentration (Figures 8a, S25, and S27). The high-energy band shows some vibronic structure and is observed at a higher energy compared to **3**, **10**, and **11**, and therefore can be attributed to a similar excited state but with a lower MLCT contribution. Both the decrease in the MLCT contribution and the increase in emission energy are explained by the strong π acceptor character of the isocyanides, which causes a decrease in the energy of the metal orbitals. The very broad, low-energy band falls in the red region (640–650 nm), and its relative intensity with respect to the high-energy band decreases as the concentration is lowered, being practically unobservable at a 5 × 10^–6^ M concentration. Photographs showing the emission color changes upon dilution are included in the Supporting Information (Figures S26 and S28). The excitation spectra monitored at both the high- and low-energy bands are identical and closely reproduce the absorption spectra. These characteristics support an excimeric origin for the low-energy band.

The emission spectrum of the CO derivative **9** in MeCN at a 5 × 10^–5^ M concentration shows a very broad band, with a maximum at 582 nm (Figure 8a). At lower concentrations, the emission shifts to higher energies (545 nm) and becomes narrower. The excitation spectra monitored at the emission maximum are red-shifted with respect to the absorption spectrum (Figure S44), suggesting that the observed emission arises mainly from molecular aggregates. Two shoulders are observed at approximately 485 and 528 nm in the spectrum of the most concentrated solution, which could correspond to vibronic peaks of the emission from individual molecules, indicating a higher emission energy with respect to the monomeric emission of the isocyanide derivatives, which is consistent with the stronger π acceptor character of the CO ligand. This complex exhibits a different behavior in fluid MeTHF solution, where it produces emissions from individual molecules and excimers (Figure 8a), whose excitation spectra coincide with the absorption spectrum. The broad excimeric emission centered at 596 nm is predominant at a 5 × 10^–5^ M concentration, whereas a vibronically structured emission band from the monomer can be isolated at a 1 × 10^–6^ M concentration, with the highest-energy peak at 501 nm, resulting in a marked change in the emission color upon dilution (Figure 8d).

The low energy emissions of complexes **7**, **8**, and **9** are much less important in the PMMA matrix (Figure 8b) as compared to the 5 × 10^–5^ M solutions, becoming barely distinguishable for the isocyanide complexes; in the case of the CO derivative, the vibronic peaks of the monomeric emission are clearly observable in this medium (492 and 526 nm). In the solid state, low energy emissions are predominant for these three derivatives, although in the cases of **7** and **9**, high-energy shoulders are observed, indicating some proportion of monomeric emission. The observed emission of **8** in the solid most likely arises from excimers because the corresponding excitation spectrum resembles the solution absorption profile (Figure S47). In contrast, the excitation spectra registered at the emission maxima of **7** and **9** in the solid state present red-shifted features in the 400–500 nm region relative to the respective solution absorption spectra (Figure S47), and therefore, these emissions are probably the result of molecular aggregates.

**Figure 9:**
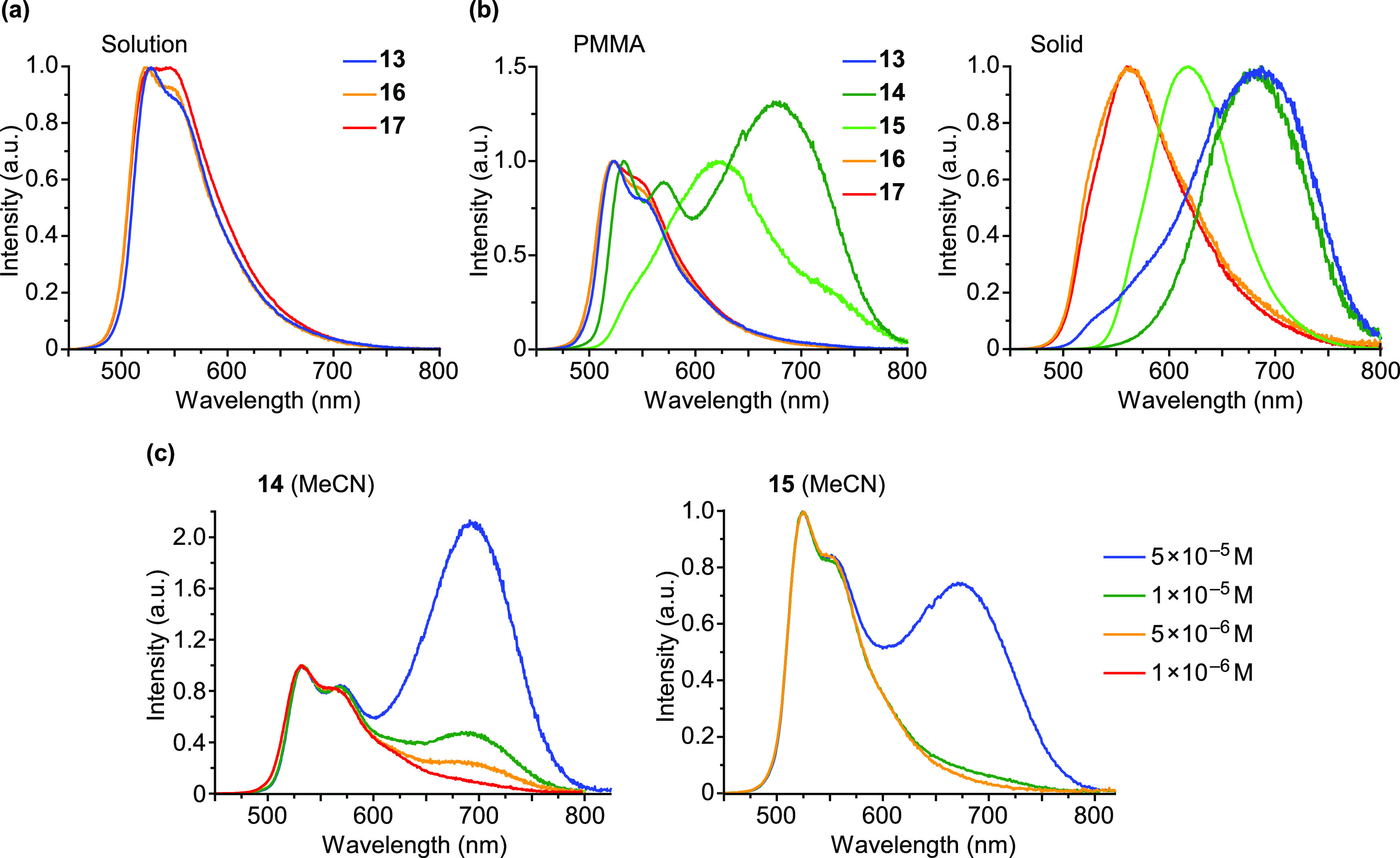
(a) Emission spectra of **13**, **16**, and **17** in solution (5 × 10^–5^ M) at 298 K; the solvent is acetone for **13** and MeCN for **16** and **17**. (b) Emission spectra of **13**–**17** in PMMA matrices (2 wt %) and solid state at 298 K. (c) Emission spectra of **14** and **15** in a MeCN solution at different concentrations at 298 K.

The emissions of the dmtppy derivatives **13**–**17** in solution show some vibronic structure, with the highest-energy peak in a narrow wavelength range (524–543 nm) (Figure 9). Therefore, for these derivatives, the variations due to the electronic properties of the ancillary ligand are much smaller and even operate in the opposite direction for the XyNC derivative **14**, whose emission appears slightly red-shifted with respect to the rest of complexes. This fact seems to indicate a higher predominance of the terdentate ligand orbitals in the emissive excited state compared to the dPhOppy derivatives. Complexes **14** and **15** show a second very broad and red-shifted emission centered at ca. 693 and 674 nm (MeCN) or 706 and 641 nm (MeTHF), respectively, that can be attributed to excimer formation. This was confirmed by registering the emission spectra at different concentrations in MeCN (Figure 9) and MeTHF (Figures S30 and S32), which show that the relative intensity of the low-energy band decreases upon dilution. The excimeric emission of **15** is particularly intense in MeTHF at a 5 × 10^–5^ M concentration, as observed for **9**. As a consequence, marked changes in emission color can be observed (see Figures S31 and S33 for photographs). The excitation spectra recorded at the low energy emission wavelengths were identical to the absorption spectra, which supports their excimeric nature.

The emission spectra of complexes **13**, **16**, and **17** in PMMA are very similar to those observed in solution and only the monomeric emission is observed (Figure 9b). In this medium, complex **14** shows a structured monomeric emission together with a significant proportion of a low energy emission (684 nm; Figure 9b), that can be attributed to the formation of excimers based on its similarity to the excimeric emission observed in solution and its excitation spectrum, which is similar to the excitation spectrum registered at the monomeric emission wavelength and matches the absorption spectrum in solution (Figure S45). In contrast, the main emission from derivative **15** in PMMA (622 nm) appears to be originated from aggregates because it is very different from the excimeric emission observed in solution and the corresponding excitation spectrum is red-shifted with respect to the absorption spectrum in solution (Figure S46); the shoulder at 539 nm is possibly a contribution from individual molecules, whereas the broad feature centered around 730 nm is probably a second aggregate emission. In the solid state, complexes **13**, **14**, and **15** exhibit almost exclusively low energy emissions. In the case of **13**, the lowest-energy feature of the corresponding excitation spectrum is similar to the absorption profile in solution (Figure S47), and therefore, we are inclined to ascribe an excimeric origin to its emission in the solid state. In contrast, the excitation spectra of **14** and **15** in the solid state present significantly red-shifted bands relative to the absorption spectra in solution (Figure S47), indicating emission from aggregates. The excitation and emission spectra of derivatives with NHC ligands **16** and **17** in the solid state are also red-shifted with respect to those in solution or PMMA, but to a lesser extent, suggesting a less significant aggregation effect.

The highest quantum yields (Φ) are attained for dPhOppy derivatives, with values up to 0.79 in solution, 0.84 in PMMA, and 0.48 in the solid state, while dmtppy derivatives reach up to 0.66 in solution and 0.71 in PMMA, and are poor emitters in the solid state, with the exception of **15** (Φ = 0.35). For complexes that easily form excimers in fluid solution, lifetimes were measured for the monomeric emission at the lowest concentration to avoid quenching caused by excimer formation. With the exception of **4**, the studied complexes gave monoexponential decay lifetimes in fluid solution ranging from 3 to 16 μs, which are typical of cyclometalated Pt(II) complexes and demonstrate triplet multiplicity of the emissive state. The biexponential decay observed for **4** in MeCN solution can probably be attributed to the presence of both monomeric and aggregate emissions. Biexponential decays were also observed for some of the complexes in PMMA matrix and all of them in the solid state, which can be attributed to the existence of different structural environments or the presence of the monomeric and aggregate or excimeric emissions.

### Luminescence at Low Temperature

**Figure 10:**
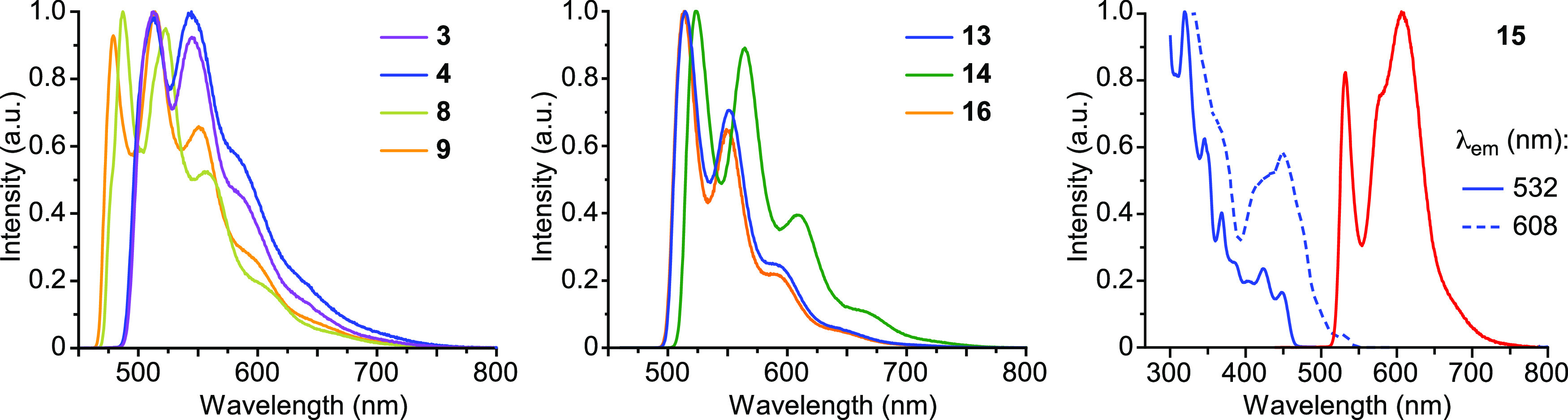
Emission spectra of selected complexes in MeTHF glasses at 77 K (5 × 10^–5^ M). In the case of **15**, the excitation spectra monitored at the two observed emission maxima are shown.

The emissions of **3**–**5**, **7**–**11**, and **13**–**17** were also examined in MeTHF glasses at 77 K. The resultant data are given in Table 2 and representative emission spectra are shown in Figure 10. The complete set of excitation and emission spectra is included in the Supporting Information. Highly structured emissions were observed for all of the studied complexes at a 5 × 10^–5^ M concentration, arising exclusively from monomeric species, except for **15**, which showed both a monomeric emission (λ_max_ = 532 nm) and a low energy emission (λ_max_ = 608 nm) that overlaps the lower energy vibronic peaks of the former. The variations in emission energies along the series generally reflect those observed at 298 K for the monomeric emissions in fluid solution. The fact that no emission from aggregates is observed for complexes **4** and **5** is attributable to a better solvation of the monomeric species by MeTHF compared to acetone. Notably, the low energy emissions observed for **7**, **8**, **9**, and **14** in fluid MeTHF at a 5 × 10^–5^ M concentration are absent in the emission spectra at 77 K, which is consistent with an excimeric behavior, because the probability of excimer formation due to collisions decreases as the temperature is lowered.^50^ The low energy emission observed for **15** at 77 K can be ascribed to the formation of aggregates because its excitation spectrum is red-shifted with respect to that of the monomeric emission (Figure 10); the most likely explanation for the observation of this emission is that the formation of dimeric assemblies is particularly favored for this complex, and the molecules may stabilize as dimers upon cooling. This effect has been recently observed for Pt(II) complexes bearing 1,3-di(2-pyridyl)benzene.^98^

The observed lifetimes at 77 K are significantly longer for derivatives with strong π acceptor ancillary ligands, particularly for CO, indicating a lower MLCT contribution to the emissive excited state. Complex **15** gives a biexponential decay at the aggregate emission maximum, whose major component is practically identical to the lifetime registered at the monomeric emission peak (23 μs), whereas the minor component is shorter (8.5 μs). This is compatible with overlapping monomeric and aggregate emissions.

### Assignment of Low Energy Emissions

The excimeric emissions of complexes **7** (in solution at 298 K), **8** (in solution and solid state at 298 K), **9** (in MeTHF solution at 298 K), **14** (in solution and PMMA at 298 K), and **15** (in solution at 298 K) have very similar characteristics that point to triplet metal–metal to ligand charge-transfer (^3^MMLCT) excited states involving both Pt···Pt contacts and π interactions between aromatic systems, as frequently observed for Pt(II) complexes with largely planar and sterically undemanding heteroaromatic ligands.^67^ Although the crystal structure of **7** does not show metallophilic Pt···Pt interactions, and those found for **9** in the crystalline state are rather long, they may be established or shortened in solution upon formation of excimers.^57^ These interactions must be facilitated by the low steric demand of the CO or BuNC ligands or the ability of the XyNC ligand to adopt a coplanar orientation relative to the metal coordination plane as opposed to the pyridine-based or NHC ancillary ligands. Notably, complexes **9** and **15** show the highest tendency to produce excimeric emissions in solution, consistent with the fact the CO ligand offers the least steric hindrance. A ^3^MMLCT state is also compatible with the higher energies of the excimeric emissions of **9** and **15** relative to those of the isocyanide complexes, which can be attributed to the lower energies of the metal orbitals in the CO derivatives.

The aggregate emissions of **4** and **5** in acetone cannot be conclusively explained on the basis of the available data. A fraction of the molecules in solution may be in the form of relatively stable ground-state dimers, wherein the frontier orbitals are altered with respect to the monomeric species. The incipient vibronic structure suggests that the emissive excited state is primarily centered on the dPhOppy ligand. In the case of **5**, the fact that no emission from monomeric species was observed in acetone solution indicates a very effective nonradiative decay, which would be consistent with the low quantum yields of the monomeric emissions in PMMA and the solid state.

The emissions of **4** and **13** in the solid state are characterized by very low energies and poor quantum yields. ^3^MMLCT states can be ruled out as the origin of these emissions because the dimeric assemblies observed in the crystal structures present very long metal–metal distances and do not show π interactions between aromatic systems. Instead, they are held together by intermolecular C–H···Pt and C–H···O or C–H/π hydrogen bonds. A conceivable explanation is the formation of intermolecular charge-transfer excited states, possibly involving an electronic transition from occupied orbitals of the metal and the terdentate ligand of one molecule to a π* orbital of the pyridine-based ligand of the other molecule.

The aggregate emission of **7** in the solid state can be attributed to the π interactions between aromatic systems of the dPhOppy ligand that are observed in the crystal structure, which is a common phenomenon for Pt(II) complexes with planar heteroaromatic ligands.^99,100^ In contrast, the aggregate emissions of **9**, **14**, and **15** in the solid state are attributable to ^3^MMLCT excited states on the basis of the Pt···Pt contacts and π interactions observed in their crystal structures; their energies are similar to those of the excimeric emissions in solution. The same assignment fits the main aggregate emission of **15** in PMMA (622 nm) and the low energy emission observed in glassy MeTHF at 77 K (608 nm).

The aggregate emission of **9** in MeCN solution has a higher energy as compared to the excimeric emission observed in MeTHF or the aggregate emission in the solid state, pointing to a different type of molecular assembly that causes a less significant alteration of frontier molecular orbitals, although its nature cannot be established.

### Electrochemistry

The redox properties were examined by means of cyclic voltammetry in MeCN solution for those complexes that are stable and soluble enough in this solvent, namely, **8**, **10**, **11**, **16**, and **17**, and the voltammograms are displayed in Figure S50. The potentials of the observed reduction or oxidation processes and energy estimations for the highest occupied/lowest unoccupied molecular orbital (HOMO/LUMO) are given in Table 3. An irreversible oxidation wave is observed in all cases in the range from 0.68 to 0.86 V vs saturated calomel electrode (SCE). The dPhOppy derivatives present very similar potentials for this oxidation, which are somewhat more positive as compared with those of the dmtppy derivatives, indicating that the HOMO is mainly on the N∧C∧C ligand and has a lower energy for the dPhOppy complexes.

**Table 3:** Electrochemical Data^a^ and HOMO/LUMO Energy Estimations^b^ for Complexes **8**, **10**, **11**, **16**, and **17**

complex	*E*_pa_^c^	*E*_pc_^d^	*E*_1/2_^e^	*E*_HOMO_	*E*_LUMO_	Δ*E*_HOMO–LUMO_
**8**	0.82	–2.31, –2.60	–1.80	–5.42	–2.97	2.45
**10**	0.84	–2.60	–2.01	–5.46	–2.76	2.70
**11**	0.86	–2.13, –2.28		–5.35	–2.74	2.61
**16**	0.75	–2.68	–2.09	–5.34	–2.69	2.65
**17**	0.68	–2.21, –2.28, –2.24		–5.22	–2.60	2.62

a In V vs SCE, registered in a 0.1 M solution of (Bu_4_N)PF_6_ in dry MeCN at 100 mV s^–1^.

b In eV.

c Irreversible anodic peak potentials.

d Irreversible cathodic peak potentials.

e For the reversible waves.

The first reduction is reversible for the XyNC complex **8** and the imz derivatives **10** and **16**, whereas it is irreversible for the trz derivatives **11** and **17**. The potentials of this reduction appear significantly influenced by the ancillary ligand, being less negative for the strongest π acceptor (XyNC). Therefore, the LUMO possibly has an important contribution from ancillary ligand orbitals.

### Computational Study

**Figure 11:**
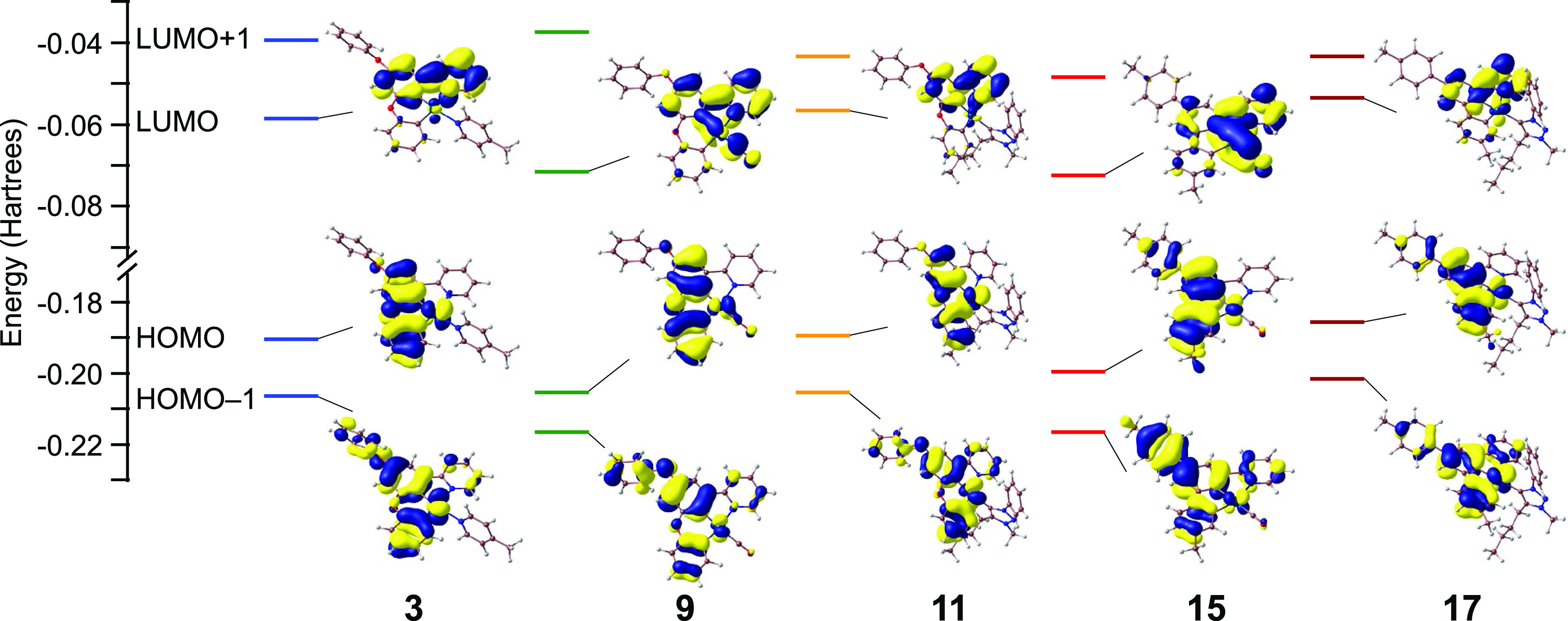
Frontier orbital energy diagrams and selected isosurfaces (0.03 e bohr^–3^) for complexes **3**, **9**, **11**, **15**, and **17** from DFT calculations.

For a better understanding of their photophysical properties, DFT and TD-DFT calculations were performed for a selected set of complexes, namely, **3**, **9**, **11**, **15**, and **17**. Details are presented in the Supporting Information. Frontier orbital energies and selected isosurfaces are shown in Figure 11. In all cases, the HOMO and the HOMO–1 are π orbitals essentially distributed over the dimetalated biphenyl or PhOPh portion of the dmtppy or dPhOppy ligand, respectively. There is an important metal d orbital contribution to these orbitals, which is mainly dictated by the ancillary ligand and is much higher for the γ-picoline and trz complexes (range 17.8–29.5%) than for the carbonyl complexes (range 5.4–6.3%), reasonably because of the considerably stronger π acceptor character of the CO ligand. This fact also explains the lower HOMO and HOMO–1 energies found for the carbonyl complexes. The lowest unoccupied molecular orbital (LUMO) is a π* orbital mainly located on the central phenyl and pyridyl ring. This orbital has a significant contribution from a CO π* orbital for complexes **9** and **15** and has a lower energy relative to the rest of calculated complexes; as a consequence, the calculations do not predict significant variations of the HOMO–LUMO energy gaps along the series.

The TD-DFT calculations show that the lowest-energy singlet excitation (S_1_) corresponds in all cases to a HOMO–LUMO transition, and therefore it can be described as having a mixed intraligand charge-transfer/metal-to-ligand charge-transfer character (ILCT/MLCT). In the cases of **9** and **15**, there is additionally a ligand–ligand charge-transfer (LLCT) contribution, due to the participation of CO orbitals in the LUMO. The next singlet excitation with significant oscillator strength corresponds to a HOMO–1-LUMO transition for derivatives **3** (S_2_), **9** (S_2_), **11** (S_3_), and **15** (S_2_) and thus also has a mixed ILCT/MLCT character (ILCT/MLCT/LLCT for CO derivatives). For **17**, on the other hand, it is a HOMO–LUMO+2 transition (S_4_), although it can also be designated as ILCT/MLCT. The two mentioned excitations should correspond to the two lowest-energy bands observed in the absorption spectra. A comparison of the energies calculated by TD-DFT with those obtained from the maxima of the experimentally observed bands is shown in Table 4. In general, the calculations make a good prediction of the energy of the lowest-energy band with a maximum deviation of 0.21 eV for complex **9**. Larger deviations are observed for the second band, whose energy is overestimated in the cases of complexes **3**, **11**, and **17** (up to 0.27 eV).

**Table 4:** Energies and Wavelengths of the Two Most Intense, Lowest Singlet Excitations from TD-DFT Calculations Compared with the Experimental Band Maxima

		Δ*E* (eV) (λ (nm))	
complex	state	calcd	exp.
**3**	S_1_	2.93 (423)	2.98 (416)
	S_2_	3.45 (360)	3.25 (382)
**9**	S_1_	3.09 (402)	3.30 (376)
	S_2_	3.43 (361)	3.60 (344)
**11**	S_1_	2.93 (423)	2.92 (425)
	S_3_	3.46 (358)	3.19 (389)
**15**	S_1_	2.84 (437)	2.94 (421)
	S_2_	3.33 (372)	3.38 (367)
**17**	S_1_	3.02 (411)	2.85 (435)
	S_4_	3.45 (360)	3.21 (386)

The lowest triplet vertical excitation (T_1_) corresponds in all cases to a HOMO–LUMO transition. Therefore, the emissive excited state can be designated as ^3^ILCT/MLCT, with an additional LLCT contribution for the CO derivatives. Its geometry could be optimized for all of the calculated complexes. The electronic energies of the optimized T_1_ states with respect to the respective optimized ground states (adiabatic energy differences) are 2.38 eV (**3**, 522 nm), 2.54 eV (**9**, 488 nm), 2.38 eV (**11**, 522 nm), 2.28 eV (**15**, 544 nm), and 2.44 eV (**17**, 509 nm), and provide a good prediction of the monomeric emission energies, with a maximum deviation of 0.13 eV (complex **11**).

## Conclusions

Two series of complexes of the type [Pt(N∧C∧C)(L)] have been synthesized with dPhOppy and dmtppy ligands. The ancillary ligands with a stronger π-acceptor character, such as CO and isocyanides as well as N-heterocyclic carbenes, give rise to the most stable complexes in solution. In addition to affecting their stability, these ligands determine the formation of different types of molecular assemblies in the solid state. Thus, complexes with less bulky ligands, such as CO and isocyanides, favor the formation of bimolecular assemblies through π interactions between the aromatic rings, metallophilic Pt···Pt contacts, or a combination of both. However, in the cases of the complexes [Pt(dPhOppy)(py-CHO-4)] and [Pt(dmtppy)(γ-picoline)], bimolecular assemblies have been observed that seem to be largely driven by different types of hydrogen bonds, including C–H···O, C–H···Pt, or C–H/π.

A photophysical study on the stable Pt(II) complexes has shown that most of them can produce efficient luminescence from ^3^ILCT/MLCT excited states in solution, PMMA matrices, or in the solid state at room temperature. For dPhOppy complexes, the ancillary ligand affects the energy of the monomeric emissions, which increase as its π acceptor character increases, whereas with the dmtppy ligand, the variations are less significant. Excimeric emissions have been demonstrated for derivatives with CO or isocyanides as ancillary ligands in fluid solutions and, in certain cases, in PMMA or in the solid state, which can be assigned to ^3^MMLCT states. In addition, emissive aggregates or assemblies have been observed in solution, PMMA, and the solid state, some of which involve metallophilic Pt···Pt and/or π interactions between aromatic systems, whereas others are held together by different noncovalent interactions.

In summary, a new class of strongly luminescent Pt(II) compounds has been demonstrated, which show great versatility and potential for further developments thanks to the possibilities of luminescence modulation through the variation of the ancillary ligand and the formation of different types of molecular assemblies.

## Supporting Information Available

Experimental details and characterization data, X-ray structure determinations, NMR spectra of new compounds, additional photophysical data, cyclic voltammograms, computational methods, and details (PDF)
