# Supplementary material for: Luminescent Platinum(II) Complexes with Terdentate N∧C∧C Ligands
Source: Inorg Chem. 2023 Dec 5;62(51):20987–1002. doi: 10.1021/acs.inorgchem.3c02399 (PMC10751801; doi:10.1021/acs.inorgchem.3c02399)
Supplement: Supplementary file 1 — ic3c02399_si_001.pdf [file ic3c02399_si_001.pdf]

**SUPPORTING INFORMATION**

# Luminescent Platinum(II) Complexes with Terdentate N^C^C Ligands

*Dionisio Poveda,*[†] *Ángela Vivancos,*[*,†] *Delia Bautista*[‡] *and Pablo González-Herrero*[*,†]

[†]Departamento de Química Inorgánica, Facultad de Química, Universidad de Murcia, Campus de Espinardo, 19, 30100 Murcia, Spain.

[‡]Área Científica y Técnica de Investigación, Universidad de Murcia, Campus de Espinardo, 21, 30100 Murcia, Spain.

*E-mail: angela.vivancos@um.es, pgh@um.es.

**Contents:**

1. Experimental details and characterization data ........ 2
   1.1. General considerations and materials ........ 2
   1.2. Spectroscopic and analytical methods ........ 2
   1.3. Synthesis and characterizaction data of new complexes ........ 2
   1.4. NMR spectra of new compounds ........ 8
   1.5. Ligand exchange equilibrium of **3** in $CD_3CN$ ........ 22
   1.6. X-ray structure determinations ........ 23
2. Additional photophysical data ........ 34
3. Electrochemical measurements ........ 52
4. Computational methods and data ........ 53
   4.1. Complex [Pt(dPhOppy)(γ-pic)] (**3**) ........ 54
   4.2. Complex [Pt(dPhOppy)(CO)] (**9**) ........ 57
   4.3. Complex [Pt(dPhOppy)(trz)] (**11**) ........ 60
   4.4. Complex [Pt(dmtppy)(CO)] (**15**) ........ 63
   4.5. Complex [Pt(dmtppy)(trz)] (**17**) ........ 67
5. References ........ 77

# 1. Experimental details and characterization data

## 1.1. General considerations and materials

All reactions were carried out at room temperature under an inert atmosphere. Synthesis-grade solvents were obtained from commercial sources. Acetone was deoxygenated by bubbling nitrogen. [$PtCl_2(NCPh)_2$],[1] 2-(3,5-diphenoxyphenyl)pyridine (dPhOppyH),[2] $Bu_4N$[PtCl(dmtppy)],[3] and 4-butyl-3-methyl-1-phenyl-1*H*-1,2,3-triazolium iodide[4] were prepared according to reported methods. All other reagents were obtained from commercial sources and used without further purification. The experimental setup for the photochemical reactions has been previously described.[3] The irradiation sources were LED Engin LuxiGen™ LZ1-10UB0R-00U8 ($\lambda_{max}$ = 405 nm) and New Energy LST1-01G01-RYL1-00 ($\lambda_{max}$ = 450 nm) LED emitters.

## 1.2. Spectroscopic and analytical methods

NMR spectra were recorded on Bruker Avance 300, 400, or 600 MHz spectrometers at 298 K. Chemical shifts are referred to residual signals of non-deuterated solvents and are given in ppm downfield from tetramethylsilane. Elemental analyses were carried out with a LECO CHNS-932 microanalyzer. UV-vis absorption and diffuse reflectance spectra were registered on a Perkin-Elmer Lambda 750S spectrophotometer, equipped with a 60 mm integrating sphere; for reflectance measurements, nujol mulls of solid samples between polyethylene sheets were employed. Excitation and emission spectra were registered on a Jobin Yvon Fluorolog 3-22 spectrofluorometer. Emission spectra were corrected for detector sensitivity. The measurements in solution were carried out in a right-angle configuration using 10 mm quartz fluorescence cells (298 K). The emission data in PMMA matrix were measured in a front-face configuration, using quartz slides as sample holders. Emission lifetimes were determined using an IBH FluoroHub controller in MCS mode and the Fluorolog's FL-1040 phosphorimeter pulsed xenon lamp as excitation source ($\tau > 12$ μs ) or in TCSPC mode using a pulsed NanoLED source ($\tau \leq 12$ μs ); the estimated uncertainty is ±10% or better. Emission quantum yields were determined using a Hamamatsu C11347 Absolute PL Quantum Yield Spectrometer; the estimated uncertainty is ±5% or better. Emission lifetimes and quantum yields in solution and PMMA matrices were registered under rigorous exclusion of oxygen.

## 1.3. Synthesis and characterizaction data of new complexes

**$Pr_4$N[PtCl(dPhOppy)] (2).** A Carius tube was charged with [$PtCl_2(NCPh)_2$] (100 mg, 0.21 mmol), dPhOppyH (79 mg, 0.23 mmol), $Na_2CO_3$ (45 mg, 0.42 mmol) and acetone (8 mL). The mixture was irradiated with violet light under vigorous stirring for 16 h. The solvent was removed under reduced pressure and the residue was extracted with $CH_2Cl_2$ (20 mL) and filtered through a silica pad. The yellow filtrate was concentrated (10 mL) and a solution of $Pr_4NCl$ (56 mg, 0.25 mmol) in acetone (2 mL) was added. After stirring for 1 h, partial evaporation of the solvent (2 mL) and slow addition of $Et_2O$ (20 mL) led to the precipitation of an orange solid, which was collected by filtration, washed with $Et_2O$ (3 × 4 mL) and vacuum-dried to give **2**. Yield: 91 mg, 57%. $^1$H NMR (600 MHz, $CD_2Cl_2$): δ 10.11 (br d with satellites, $J_{H\text{-}H}$ ~ 5.4 Hz, $J_{H\text{-}Pt}$ ~ 13 Hz, 1H), 8.99 (dd with satellites, $J_{H\text{-}H}$ = 7.9, 1.7 Hz, $J_{H\text{-}Pt}$ = 67 Hz, 1H), 7.76 (td, $J_{H\text{-}H}$ = 7.6, 1.7 Hz, 1H), 7.66 (br d, $J_{H\text{-}H}$ ~ 8.0 Hz, 1H), 7.37 – 7.28 (m, 2H), 7.23 (ddd, $J_{H\text{-}H}$ = 7.1, 5.5, 1.4 Hz, 1H), 7.10 (d, $J_{H\text{-}H}$ = 2.5 Hz, 1H), 7.06 (br t, $J_{H\text{-}H}$ = 7.5 Hz, 1H), 7.05 – 7.00 (m, 2H), 6.95 (td, $J_{H\text{-}H}$ = 7.2, 1.7 Hz, 1H), 6.93 (td, $J_{H\text{-}H}$ = 7.9, 1.8 Hz, 1H), 6.71 (ddd, $J_{H\text{-}H}$ = 8.1, 6.7, 2.0 Hz, 1H), 6.69 (d, $J_{H\text{-}H}$ = 2.2 Hz, 1H), 2.89 – 2.72 (m, 8H), 1.39 – 1.19 (m, 8H), 0.70 (t, $J_{H\text{-}H}$ = 7.4 Hz, 12H). $^{13}C\{^1H\}$ APT NMR (151 MHz, $CD_2Cl_2$): δ 163.9 (C), 158.7 (C), 153.3 (C), 152.9 (C), 152.0 (CH), 151.7 (C), 147.2 (C), 145.6 (CH), 137.5 (CH), 130.0 (2CH), 123.6 (CH), 123.1 (CH), 122.7 ($J_{C\text{-}Pt}$ ~ 17 Hz, CH), 120.5 (CH), 119.6 (C), 118.6 (CH), 118.1 (2CH),

117.4 (C), 115.2 ($J_{C-Pt}$ ~ 26 Hz, CH), 110.2 (CH), 109.9 ($J_{C-Pt}$ = 31 Hz, CH), 60.5 (4$CH_2$), 15.9 (4$CH_2$), 10.6 (4$CH_3$). Anal. Calcd for $C_{35}H_{43}ClN_2O_2Pt$: C, 55.73; H, 5.75; N, 3.71. Found: C, 55.78; H, 5.88; N, 3.58.

**[Pt(dPhOppy)(γ-pic)] (3).** The synthesis of this complex has been previously reported by us using $(Bu_4N)_2[Pt_2Cl_6]$ as precursor.[3] The following is an alternative procedure: A Carius tube was charged with $[PtCl_2(NCPh)_2]$ (100 mg, 0.21 mmol), dPhOppyH (79 mg, 0.23 mmol), $Na_2CO_3$ (45 mg, 0.42 mmol) and acetone (8 mL). The mixture was irradiated with violet light under vigorous stirring for 16 h. The resulting suspension was treated with γ-picoline (53 µL, 0.53 mmol) and stirred for 30 min. The solvent was removed under reduced pressure, the residue was extracted with $CH_2Cl_2$ (10 mL) and the suspension was filtered through a silica pad. The yellow filtrate was concentrated (2 mL) and MeOH (20 mL) was added, whereupon a yellow solid precipitated, which was collected by filtration, washed with MeOH (4 × 5 mL) and vacuum-dried to give **3**. Yield: 81 mg, 61%. $^1$H NMR data are agreement with those previously reported.[3]

**[Pt(dPhOppy)(py-CHO-4)] (4).** This complex was obtained as an orange solid as described for **3**, from $[PtCl_2(NCPh)_2]$ (100 mg, 0.21 mmol), dPhOppyH (79 mg, 0.23 mmol), $Na_2CO_3$ (45 mg, 0.42 mmol) and 4-pyridinecarboxaldehyde (50 µL, 0.53 mmol). $Et_2O$ was employed for the final precipitation. Yield: 79 mg, 58%. $^1$H NMR (600 MHz, $CD_2Cl_2$): δ 10.23 (s, 1H), dd with satellites, $J_{H-H}$ = 6.4, 1.7 Hz, $J_{H-Pt}$ ~ 16 Hz, 2H), 7.98 (dd, $J_{H-H}$ = 6.4, 1.7 Hz, 2H), 7.81 (ddd, $J_{H-H}$ = 8.0, 7.2, 1.6 Hz, 1H), 7.78 (br d, $J_{H-H}$ ~ 8.0 Hz, 1H), 7.38 (ddd with satellites, $J_{H-H}$ = 5.6, 1.6, 0.8 Hz, $J_{H-Pt}$ ~ 16 Hz, 1H), 7.37 – 7.32 (m, 2H), 7.21 (d, $J_{H-H}$ = 2.2 Hz, 1H), 7.18 – 7.05 (m, 4H), 7.05 – 7.00 (m, 2H), 6.82 (d with satellites, $J_{H-H}$ = 2.2 Hz, $J_{H-Pt}$ = 27 Hz, 1H), 6.59 (ddd, $J_{H-H}$ = 7.7, 1.4, 1.1 Hz, 1H), 6.58 (ddd with satellites, $J_{H-H}$ = 7.7, 2.3, 0.5 Hz, $J_{H-Pt}$ ~ 72 Hz, 1H). $^{13}$C{$^1$H} APT NMR (151 MHz, $CD_2Cl_2$): δ 193.3 (CH), 164.2 (C), 158.6 (C), 154.7 (C), 154.3 (2CH), 153.8 (C), 153.2 (C), 147.8 (C), 147.2 (CH), 142.3 (C), 138.5 (CH), 138.0 ($J_{C-Pt}$ ~ 19 Hz, CH), 130.1 (2CH), 125.5 ($J_{C-Pt}$ ~ 19 Hz, 2CH), 124.7 (CH), 123.6 (CH), 123.0 (CH), 120.8 ($J_{C-Pt}$ = 51 Hz, CH), 120.0 ($J_{C-Pt}$ ~ 21 Hz, CH), 118.4 (2CH), 116.3 (CH), 116.2 (C), 116.0 (C), 110.8 ($J_{C-Pt}$ ~ 36 Hz, CH), 109.7 ($J_{C-Pt}$ ~ 21 Hz, CH). Anal. Calcd for $C_{29}H_{20}N_2O_3Pt$: C, 54.46; H, 3.15; N, 4.38. Found: C, 54.56; H, 3.32; N, 4.23.

**[Pt(dPhOppy)(PPh$_3$)] (5).** This complex was obtained as a yellow solid as described for **3**, from $[PtCl_2(NCPh)_2]$ (100 mg, 0.21 mmol), dPhOppyH (79 mg, 0.23 mmol), $Na_2CO_3$ (45 mg, 0.42 mmol) and $PPh_3$ (83 mg, 0.32 mmol). Yield: 101 mg, 60%. $^1$H NMR (600 MHz, $CD_2Cl_2$): δ 7.88 (ddd with satellites, $J_{H-H}$ = 5.8, 1.1, 0.6 Hz, $J_{H-Pt}$ ~ 17 Hz, 1H), 7.84 – 7.77 (m, 6H), 7.76 (ddd, $J_{H-H}$ = 8.5, 1.4, 0.7 Hz, 1H), 7.71 (ddd, $J_{H-H}$ = 8.1, 7.3, 1.6 Hz, 1H), 7.45 – 7.39 (m, 3H), 7.39 – 7.32 (m, 8H), 7.26 (br dd, $J_{H-H}$ ~ 2.2 Hz, $J_{H-P}$ ~ 1.4 Hz, 1H), 7.21 (ddd with satellites, $J_{H-H}$ = 7.8, 1.7 Hz, $J_{H-P}$ = 1.7 Hz, $J_{H-Pt}$ ~ 82 Hz, 1H), 7.15 – 7.07 (m, 3H), 7.03 (dd with satellites, $J_{H-H}$ = 7.9, 1.4 Hz, $J_{H-Pt}$ ~ 32 Hz, 1H), 6.95 (dd with satellites, $J_{H-P}$ ~ 2.6 Hz, $J_{H-H}$ ~ 2.2 Hz, $J_{H-Pt}$ ~ 26 Hz, 1H), 6.81 (ddd, $J_{H-H}$ = 7.9, 6.9, 1.7 Hz, 1H), 6.64 (ddd, $J_{H-H}$ = 7.3, 5.7, 1.5 Hz, 1H), 6.15 (ddd, $J_{H-H}$ = 7.7, 6.9, 1.5 Hz, 1H). $^{13}$C{$^1$H} APT NMR (151 MHz, $CD_2Cl_2$): δ 166.5 (d, $J_{C-P}$ = 6.6 Hz, C), 158.4 (C), 155.7 (C), 154.1 ($J_{C-Pt}$ ~ 20 Hz, C), 153.5 (C), 152.2 (d, $J_{C-P}$ = 3.6 Hz, CH), 149.2 (C), 145.4 (d, $J_{C-P}$ = 16.3 Hz, CH), 138.0 (CH), 137.4 (d, $J_{C-P}$ = 104.9 Hz, C), 135.4 (d, $J_{C-P}$ = 12.2 Hz, 6CH), 132.9 (d, $J_{C-P}$ = 42.0 Hz, 3C), 130.7 (3CH), 130.1 (2CH), 128.9 (d, $J_{C-P}$ = 9.8 Hz, 6CH), 124.3 (CH), 123.2 (2CH), 120.8 (d with satellites, $J_{C-P}$ = 3.0 Hz, $J_{C-Pt}$ = 66 Hz, CH), 120.0 ($J_{C-Pt}$ ~ 21 Hz, CH), 118.7 (2CH), 116.2 ($J_{C-Pt}$ = 28 Hz, CH), 114.2 (d, $J_{C-P}$ = 5.8 Hz, C), 110.4 (d with satellites, $J_{C-P}$ = 4.9 Hz, $J_{C-Pt}$ = 34 Hz, CH), 109.6 ($J_{C-Pt}$ ~ 20 Hz, CH). $^{31}$P{$^1$H} NMR (243 MHz, $CD_2Cl_2$): δ 33.74 (s with satellites, $J_{P-Pt}$ = 2446 Hz, 1P). Anal. Calcd for $C_{41}H_{30}NO_2PPt$: C, 61.96; H, 3.80; N, 1.76. Found: C, 61.93; H, 3.71; N, 1.79.

**[PtCl$_2$(dPhOppy)(PPh$_3$)] (6).** A solution of [Pt(dPhOppy)(PPh$_3$)] (50 mg, 0.06 mmol) in $CHCl_3$ (10 mL) was irradiated with blue LEDs under atmospheric conditions and vigorous stirring for 24 h. The solvent was removed under reduced pressure and the residue was extracted with $CH_2Cl_2$ (25 mL) and filtered through

Celite. The yellow filtrate was concentrated (5 mL) and $Et_2O$ (30 mL) was added, whereupon a yellow solid precipitated, which was collected by filtration, washed with $Et_2O$ (4 × 3 mL) and vacuum-dried to give **6**. Yield: 30 mg, 56%. $^1H$ NMR (600 MHz, $CD_2Cl_2$): δ 9.41 (br d with satellites, $J_{H-H}$ ~ 5.5 Hz, $J_{H-Pt}$ ~ 11 Hz, 1H), 8.67 (dd with satellites, $J_{H-H}$ = 8.1, 1.6 Hz, $J_{H-Pt}$ = 37 Hz, 1H), 7.82 (td, $J_{H-H}$ = 7.6, 1.6 Hz, 1H), 7.78 (br d, $J_{H-H}$ ~ 7.9 Hz, 1H), 7.48 – 7.32 (m, 5H), 7.26 (d, $J_{H-H}$ = 2.5 Hz, 1H), 7.25 (ddd, $J_{H-H}$ = 7.4, 5.9, 1.5 Hz, 1H), 7.22 – 7.14 (m, 13H), 7.13 – 7.07 (m, 3H), 6.97 (ddd, $J_{H-H}$ = 8.4, 6.9, 1.6 Hz, 1H), 6.76 (dd with satellites, $J_{H-H}$ = 8.0, 1.6 Hz, $J_{H-Pt}$ ~ 16 Hz, 1H), 6.33 (d with satellites, $J_{H-H}$ = 2.4 Hz, $J_{H-Pt}$ = 16 Hz, 1H). $^{31}P\{^1H\}$ NMR (243 MHz, $CD_2Cl_2$): δ − 8.16 (s with satellites, $J_{P-Pt}$ = 2630 Hz, 1P). An appropriate $^{13}C\{^1H\}$ NMR spectrum could not be recorded due the poor solubility of this product. Anal. Calcd for $C_{41}H_{30}Cl_2NO_2PPt$: C, 56.89; H, 3.49; N, 1.62. Found: C, 56.96; H, 3.58; N, 1.47.

**[Pt(dPhOppy)(CN$^n$Bu)] (7).** This complex was obtained as a yellow solid as described for **3**, from [$PtCl_2(NCPh)_2$] (100 mg, 0.21 mmol), dPhOppyH (79 mg, 0.23 mmol), $Na_2CO_3$ (45 mg, 0.42 mmol) and *n*-butyl isocyanide (33 µL, 0.32 mmol). Yield: 88 mg, 67%. $^1H$ NMR (600 MHz, $CD_2Cl_2$): δ 8.87 (ddd with satellites, $J_{H-H}$ = 5.5, 1.7, 0.8 Hz, $J_{H-Pt}$ ~ 18 Hz, 1H), 8.03 (dd with satellites, $J_{H-H}$ = 7.6, 1.6 Hz, $J_{H-Pt}$ = 91 Hz, 1H), 7.91 (ddd, $J_{H-H}$ = 8.1, 7.4, 1.6 Hz, 1H), 7.80 (ddd, $J_{H-H}$ = 8.5, 1.3, 0.7 Hz, 1H), 7.41 – 7.30 (m, 2H), 7.25 (dd, $J_{H-H}$ = 7.3, 5.5, 1.3 Hz, 1H), 7.23 (br d, $J_{H-H}$ ~ 2.2 Hz, 1H), 7.18 (dd, $J_{H-H}$ = 8.0, 1.6 Hz, 1H), 7.14 (ddd, $J_{H-H}$ = 8.1, 6.8, 1.6 Hz, 1H), 7.11 (tdd, $J_{H-H}$ = 7.4, 1.1, 1.0 Hz, 1H), 7.09 – 7.06 (m, 2H), 6.90 (d with satellites, $J_{H-H}$ = 2.1 Hz, $J_{H-Pt}$ = 23 Hz, 1H), 6.82 (ddd, $J_{H-H}$ = 7.6, 6.8, 1.6 Hz, 1H), 3.80 (t, $J_{H-H}$ = 6.8 Hz, 2H), 2.00 – 1.90 (m, 2H), 1.69 – 1.60 (m, 2H), 1.05 (t, $J_{H-H}$ = 7.4 Hz, 3H). $^{13}C\{^1H\}$ APT NMR (151 MHz, $CD_2Cl_2$): δ 165.0 (C), 158.4 (C), 156.2 (C), 153.7 (C), 152.8 (C), 151.6 (CH), 150.4 (C), 149.8 (C), 146.6 (CH), 138.7 (CH), 132.6 (C), 130.2 (2CH), 125.5 (CH), 124.4 (CH), 123.3 (CH), 121.6 ($J_{C-Pt}$ = 62 Hz, CH), 120.3 ($J_{C-Pt}$ ~ 19 Hz, CH), 118.9 (2CH), 116.7 ($J_{C-Pt}$ = 26 Hz, CH), 113.4 (C), 110.4 ($J_{C-Pt}$ = 32 Hz, CH), 109.3 ($J_{C-Pt}$ ~ 18 Hz, CH), 44.7 ($CH_2$), 31.3 ($CH_2$), 20.3 ($CH_2$), 13.5 ($CH_3$). Anal. Calcd for $C_{28}H_{24}N_2O_2Pt$: C, 54.63; H, 3.93; N, 4.55. Found: C, 54.53; H, 3.96; N, 4.58.

**[Pt(dPhOppy)(CNXy)] (8).** This complex was obtained as a yellow solid as described for **3**, from [$PtCl_2(NCPh)_2$] (100 mg, 0.21 mmol), dPhOppyH (79 mg, 0.23 mmol), $Na_2CO_3$ (45 mg, 0.42 mmol) and 2,6-dimethylphenyl isocyanide (36 mg, 0.28 mmol). Yield: 85 mg, 60%. $^1H$ NMR (600 MHz, $CD_2Cl_2$): δ 9.02 (dd with satellites, $J_{H-H}$ = 5.6, 1.7, 0.8 Hz, $J_{H-Pt}$ ~ 21 Hz, 1H), 8.20 (dd with satellites , $J_{H-H}$ = 7.6, 1.7 Hz, $J_{H-Pt}$ = 91 Hz, 1H), 7.94 (ddd, $J_{H-H}$ = 8.2, 7.4, 1.6 Hz, 1H), 7.86 (br d, $J_{H-H}$ ~ 8.2 Hz, 1H), 7.43 – 7.31 (m, 3H), 7.29 (d, $J_{H-H}$ = 2.2 Hz, 1H), 7.28 – 7.18 (m, 4H), 7.16 (ddd, $J_{H-H}$ = 8.1, 6.9, 1.7 Hz, 1H), 7.12 (tdd, $J_{H-H}$ = 7.4, 1.1, 1.0 Hz, 1H), 7.12 – 7.04 (m, 2H), 6.95 (d with satellites, $J_{H-H}$ = 2.1 Hz, $J_{H-Pt}$ = 23 Hz, 1H), 6.81 (ddd, $J_{H-H}$ = 7.7, 6.9, 1.5 Hz, 1H), 2.60 (s, 6H). $^{13}C\{^1H\}$ APT NMR (151 MHz, $CD_2Cl_2$): δ 165.0 (C), 160.5 (C), 158.2 (C), 156.4 (C), 153.7 ($J_{C-Pt}$ ~ 22 Hz, C), 152.6 (C), 151.6 ($J_{C-Pt}$ ~ 14 Hz, CH), 150.5 (C), 147.1 (CH), 138.9 (CH), 135.8 (2C), 133.0 (C), 130.2 (2CH), 129.6 (CH), 128.7 (2CH), 127.8 (C), 125.7 (CH), 124.5 (CH), 123.4 (CH), 121.7 ($J_{C-Pt}$ = 62 Hz, CH), 120.4 ($J_{C-Pt}$ ~ 21 Hz, CH), 118.9 (2CH), 116.8 ($J_{C-Pt}$ = 27 Hz, CH), 113.3 (C), 110.4 ($J_{C-Pt}$ = 31 Hz, CH), 109.2 ($J_{C-Pt}$ ~ 15 Hz, CH), 19.4 ($2CH_3$). Anal. Calcd for $C_{23}H_{24}N_2O_2Pt$: C, 57.92; H, 3.65; N, 4.22. Found: C, 57.99; H, 3.70; N, 4.21.

**[Pt(dPhOppy)(CO)] (9).** A Carius tube was charged with [$PtCl_2(NCPh)_2$] (100 mg, 0.21 mmol), dPhOppyH (79 mg, 0.23 mmol), $Na_2CO_3$ (45 mg, 0.42 mmol) and acetone (8 mL). The mixture was irradiated with violet light under vigorous stirring for 16 h. CO was bubbled through the mixture for 5 min and the mixture was stirred for 2 h. The solvent was removed under reduced pressure and the residue was extracted with $CH_2Cl_2$ (100 mL) and filtered through Celite. The filtrate was concentrated (2 mL) and AcOEt (20 mL) was added. The yellow precipitate was collected by filtration and washed with AcOEt (3 × 5 mL), $Et_2O$ (3 × 5 mL) and vacuum-dried to give **9**. Yield: 68 mg, 57%. $^1H$ NMR (600 MHz, $CD_2Cl_2$): δ 8.79 (ddd with

satellites, $J_{H\text{-}H}$ = 5.6, 1.6, 0.8 Hz, $J_{H\text{-}Pt}$ ~ 20 Hz, 1H), 7.99 (ddd, $J_{H\text{-}H}$ = 8.1, 7.5, 1.6 Hz, 1H), 7.95 (dd with satellites, $J_{H\text{-}H}$ = 7.6, 1.6 Hz, $J_{H\text{-}Pt}$ = 96 Hz, 1H), 7.86 (br d, $J_{H\text{-}H}$ ~ 8.0 Hz, 1H), 7.45 – 7.35 (m, 2H), 7.33 (ddd, $J_{H\text{-}H}$ = 7.5, 5.5, 1.4 Hz, 1H), 7.30 – 7.27 (m, 2H), 7.24 (ddd, $J_{H\text{-}H}$ = 8.1, 6.9, 1.6 Hz, 1H), 7.16 (tt, $J_{H\text{-}H}$ = 7.2, 1.1 Hz, 1H), 7.13 – 7.08 (m, 2H), 6.96 (d with satellites, $J_{H\text{-}H}$ = 2.1 Hz, $J_{H\text{-}Pt}$ = 26 Hz 1H), 6.89 (ddd, $J_{H\text{-}H}$ = 7.6, 6.8, 1.6 Hz, 1H). $^{13}C\{^1H\}$ APT NMR (151 MHz, $CD_2Cl_2$): δ 164.0 (C), 158.0 (C), 157.5 (C), 153.6 (C), 152.1 ($J_{C\text{-}Pt}$ ~ 17 Hz, CH), 151.8 (C), 151.2 (C), 147.8 (CH), 139.7 (CH), 132.5 (C), 130.3 (2CH), 126.9 (CH), 125.4 (CH), 123.9 (CH), 122.8 ($J_{C\text{-}Pt}$ = 60 Hz, CH), 120.8 ($J_{C\text{-}Pt}$ = 20 Hz, CH), 119.4 (2CH), 117.4 ($J_{C\text{-}Pt}$ = 29 Hz, CH), 112.2 (C), 110.4 ($J_{C\text{-}Pt}$ = 33 Hz, CH), 108.9 ($J_{C\text{-}Pt}$ ~19 Hz, CH). Anal. Calcd for $C_{24}H_{15}NO_3Pt$: C, 51.43; H, 2.70; N, 2.50. Found: C, 51.31; H, 2.84; N, 2.52.

**[Pt(dPhOppy)(imz)] (10).** A Carius tube was charged with [$PtCl_2(NCPh)_2$] (100 mg, 0.21 mmol), dPhOppyH (79 mg, 0.23 mmol), $Na_2CO_3$ (45 mg, 0.42 mmol) and acetone (8 mL). The mixture was irradiated with violet light under vigorous stirring for 16 h. The solvent was removed under reduced pressure and the residue was suspended in 1,2-dichloroethane (5 mL) (**mixture A**). 1-Butyl-3-methylimidazolium iodide (68 mg, 0.254 mmol) and $Ag_2O$ (59 mg, 0.254 mmol) were suspended in 1,2-dichloroethane (8 mL) and the mixture was stirred at 40 °C for 2 h under an $N_2$ atmosphere protected from light. The solids were removed by filtration through Celite and **mixture A** was immediately added to the filtrate. The mixture was bubbled with $N_2$, stirred at 40 °C in the dark for 30 min, and then filtered through Celite. The complex was purified in a short silica gel chromatography column using $CH_2Cl_2$ as the eluent. The yellow fraction was collected and evaporated to dryness to give complex **10** as a yellow solid. Yield: 48 mg (34%). $^1H$ NMR (600 MHz, $CD_2Cl_2$): δ 7.82 (ddd, $J_{H\text{-}H}$ = 8.2, 7.2, 1.6 Hz, 1H), 7.80 – 7.76 (m, 2H), 7.37 – 7.29 (m, 2H), 7.22 (d, $J_{H\text{-}H}$ = 2.1 Hz, 1H), 7.15 (dd, $J_{H\text{-}H}$ = 6.9, 2.2 Hz, 2H), 7.12 – 7.04 (m, 4H), 7.02 (ddd, $J_{H\text{-}H}$ = 7.1, 5.7, 1.4 Hz, 1H), 7.00 (ddd, $J_{H\text{-}H}$ = 7.9, 6.9, 1.8 Hz, 1H), 6.89 (d with satellites, $J_{H\text{-}H}$ = 2.1 Hz, $J_{H\text{-}Pt}$ = 19 Hz, 1H), 6.67 (dd with satellites, $J_{H\text{-}H}$ = 7.6, 1.7 Hz, $J_{H\text{-}Pt}$ = 89 Hz, 1H), 6.54 (ddd, $J_{H\text{-}H}$ = 7.6, 6.9, 1.5 Hz, 1H), 4.40 (dt, $J_{H\text{-}H}$ = 13.5, 7.3 Hz, 1H), 4.20 (dt, $J_{H\text{-}H}$ = 13.4, 7.2 Hz, 1H), 3.88 (s, 3H), 1.82 – 1.72 (m, 2H), 1.30 – 1.20 (m, 2H), 0.76 (t, $J_{H\text{-}H}$ = 7.4 Hz, 2H). $^{13}C\{^1H\}$ APT NMR (151 MHz, $CD_2Cl_2$): δ 190.0 (C), 166.6 (C), 158.9 (C), 154.3 (C), 153.8 (C), 153.5 (C), 150.8 (CH), 149.3 (C), 144.5 (CH), 137.8 (CH), 137.0 (C), 130.0 (CH), 124.3 (CH), 123.7 ($J_{C\text{-}Pt}$ ~ 11 Hz, CH), 122.7 (CH), 122.0 (CH), 120.9 (2CH), 120.9 ($J_{C\text{-}Pt}$ ~ 64 Hz, CH), 120.0 ($J_{C\text{-}Pt}$ ~ 20 Hz, 2CH), 118.2 (CH), 116.3 ($J_{C\text{-}Pt}$ ~ 31 Hz, CH), 115.3 (C), 110.6 ($J_{C\text{-}Pt}$ ~ 28 Hz, CH), 109.7 ($J_{C\text{-}Pt}$ ~ 14 Hz, CH), 50.6 ($CH_2$), 37.9 ($CH_3$), 32.7 ($CH_2$), 20.1 ($CH_2$), 13.7 ($CH_3$). Anal. Calcd for $C_{31}H_{29}N_3O_2Pt$: C, 55.52; H, 4.36; N, 6.27. Found: C, 55.51; H, 4.42; N, 6.28.

**[Pt(dPhOppy)(trz)] (11).** This complex was obtained as described for **10**, from 4-butyl-3-methyl-1-phenyl-1*H*-1,2,3-triazolium iodide (87 mg, 0.254 mmol), $Ag_2O$ (59 mg, 0.254 mmol), [$PtCl_2(NCPh)_2$] (100 mg, 0.21 mmol), dPhOppyH (79 mg, 0.23 mmol) and $Na_2CO_3$ (45 mg, 0.42 mmol). After filtration through silica gel using $CH_2Cl_2$ as eluent, the yellow fraction was collected, evaporated to dryness and the residue was washed with MeOH (2 mL) to give complex **11** as a yellow solid. Yield: 70 mg, 44%. $^1H$ NMR (600 MHz, $CD_2Cl_2$): δ 8.64 – 8.50 (m, 2H), 7.83 (ddd with satellites, $J_{H\text{-}H}$ = 5.6, 1.6, 0.9 Hz, $J_{H\text{-}Pt}$ ~ 18 Hz, 1H), 7.76 – 7.68 (m, 2H), 7.39 – 7.29 (m, 5H), 7.18 (d, $J_{H\text{-}H}$ = 2.1 Hz, 1H), 7.12 – 7.03 (m, 4H), 7.02 (dd with satellites, $J_{H\text{-}H}$ = 7.7, 1.7 Hz, $J_{H\text{-}Pt}$ ~ 89 Hz, 1H), 6.96 (ddd, $J_{H\text{-}H}$ = 7.9, 6.9, 1.7 Hz, 1H), 6.88 (ddd, $J_{H\text{-}H}$ = 6.6, 5.6, 2.1 Hz, 1H), 6.86 (d with satellites, $J_{H\text{-}H}$ = 2.0 Hz, $J_{H\text{-}Pt}$ ~ 18 Hz, 1H), 6.49 (ddd, $J_{H\text{-}H}$ = 7.6, 6.9, 1.5 Hz, 1H), 4.28 (s, 3H), 3.06 (ddd, $J_{H\text{-}H}$ = 15.1, 8.7, 6.7 Hz, 1H), 2.85 (ddd, $J_{H\text{-}H}$ = 14.9, 8.7, 6.6 Hz, 1H), 1.75 – 1.61 (m, 2H), 1.32 – 1.17 (m, 2H), 0.71 (t, $J_{H\text{-}H}$ = 7.3 Hz, 3H). $^{13}C\{^1H\}$ APT NMR (151 MHz, $CD_2Cl_2$): δ 177.4 (C), 166.7 (C), 159.0 (C), 154.0 (C), 153.8 (C), 153.7 (C), 150.3 ($J_{C\text{-}Pt}$ ~ 19 Hz, CH), 149.1 (C), 147.9 ($J_{C\text{-}Pt}$ ~ 42 Hz, C), 144.2 (CH), 140.3 (C), 137.6 (CH), 136.7 (C), 130.0 (2CH), 129.4 (CH), 129.2 (2CH), 123.9 (CH), 123.8 (2CH), 123.1 ($J_{C\text{-}Pt}$ ~ 11 Hz, CH), 122.6 (CH), 120.6 ($J_{C\text{-}Pt}$ = 65 Hz, CH), 119.7 ($J_{C\text{-}Pt}$ ~ 19 Hz, CH), 118.1 (2CH), 116.2 ($J_{C\text{-}Pt}$ = 28 Hz, CH), 115.3 (C), 110.5 ($J_{C\text{-}Pt}$ ~ 25 Hz, CH), 109.6 ($J_{C\text{-}Pt}$ ~ 17 Hz,

CH), 36.9 ($CH_3$), 30.8 ($CH_2$), 25.9 ($CH_2$), 22.8 ($CH_2$), 13.8 ($CH_3$). Anal. Calcd for $C_{36}H_{32}N_4O_2Pt$: C, 57.83; H, 4.31; N, 7.49. Found: C, 57.87; H, 4.20; N, 7.28.

**[Pt(dmtppy)(γ-pic)] (13).** The synthesis of this complex has been previously reported by us using [$PtCl_2(NCPh)_2$] as precursor.[3] The following is an alternative procedure: To a solution of **12** (60 mg, 0.074 mmol) in $CH_2Cl_2$ (10 mL) was added γ-picoline (22 μL, 0.22 mmol) and the mixture was stirred for 1 h. The resulting solution was filtered through a silica pad, the yellow filtrate was concentrated (2 mL) and $Et_2O$ (15 mL) was added, whereupon a yellow solid precipitated, which was collected by filtration, washed with $Et_2O$ (3 × 3 mL) and vacuum-dried to give **13**. Yield: 24 mg, 53%. The $^1$H NMR data are in agreement with those previously reported.[3]

**[Pt(dmtppy)(CNXy)] (14).** To a solution of **12** (50 mg, 0.062 mmol) in $CH_2Cl_2$ (7 mL) was added 2,6-dimethylphenyl isocyanide (10 mg, 0.074 mmol) and the mixture was stirred for 1 h. The solvent was removed under reduced pressure and the residue was washed with MeOH (2 mL) and $Et_2O$ (2 × 2 mL) to give **14** as a red solid. Yield: 26 mg, 64%. $^1$H NMR (600 MHz, $CD_2Cl_2$): δ 8.85 (br d with satellites, $J_{H-H}$ = 5.4 Hz, $J_{H-Pt}$ ~ 18 Hz, 1H), 7.96 (td, $J_{H-H}$ = 7.2, 1.6 Hz, 1H), 7.83 (d, $J_{H-H}$ = 8.0 Hz, 1H), 7.57 (d, $J_{H-H}$ = 8.1 Hz, 2H), 7.48 (d, $J_{H-H}$ = 1.4 Hz, 1H), 7.47 (s with satellites, $J_{H-Pt}$ = 76 Hz, 1H), 7.46 (d, $J_{H-H}$ = 1.4 Hz, $J_{H-Pt}$ ~ 6 Hz, 1H), 7.34 – 7.31 (m, 1H), 7.28 (d, $J_{H-H}$ = 7.7 Hz, 2H), 7.26 – 7.22 (m, 4H), 6.83 (d, $J_{H-H}$ = 7.7 Hz, 1H), 2.60 (s, 6H), 2.41 (s, 3H), 2.25 (s, 3H). $^{13}C\{^1H\}$ APT NMR (151 MHz, $CD_2Cl_2$): δ 176.3 (C), 167.9 (C), 164.1 (C), 155.7 (C), 154.8 (C), 153.3 (CH), 145.4 (C), 143.6 (C), 140.7 ($J_{C-Pt}$ = 58 Hz, CH), 140.1 (C), 139.1 (CH), 139.0 (C), 137.0 (2C), 136.9 (C), 135.5 (2C), 129.8 (2CH), 129.2 (CH), 128.6 (2CH), 127.2 (2CH), 125.5 (CH), 124.1 (CH), 120.6 ($J_{C-Pt}$ = 66 Hz, CH), 119.9 (CH), 119.5 ($J_{C-Pt}$ = 47 Hz, CH), 118.9 ($J_{C-Pt}$ = 31 Hz, CH), 21.5 ($CH_3$), 21.2 ($CH_3$), 19.5 (2$CH_3$). Anal. Calcd for $C_{34}H_{28}N_2Pt$: C, 61.90; H, 4.28; N, 4.25. Found: C, 61.70; H, 4.43; N, 4.24.

**[Pt(dmtppy)(CO)] (15).** CO was bubbled through a solution of **12** (100 mg, 0.124 mmol) in $CH_2Cl_2$ (15 mL) for 5 min and the mixture was stirred for 1 h. The solvent was removed under reduced pressure, the residue was dissolved in the minimum amount of $CH_2Cl_2$ and the solution was passed through silica gel in a short chromatography column. The yellow fraction was collected and concentrated (2 mL) and $Et_2O$ was added (10 mL). The orange precipitate was collected by filtration and washed with $Et_2O$ (2 × 2 mL) and vacuum-dried to give **15**. Yield: 48 mg, 70%. $^1$H NMR (600 MHz, $CD_2Cl_2$): δ 8.55 (br s, 1H), 7.91 (t, $J_{H-H}$ = 7.8 Hz, 1H), 7.71 (d, $J_{H-H}$ = 8.0 Hz, 1H), 7.51 (d, $J_{H-H}$ = 8.0 Hz, 1H), 7.38 (s, 2H), 7.28 (d, $J_{H-H}$ = 7.9 Hz, 1H), 7.23 (s with satellites, $J_{H-Pt}$ ~ 80 Hz, 1H), 7.21-7.16 (m, 2H), 6.86 (ddd, $J_{H-H}$ = 7.6, 1.7, 0.9 Hz, 1H), 2.41 (s, 3H), 2.28 (s, 3H). $^{13}C\{^1H\}$ APT NMR (151 MHz, $CD_2Cl_2$): δ 189.6 (C), 175.4 (C), 166.9 (C), 155.9 (C), 154.0 (CH), 153.9 (C), 146.6 ($J_{C-Pt}$ ~ 47 Hz, C), 142.1 (C), 140.7 (CH), 140.6 (C), 139.7 (CH), 139.5 (C), 137.9 (C), 137.5 (C), 129.8 (2CH), 127.2 (2CH), 126.5 (CH), 124.8 (CH), 121.2 ($J_{C-Pt}$ ~ 67 Hz, CH), 120.1 (CH), 120.1 (CH), 119.1 ($J_{C-Pt}$ ~ 30 Hz, CH), 21.6 ($CH_3$), 21.2 ($CH_3$). Anal. Calcd for $C_{26}H_{19}NPtO$: C, 56.11; H, 3.44; N, 2.52. Found: C, 56.11; H, 3.78; N, 2.60.

**[Pt(dmtppy)(imz)] (16).** 1-Butyl-3-methylimidazolium iodide (30 mg, 0.122 mmol) and $Ag_2O$ (28 mg, 0.122 mmol) were suspended in 1,2-dichloroethane (5 mL) and the mixture was stirred at 40 °C for 2 h under an $N_2$ atmosphere protected from light. The solids were removed by filtration through Celite and **12** (60 mg, 0.074 mmol) was immediately added to the filtrate. The mixture was stirred at 40 °C in the dark for 1 h and then filtered through Celite. The filtrate was passed through a short silica gel chromatography column using $CH_2Cl_2$ as the eluent. The yellow fraction was collected and evaporated to dryness to give **16** as a yellow solid. Yield: 38 mg (76%). $^1$H NMR (600 MHz, $CD_2Cl_2$): δ 8.21 (ddd with satellites, $J_{H-H}$ = 5.4, 1.7, 0.8 Hz, $J_{H-Pt}$ ~ 15 Hz, 1H), 7.90 (ddd, $J_{H-H}$ = 8.0, 7.3, 1.7 Hz, 1H), 7.85 (ddd, $J_{H-H}$ = 8.0, 1.2, 0.7 Hz,

1H), 7.61 – 7.59 (m, 2H), 7.55 (d, $J_{H-H}$ = 1.4 Hz, 1H), 7.49 (d with satellites, $J_{H-H}$ = 1.3 Hz, $J_{H-Pt}$ ~ 5 Hz, 1H), 7.28 – 7.25 (m, 3H), 7.10 (dd, $J_{H-H}$ = 10.8, 2.0 Hz, 2H), 7.07 (ddd, $J_{H-H}$ = 7.3, 5.4, 1.4 Hz, 1H), 6.76 (ddd, $J_{H-H}$ = 7.6, 1.9, 0.8 Hz, 1H), 6.50 (dd with satellites, $J_{H-H}$ = 1.3, 0.6 Hz, $J_{H-Pt}$ = 74 Hz, 1H), 4.39 – 4.34 (m, 1H), 4.23 – 4.18 (m, 1H), 3.86 (s, 3H), 2.40 (s, 3H), 2.15 (s, 3H), 1.85 – 1.78 (m, 2H), 1.31 – 1.23 (m, 2H), 0.80 (t, $J_{H-H}$ = 7.4 Hz, 3H). $^{13}C\{^1H\}$ APT NMR (151 MHz, $CD_2Cl_2$): δ 194.3 ($J_{C-Pt}$ ~ 880 Hz, C), 180.2 ($J_{C-Pt}$ = 760 Hz, C), 169.2 ($J_{C-Pt}$ = 78 Hz, C), 155.9 (C), 154.7 ($J_{C-Pt}$ = 144 Hz, C), 152.4 (CH), 146.0 ($J_{C-Pt}$ = 1180 Hz, C), 143.7 ($J_{C-Pt}$ = 42 Hz, C), 141 (C), 139.7 ($J_{C-Pt}$ = 62 Hz, CH), 138.0 (CH), 136.7 (C), 136.4 (C), 135.8 ($J_{C-Pt}$ = 72 Hz, C), 129.7 (2CH), 127.1 (2CH), 124.2 (CH), 123.1 (CH), 121.7 ($J_{C-Pt}$ ~ 20 Hz, CH), 120.5 ($J_{C-Pt}$ ~ 20 Hz, CH), 120.2 ($J_{C-Pt}$ = 68 Hz, CH), 119.6 ($J_{C-Pt}$ ~ 17 Hz, CH), 119.0 ($J_{C-Pt}$ = 40 Hz, CH), 118.8 ($J_{C-Pt}$ ~ 26 Hz, CH), 50.5 ($J_{C-Pt}$ = 29 Hz, $CH_2$), 37.8 ($J_{C-Pt}$ = 36 Hz, $CH_3$), 33.1 ($CH_2$), 21.7 ($CH_3$), 21.2 ($CH_3$), 20.1 ($CH_2$), 13.8 ($CH_3$). Anal. Calcd for $C_{33}H_{33}N_3Pt$: C, 59.45; H, 4.99; N, 6.30. Found: C, 59.50; H, 5.09; N, 6.38.

**[Pt(dmtppy)(trz)]** (**17**). This complex was obtained as a yellow solid as described for **16**, from 4-butyl-3-methyl-1-phenyl-1*H*-1,2,3-triazolium iodide (50 mg, 0.146 mmol), $Ag_2O$ (34 mg, 0.146 mmol) and **12** (100 mg, 0.124 mmol). Yield: 61 mg, 66%. $^1H$ NMR (600 MHz, $CD_2Cl_2$): δ 8.62 (br d, $J_{H-H}$ = 8.5 Hz, 2H), 8.05 (ddd with satellites, $J_{H-H}$ = 5.5, 1.5, 0.8 Hz, $J_{H-Pt}$ ~ 15 Hz, 1H), 7.84 – 7.77 (m, 2H), 7.60 (dd, $J_{H-H}$ = 8.1, 1.6 Hz, 2H), 7.54 (t, $J_{H-H}$ = 1.4 Hz, 1H), 7.49 (dd, $J_{H-H}$ = 2.3, 1.3 Hz, 1H), 7.40 – 7.31 (m, 3H), 7.31 – 7.24 (m, 3H), 6.91 (ddd, $J_{H-H}$ = 6.7, 5.4, 2.0 Hz, 1H), 6.74 (br d, $J_{H-H}$ = 7.5 Hz, 1H), 6.64 (s with satellites, $J_{H-Pt}$ = 73 Hz, 1H, CH), 4.25 (br s, 1.5H), 4.24 (br s, 1.5H), 3.08 (ddd, $J_{H-H}$ = 15.1, 8.6, 6.9 Hz, 1H), 2.87 (ddd, $J_{H-H}$ = 14.7, 8.4, 6.4 Hz, 1H), 2.40 (s, 3H), 2.11 (s, 3H), 1.80 – 1.67 (m, 2H), 1.28 (h, $J_{H-H}$ = 7.4 Hz, 2H), 0.75 (t, $J_{H-H}$ = 7.4 Hz, 3H). $^{13}C\{^1H\}$ APT NMR (151 MHz, $CD_2Cl_2$): δ 180.4 ($J_{C-Pt}$ = 770 Hz, C), 180.3 ($J_{C-Pt}$ = 795 Hz, C), 169.2 ($J_{C-Pt}$ = 79 Hz, C), 156.0 ($J_{C-Pt}$ = 120 Hz, C), 154.8 ($J_{C-Pt}$ = 148 Hz, C), 151.8 (CH), 148.4 ($J_{C-Pt}$ = 47 Hz, C), 146.5 ($J_{C-Pt}$ = 1200 Hz, C), 143.3 ($J_{C-Pt}$ = 42 Hz, C), 141.2 (C), 140.6 (C), 139.4 ($J_{C-Pt}$ = 59 Hz, CH), 137.6 (CH), 136.4 (C), 136.3 (C), 135.6 ($J_{C-Pt}$ = 71 Hz, C), 129.7 (2CH), 129.3 (CH), 129.2 (2CH), 127.1 (2CH), 123.9 (CH), 123.5 (2CH), 122.6 (CH), 120.1 ($J_{C-Pt}$ = 68 Hz, CH), 119.4 ($J_{C-Pt}$ = 19 Hz, CH), 118.8 ($J_{C-Pt}$ = 44 Hz, CH), 118.7 ($J_{C-Pt}$ = 30 Hz, CH), 36.7 ($CH_3$), 31.3 ($CH_2$), 26.1 ($CH_2$), 22.7 ($CH_2$), 21.7 ($CH_3$), 21.2 ($CH_3$), 13.9 ($CH_3$). Anal. Calcd for $C_{38}H_{36}N_4Pt$: C, 61.36; H, 4.88; N, 7.53. Found: C, 61.43; H, 5.08; N, 7.66.

## 1.4. NMR spectra of new compounds

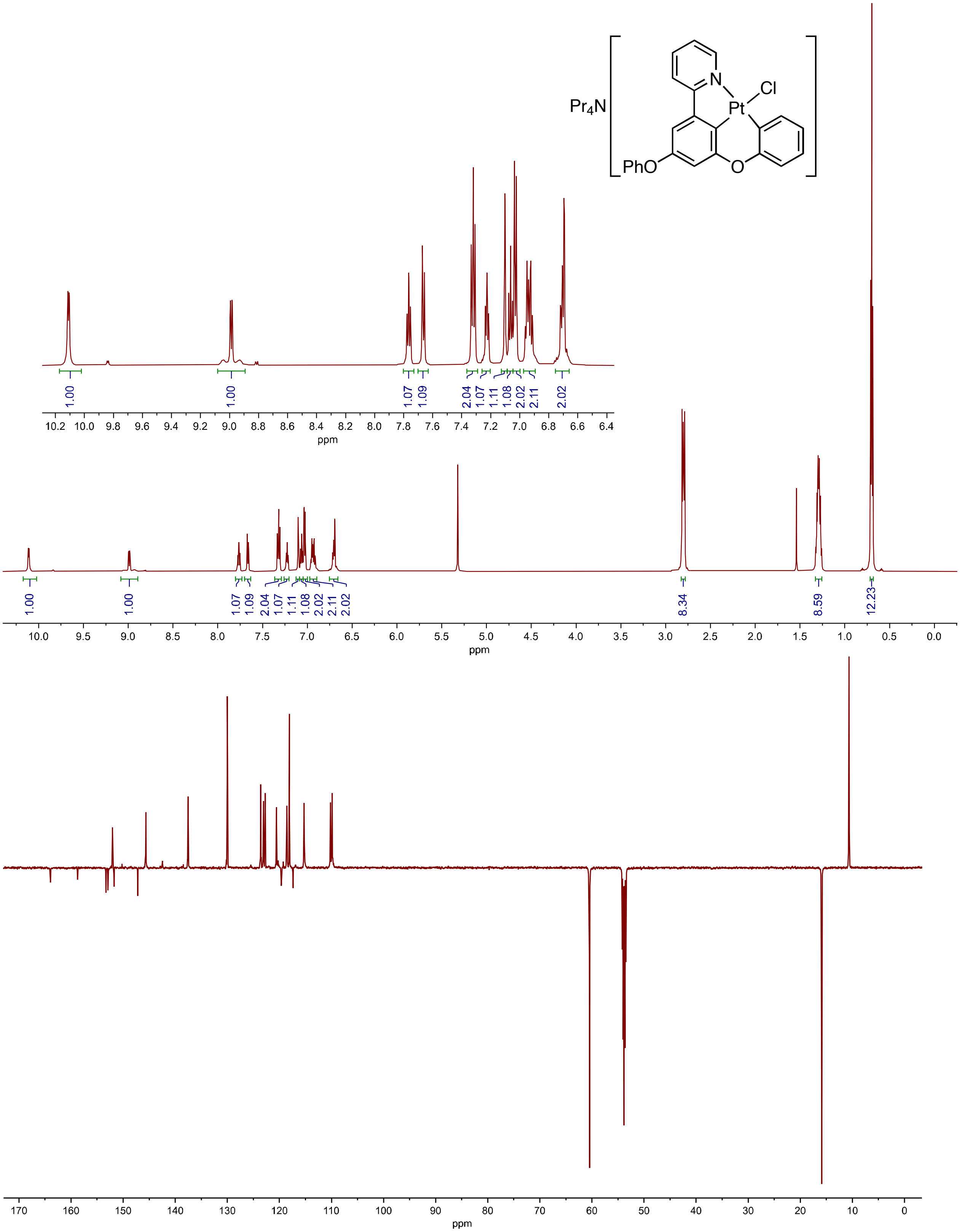


**Figure S1.** $^{1}H$ (top) and $^{13}C\{^{1}H\}$ APT (bottom) NMR spectra of complex $(Pr_4N)[PtCl(dPhOppy)]$ (**2**) ($CD_2Cl_2$, 600 and 151 MHz, respectively).

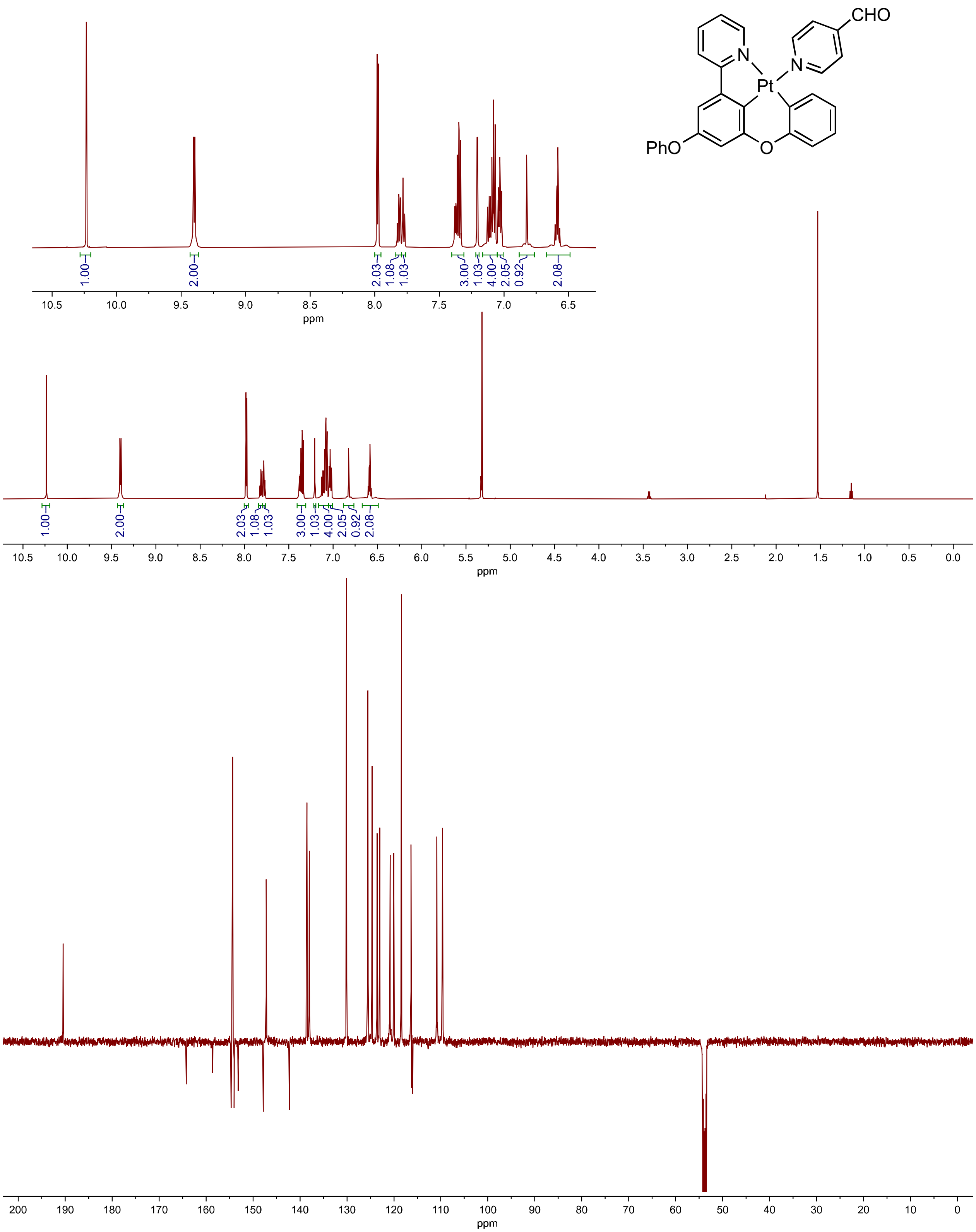


**Figure S2.** $^{1}H$ (top) and $^{13}C\{^{1}H\}$ APT (bottom) NMR spectra of complex [Pt(dPhOppy)(py-CHO-4)] (**4**) ($CD_2Cl_2$, 600 and 151 MHz, respectively).

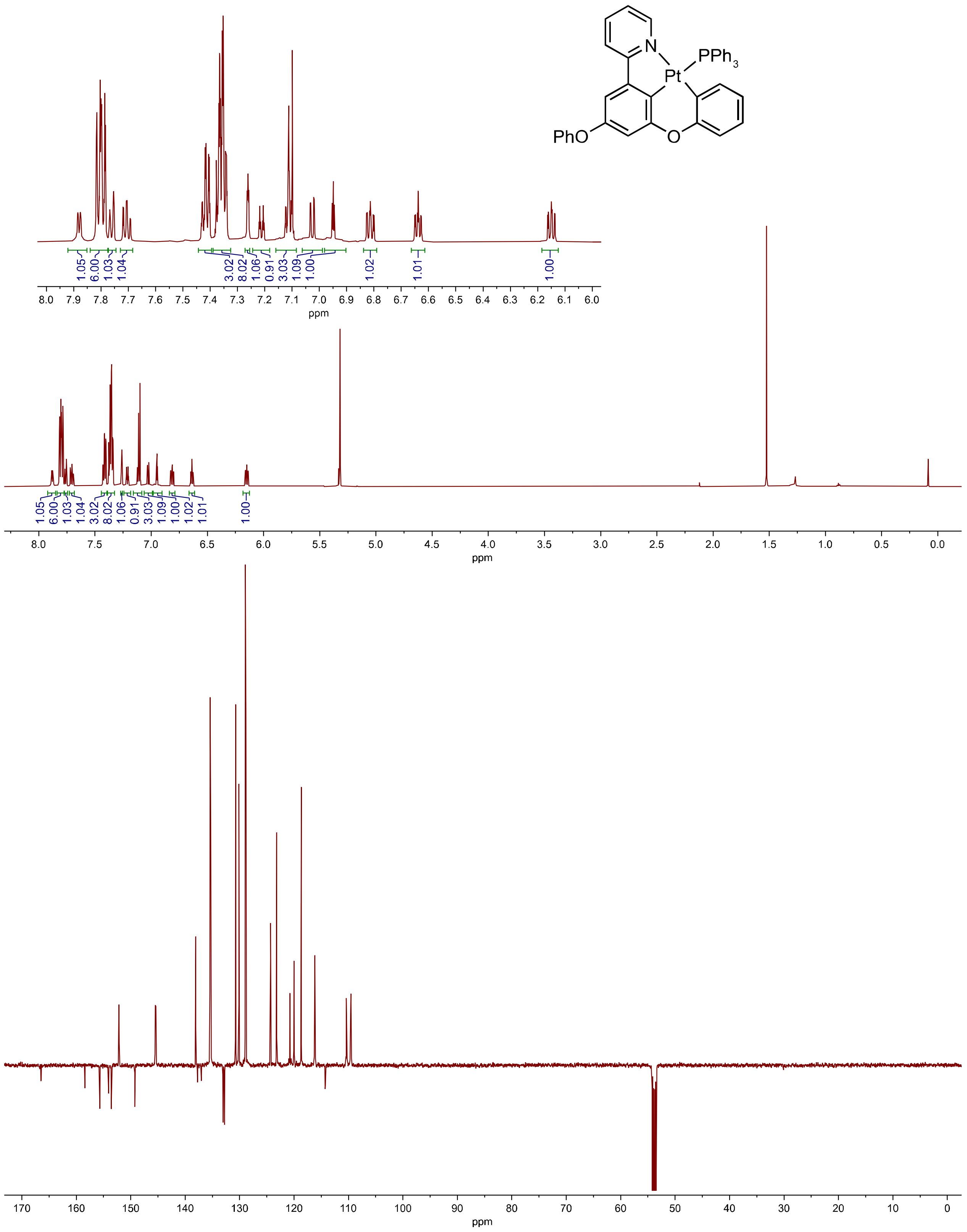


**Figure S3.** $^{1}$H (top) and $^{13}$C{$^{1}$H} APT (bottom) NMR spectra of complex [Pt(dPhOppy)(PPh$_3$)] (**5**) ($CD_2Cl_2$, 600 and 151 MHz, respectively).

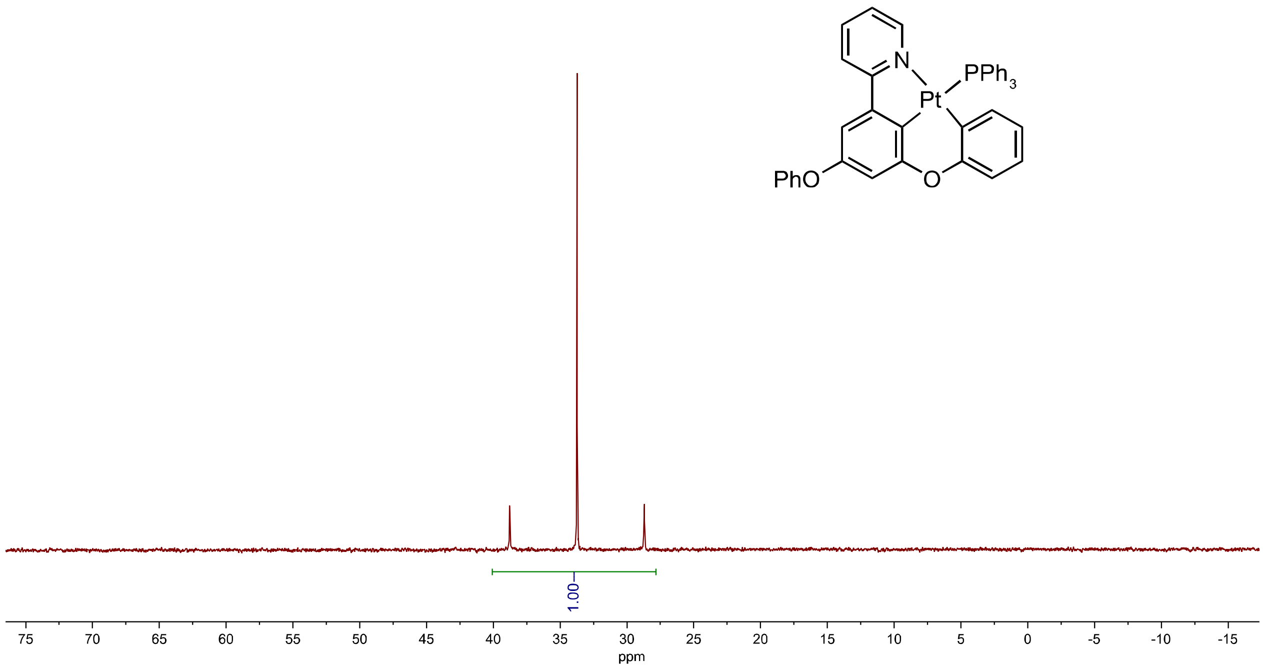


**Figure S4.** $^{31}P\{^1H\}$ NMR spectrum of complex [Pt(dPhOppy)(PPh$_3$)] (**5**) ($CD_2Cl_2$, 243 MHz).

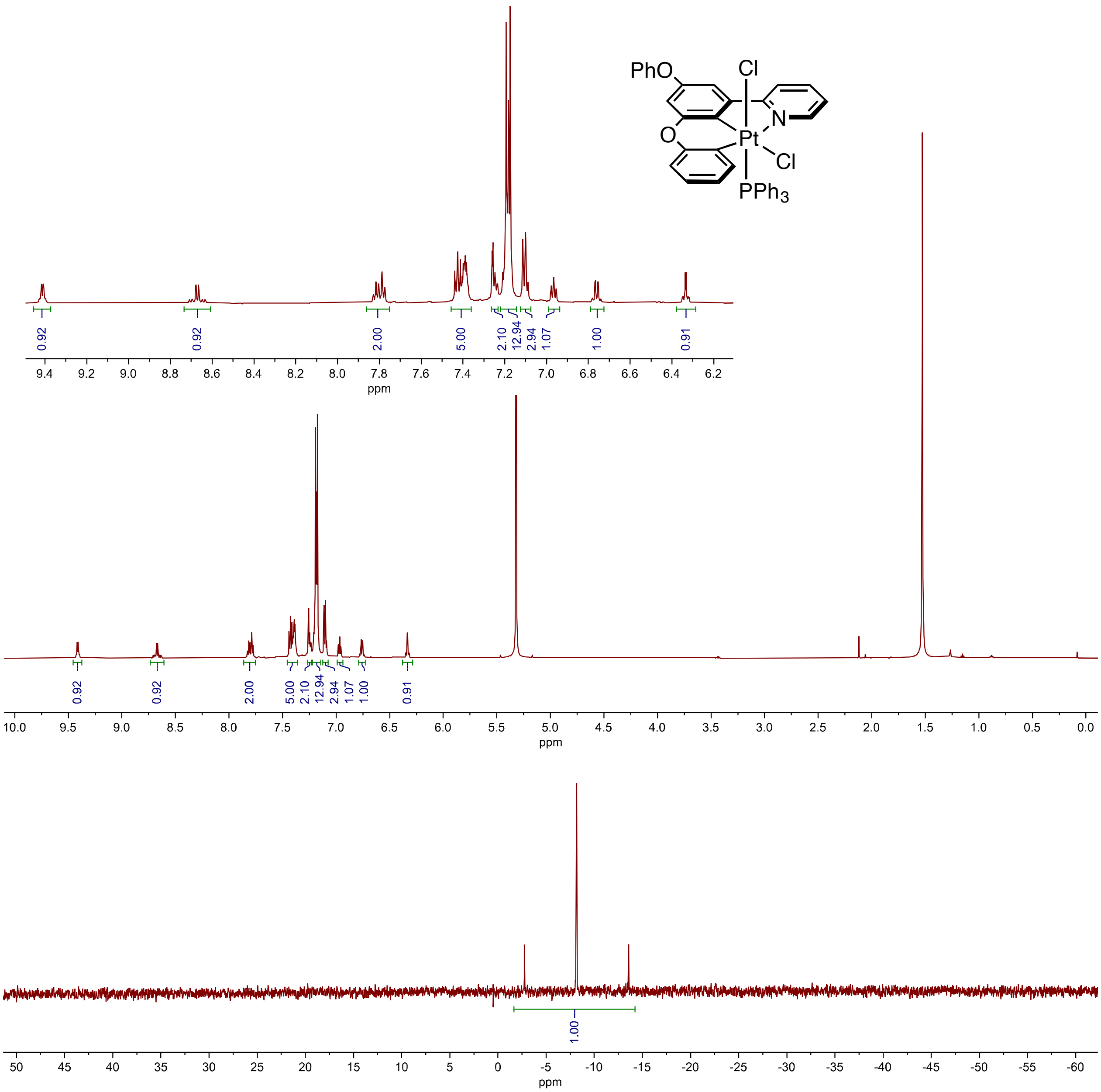


**Figure S5.** $^{1}H$ (top) and $^{31}P\{^{1}H\}$ (bottom) NMR spectra of complex [$PtCl_2$(dPhOppy)($PPh_3$)] (**6**) (600 and 243 MHz, respectively).

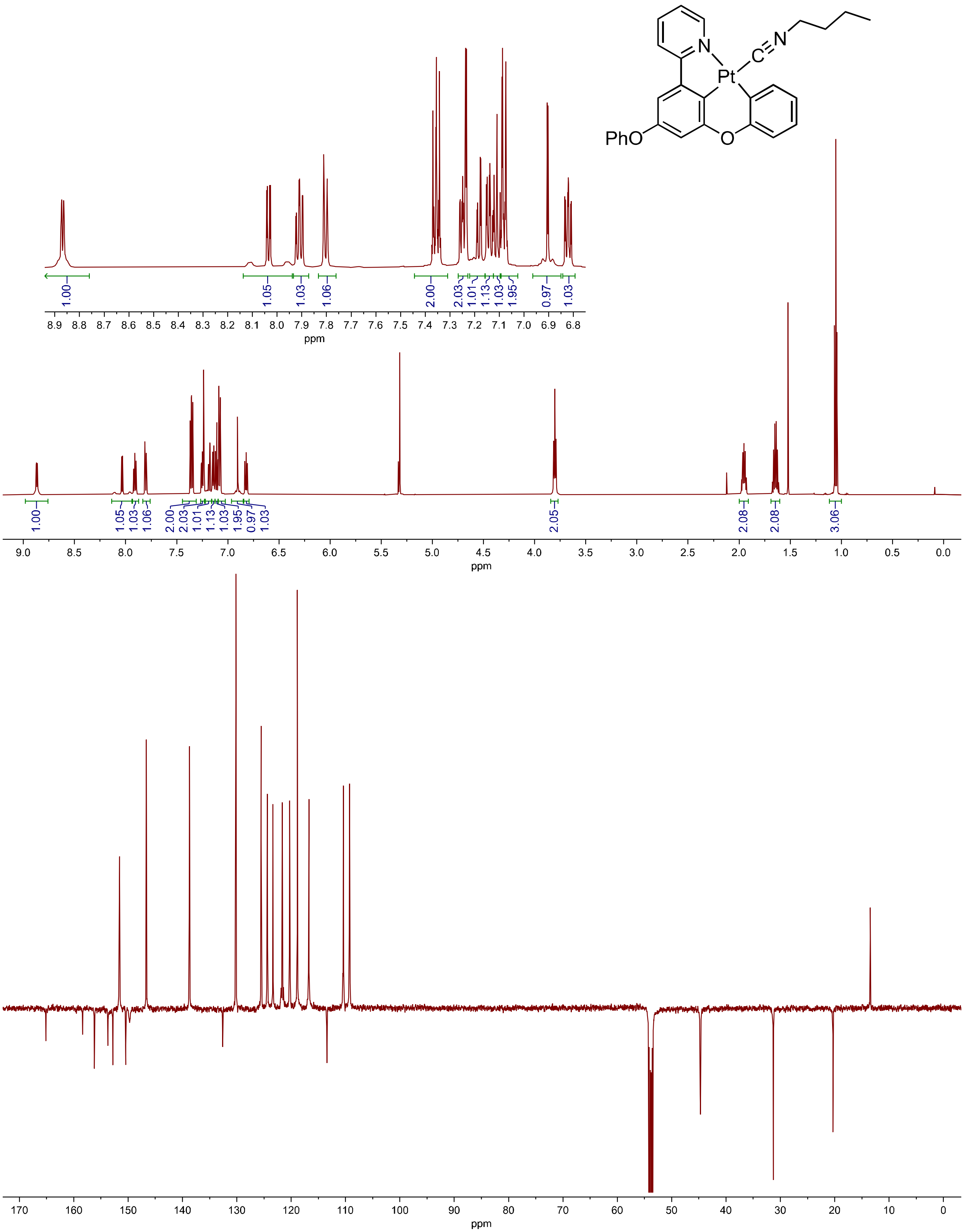


**Figure S6.** $^{1}H$ (top) and $^{13}C\{^{1}H\}$ APT (bottom) NMR spectra of complex [Pt(dPhOppy)(CNBu)] (**7**) ($CD_2Cl_2$, 600 and 151 MHz, respectively).

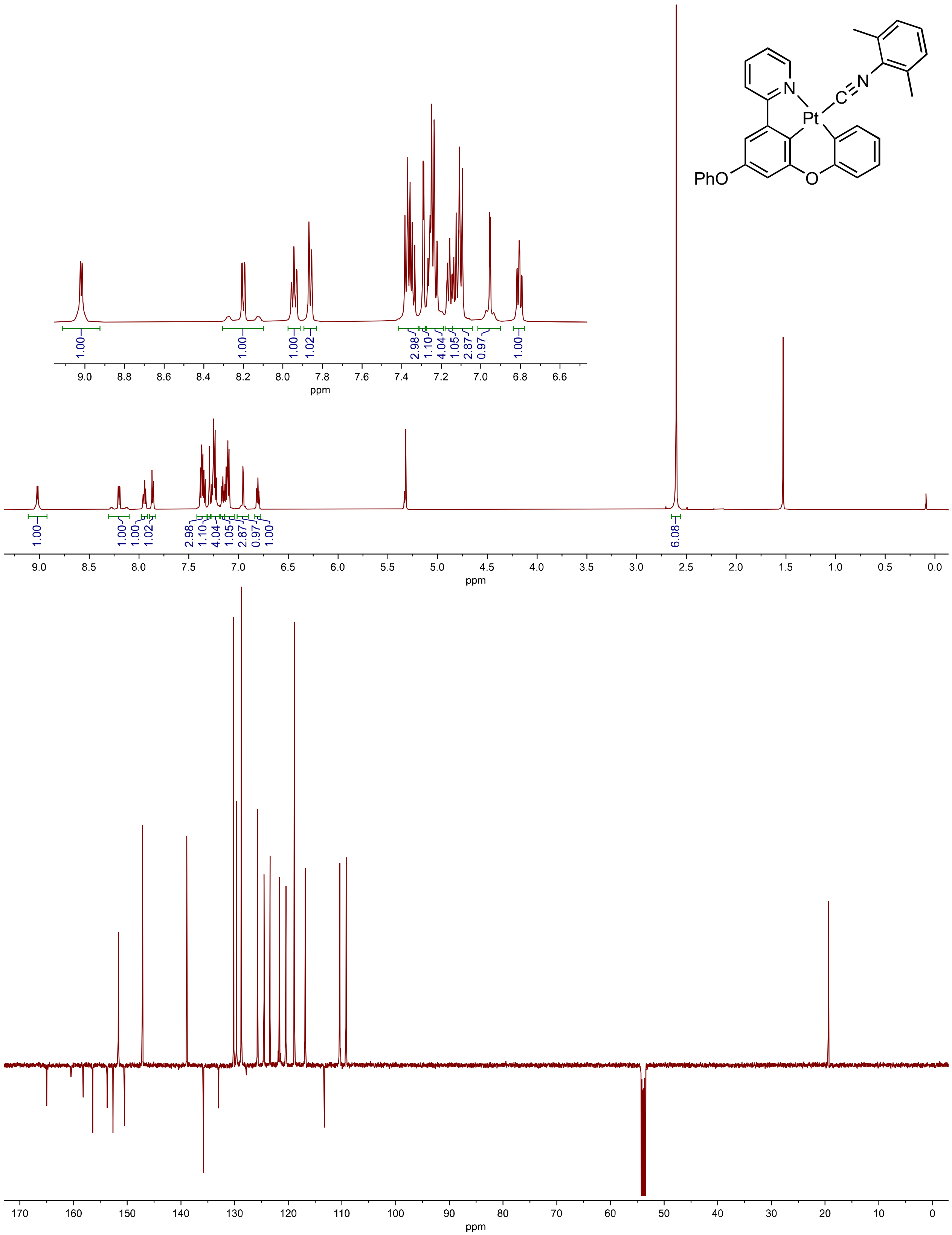


**Figure S7.** $^{1}H$ (top) and $^{13}C\{^{1}H\}$ APT (bottom) NMR spectra of complex [Pt(dPhOppy)(CNXy)] (**8**) ($CD_2Cl_2$, 600 and 151 MHz, respectively).

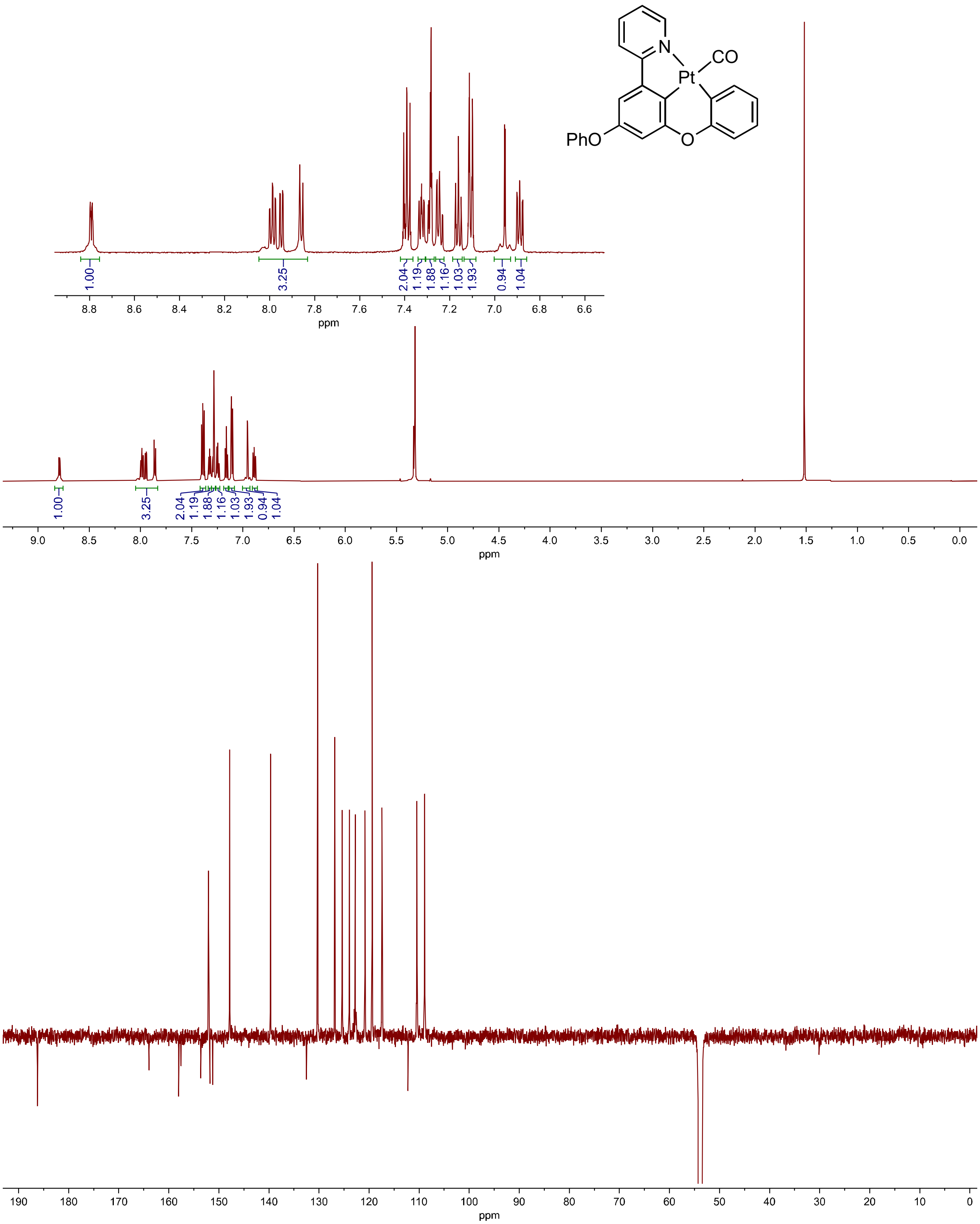


**Figure S8.** $^{1}H$ (top) and $^{13}C\{^{1}H\}$ APT (bottom) NMR spectra of complex [Pt(dPhOppy)(CO)] (**9**) ($CD_2Cl_2$, 600 and 151 MHz, respectively).

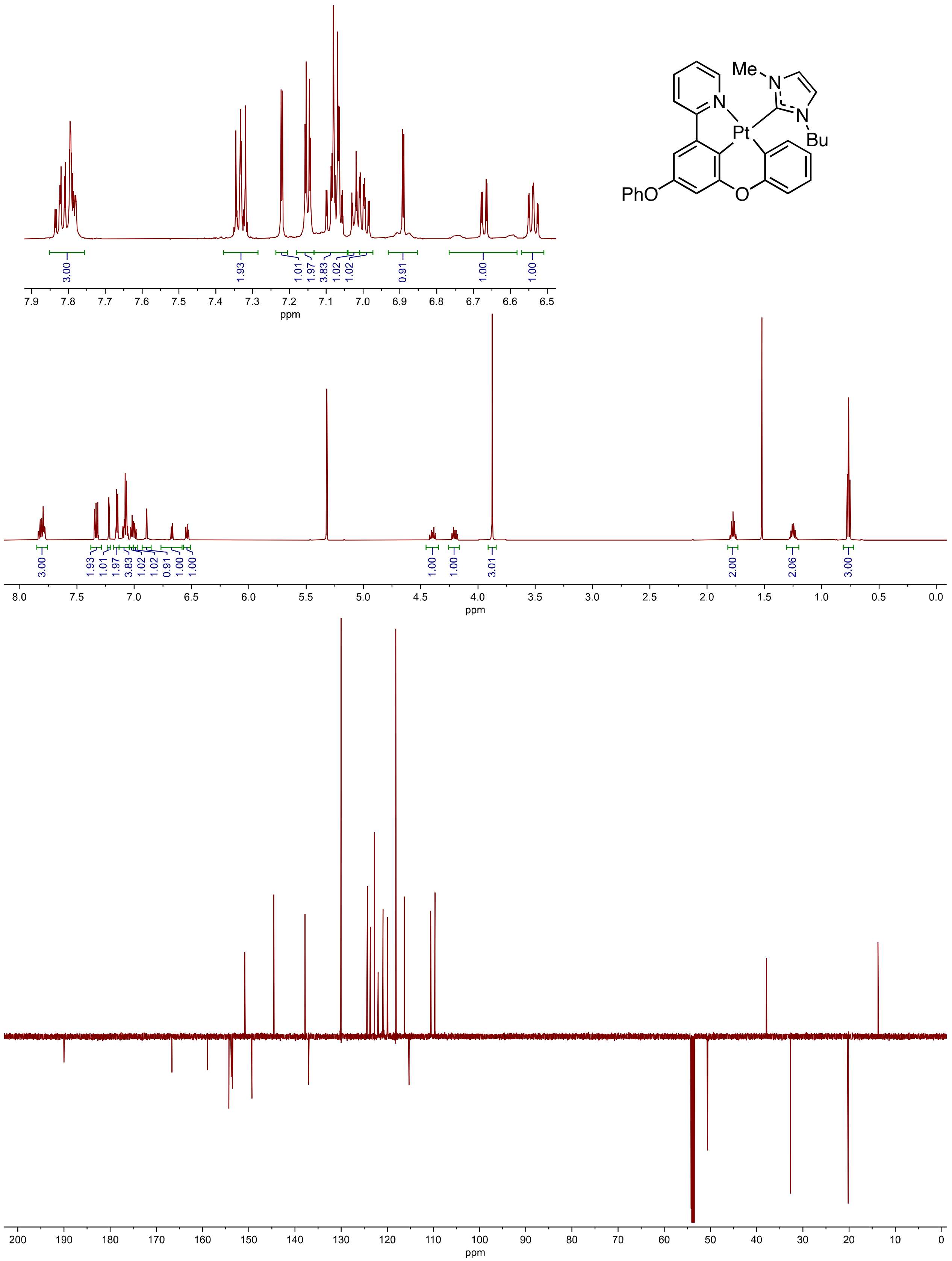


**Figure S9.** $^{1}H$ (top) and $^{13}C\{^{1}H\}$ APT (bottom) NMR spectra of complex [PtCl(dPhOppy)(imz)] (**10**) ($CD_2Cl_2$, 600 and 151 MHz, respectively).

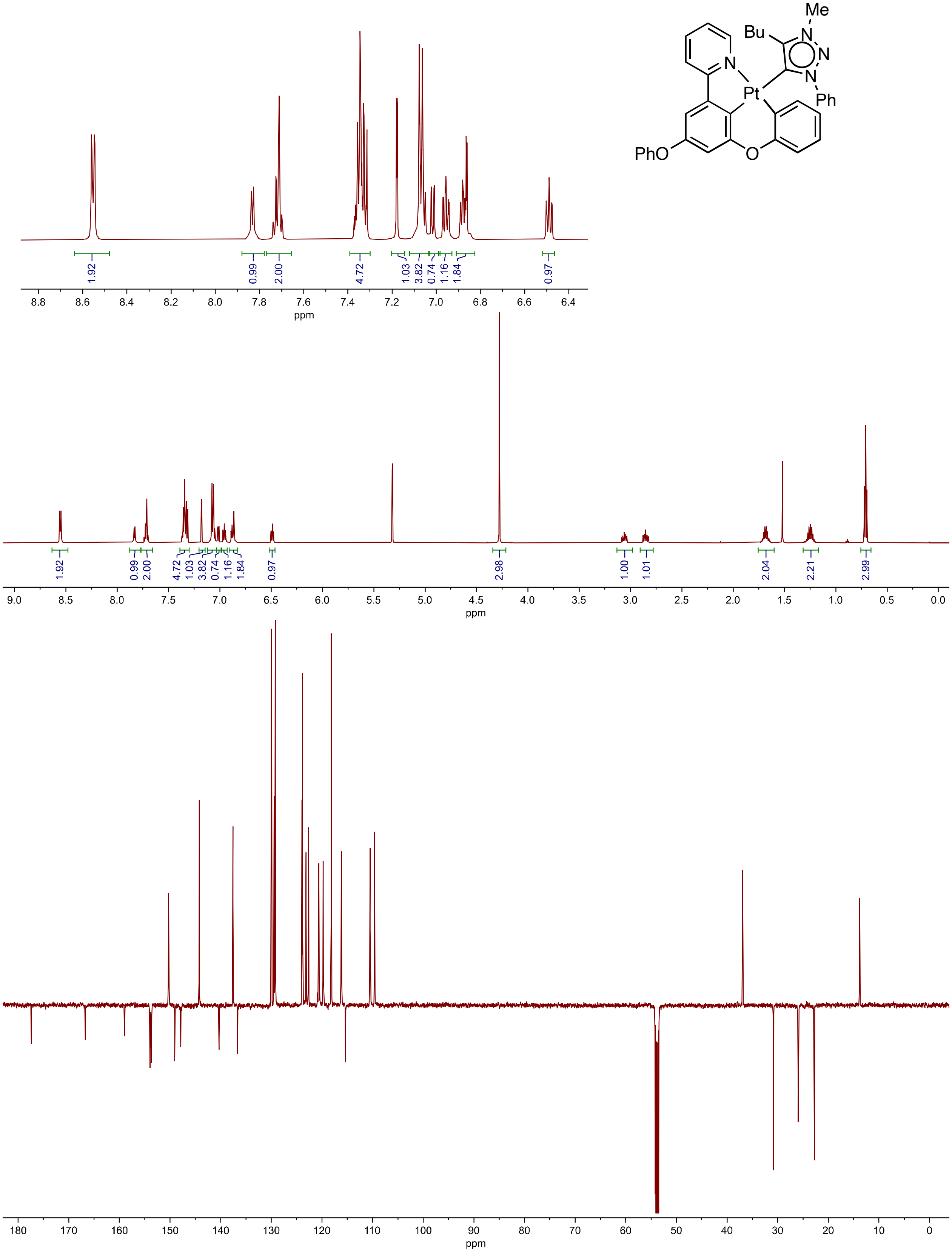


**Figure S10.** $^{1}H$ (top) and $^{13}C\{^{1}H\}$ APT (bottom) NMR spectra of complex [Pt(dPhOppy)(trz)] (**11**) ($CD_2Cl_2$, 600 and 151 MHz, respectively).

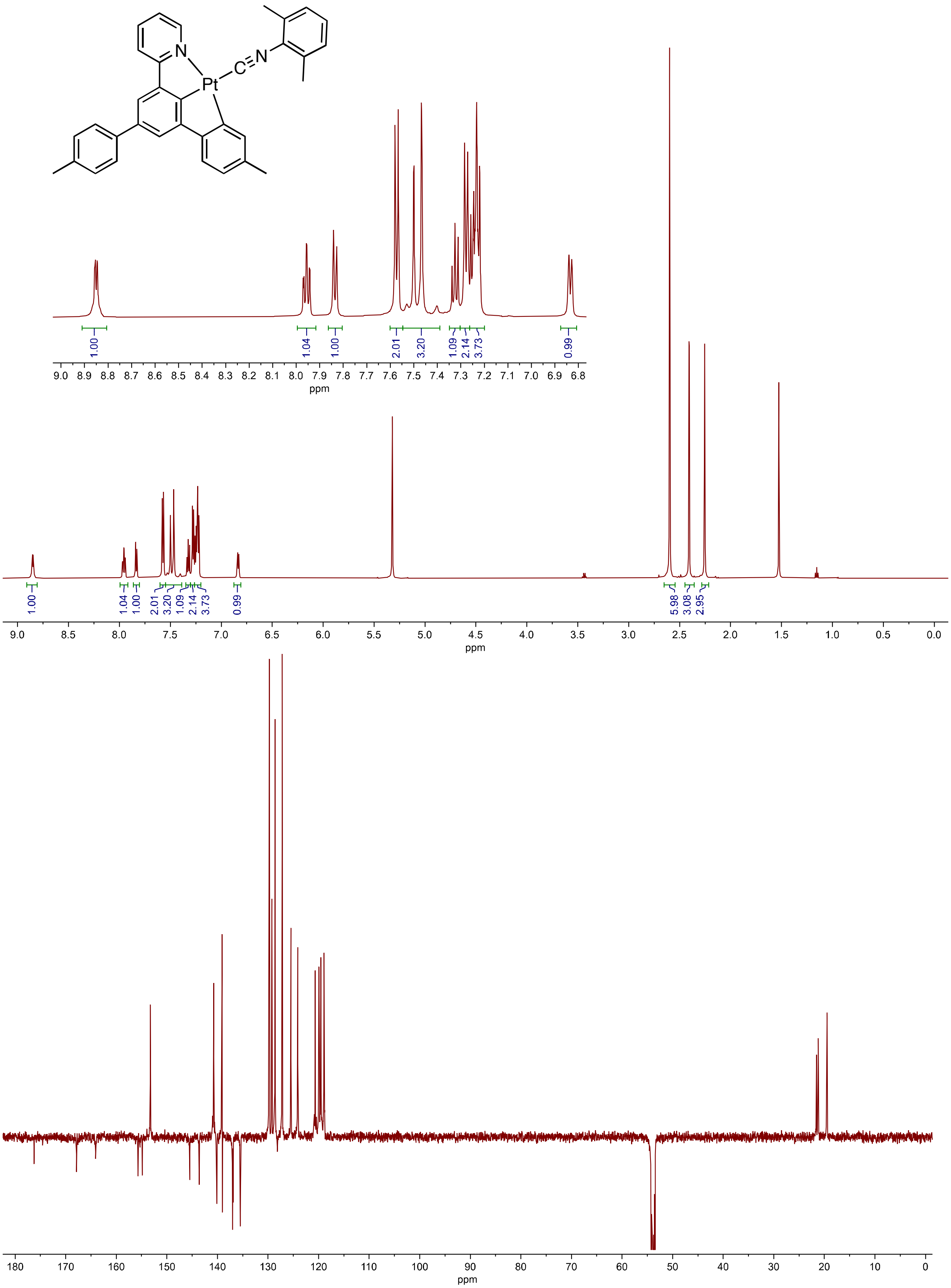


**Figure S11.** $^{1}H$ (top) and $^{13}C\{^{1}H\}$ APT (bottom) NMR spectra of complex [Pt(dmtppy)(CNXy)] (**14**) ($CD_2Cl_2$, 600 and 151 MHz, respectively).

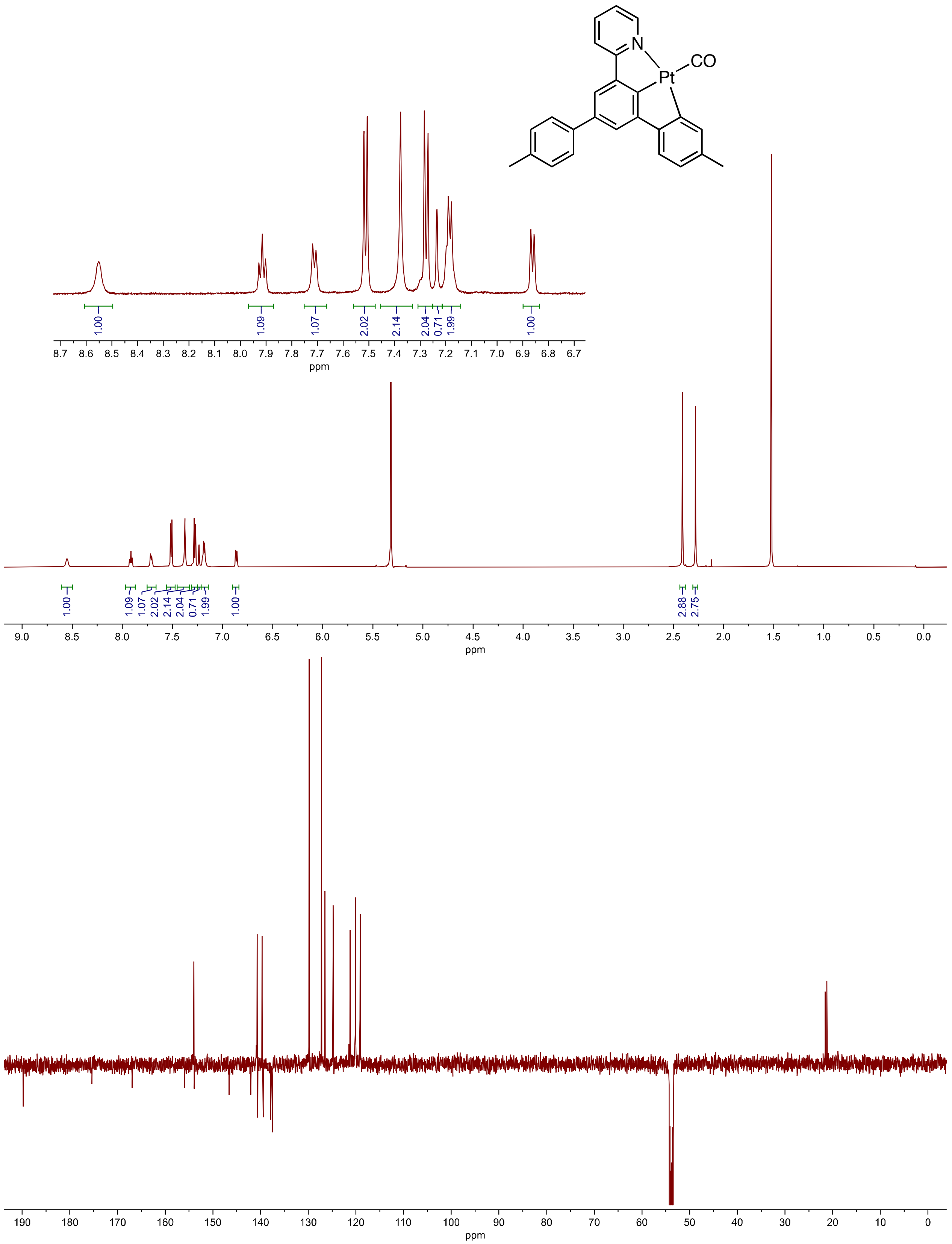


**Figure S12.** $^{1}H$ (top) and $^{13}C\{^{1}H\}$ APT (bottom) NMR spectra of complex [Pt(dmtppy)(CO)] (**15**) ($CD_2Cl_2$, 600 and 151 MHz, respectively).

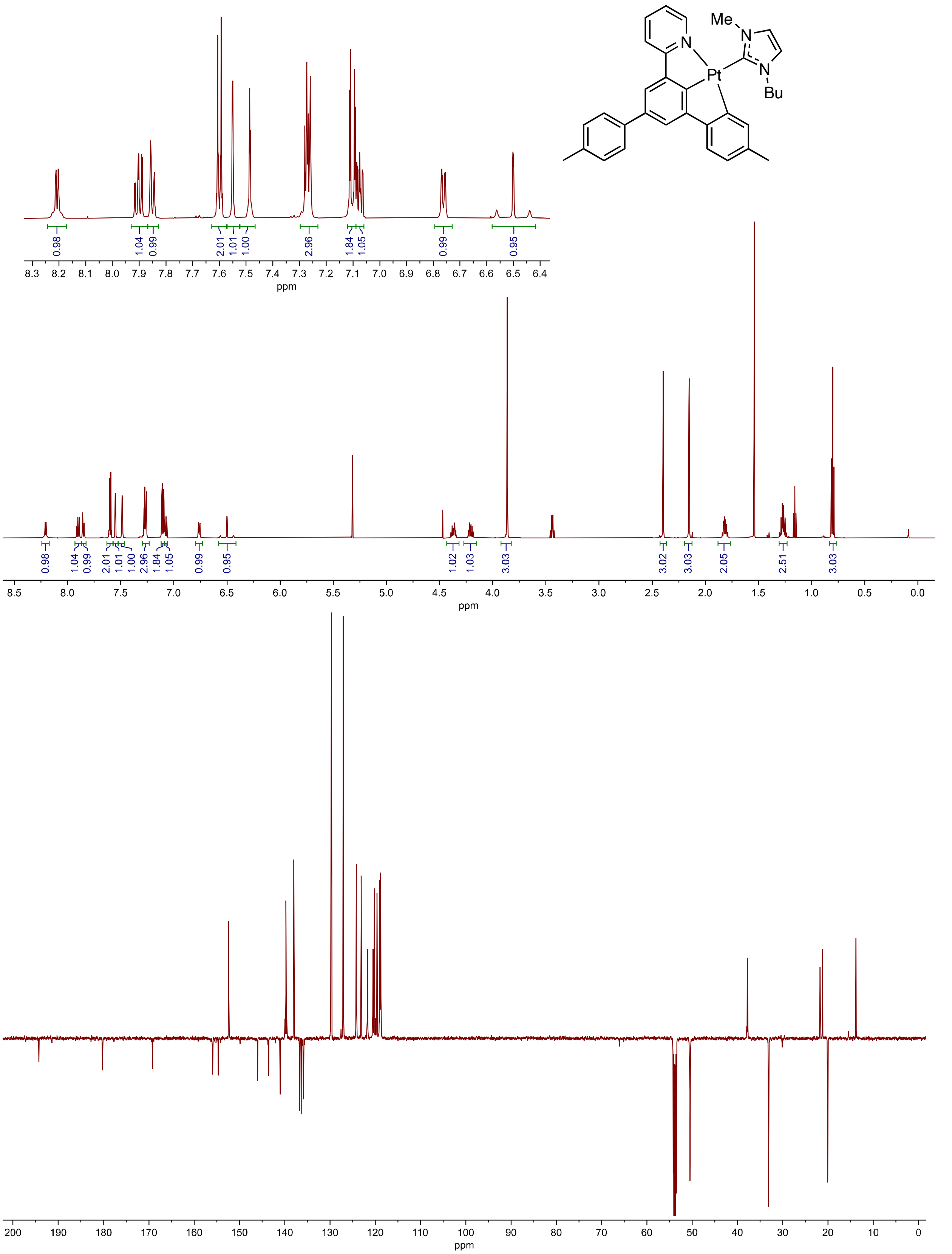


**Figure S13.** $^{1}H$ (top) and $^{13}C\{^{1}H\}$ APT (bottom) NMR spectra of complex [Pt(dmtppy)(imz)] (**16**) ($CD_2Cl_2$, 600 and 151 MHz, respectively).

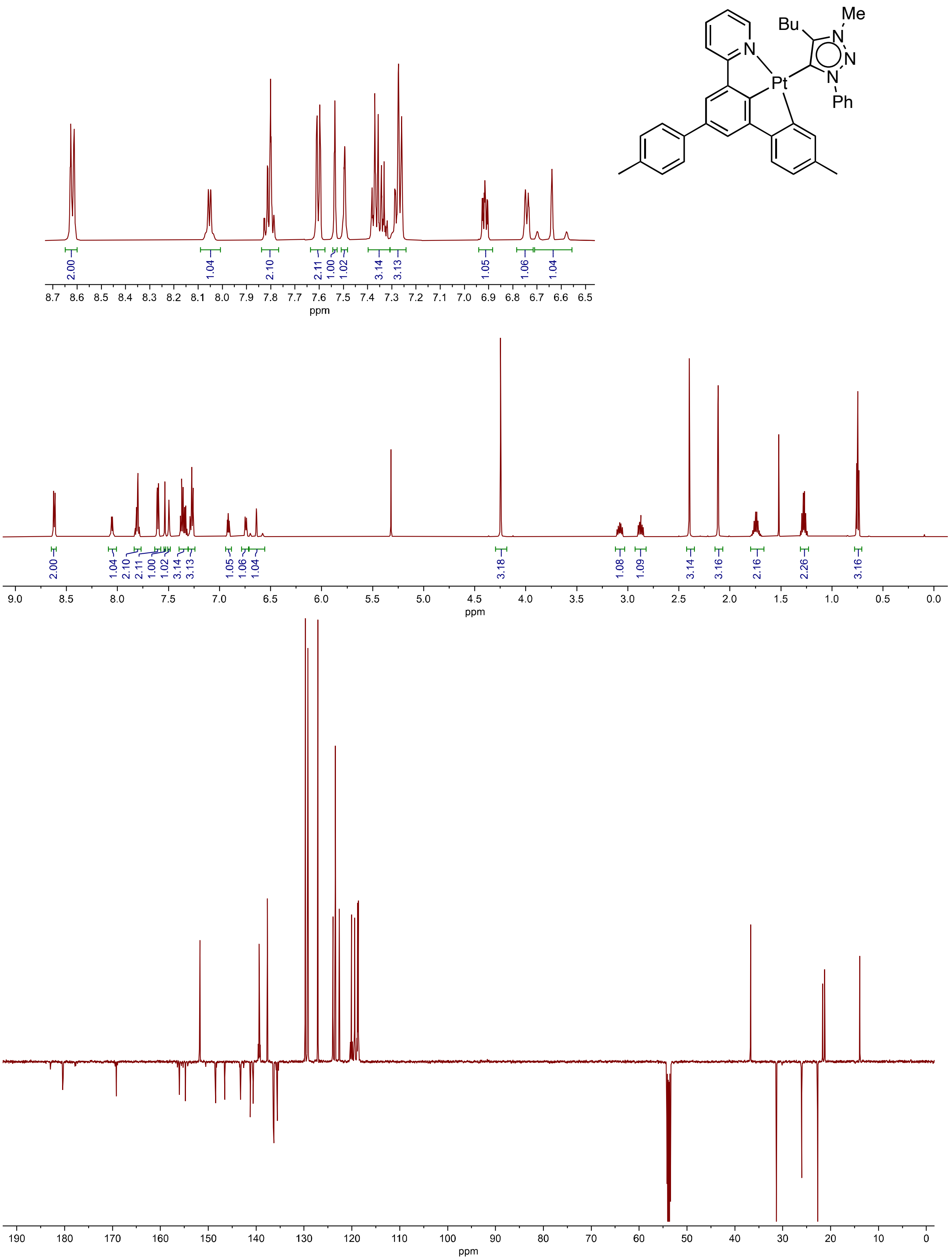


**Figure S14.** $^{1}$H (top) and $^{13}$C{$^{1}$H} APT (bottom) NMR spectra of complex [Pt(dmtppy)(trz)] (**17**) ($CD_2Cl_2$, 600 and 151 MHz, respectively).

## 1.5. Ligand exchange equilibrium of 3 in $CD_3CN$

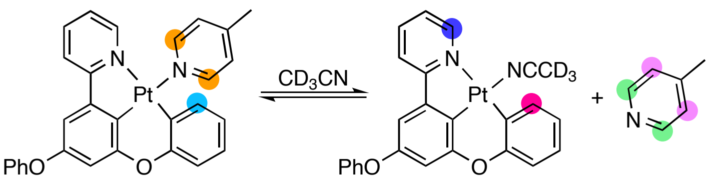


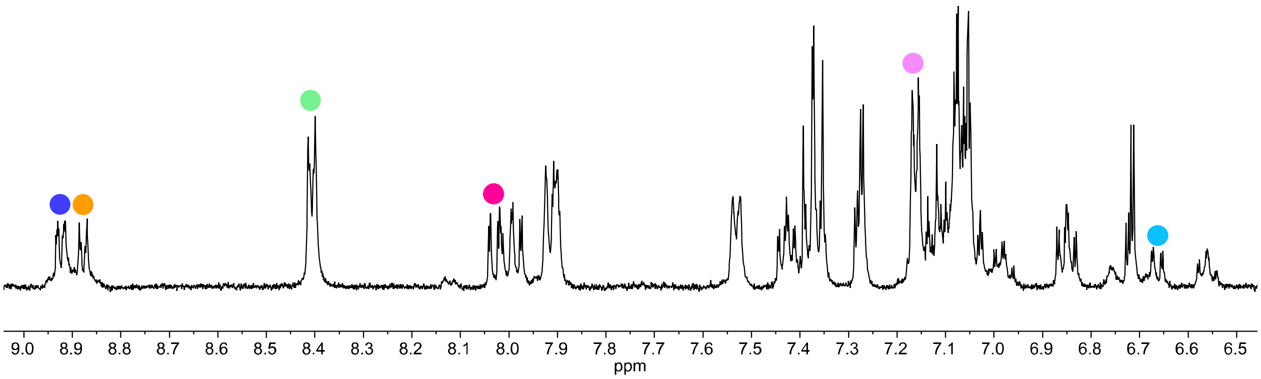


**Figure S15.** $^1H$ NMR spectrum (aromatic region) of complex **3** ($CD_3CN$, 400 MHz).

## 1.6. X-ray structure determinations

Single crystals suitable for X-ray diffraction were grown by slow liquid-liquid diffusion from $CH_2Cl_2$/*n*-pentane (**4**), $CH_2Cl_2$/acetone/$Et_2O$ (**9**), or $CH_2Cl_2/Et_2O$ (**6**, **7**, **11**, **14** and **15**). Diffraction data were collected on a Bruker D8 QUEST diffractometer with monochromated Mo-*K*α radiation performing $\varphi$ and $\omega$ scans. The structures were solved by direct methods and refined anisotropically on $F^2$ using the program SHELXL-2018 (G. M. Sheldrick, University of Göttingen).[5,6] Numerical details are presented in Tables S1 and S2. Methyl hydrogens were included as part of rigid idealized methyl groups allowed to rotate but not tip; other hydrogens were included using a riding model. *Special features of refinement:* The structure of **6** contains poorly-resolved regions of residual electron density, which could not be adequately modelled and therefore was "removed" using the program SQUEEZE, which is part of the PLATON system; the void volume per cell was 219 Å$^3$, with a void electron count per cell of 57; this additional solvent was not taken into account when calculating derived parameters such as the formula weight, because its nature was uncertain.

**Table S1.** Crystallographic data for **4**, **6**, **7** and **9**.

| | **4** | **6** | **7** | **9** |
|---|---|---|---|---|
| formula | $C_{29}H_{20}N_2O_3Pt$ | $C_{41}H_{30}Cl_2NO_2PPt$ | $C_{28}H_{24}ClN_2O_2Pt$ | $C_{24}H_{15}NO_3Pt$ |
| fw | 639.56 | 865.62 | 615.58 | 560.46 |
| $T$ (K) | 100(2) | 100(2) | 100(2) | 100(2) |
| λ | 0.71073 | 0.71073 | 0.71073 | 0.71073 |
| cryst syst | triclinic | triclinic | triclinic | monoclinic |
| space group | P-1 | P-1 | P-1 | $P2_1/n$ |
| $a$ (Å) | 10.5590(12) | 10.2888(15) | 9.0416(8) | 6.8120(16) |
| $b$ (Å) | 10.6453(12) | 10.6189(14) | 11.2066(10) | 25.400(6) |
| $c$ (Å) | 10.8890(12) | 18.388(3) | 12.4009(11) | 10.449(2) |
| $\alpha$ (°) | 100.620(3) | 86.068(4) | 81.088(3) | 90 |
| $\beta$ (°) | 97.075(4) | 79.510(4) | 83.588(3) | 95.952(7) |
| $\gamma$ (°) | 108.592(3) | 65.123(3) | 67.300(2) | 90 |
| $V$ (Å$^3$) | 1118.3(2) | 1792.1(5) | 1143.34(18) | 1798.2(7) |
| $Z$ | 2 | 2 | 2 | 4 |
| $\rho_{calcd}$ (Mg m$^{-3}$) | 1.899 | 1.604 | 1.788 | 2.070 |
| $\mu$ (mm$^{-1}$) | 6.310 | 4.145 | 6.165 | 7.831 |
| R1$^a$ | 0.0134 | 0.0377 | 0.0133 | 0.0173 |
| wR2$^b$ | 0.0361 | 0.0829 | 0.0342 | 0.0389 |

$^a$R1 = $\Sigma||F_o| - |F_c||/\Sigma|F_o|$ for reflections with $I > 2\sigma(I)$. $^b$wR2 = $[\Sigma[w(F_o^2 - F_c^2)^2/\Sigma[w(F_o^2)^2]]^{0.5}$ for all reflections; $w^{-1} = \sigma^2(F^2) + (aP)^2 + bP$, where $P = (2F_c^2 + F_o^2)/3$ and $a$ and $b$ are constants set by the program.

**Table S2.** Crystallographic data for **11**, **14**, and **15**.

| | **11** | **14** | **15** |
|---|---|---|---|
| formula | $C_{36}H_{32}N_4O_2Pt$ | $C_{34}H_{28}N_2Pt$ | $C_{26}H_{19}NOPt$ |
| fw | 747.74 | 659.67 | 556.51 |
| $T$ (K) | 100(2) | 100(2) | 100(2) |
| λ | 0.71073 | 0.71073 | 0.71073 |
| cryst syst | monoclinic | monoclinic | monoclinic |
| space group | $P2_1/n$ | $P2_1/n$ | $P2_1/c$ |
| $a$ (Å) | 14.2124(17) | 13.5434(7) | 12.792(3) |
| $b$ (Å) | 14.6485(13) | 7.7317(3) | 7.7959(14) |
| $c$ (Å) | 15.7085(18) | 24.4410(14) | 19.751(4) |
| $\alpha$ (°) | 90 | 90 | 90 |
| $\beta$ (°) | 114.785(3) | 96.677(2) | 99.827(6) |
| $\gamma$ (°) | 90 | 90 | 90 |
| $V$ (Å$^3$) | 2969.1(6) | 2541.9(2) | 1940.7(6) |
| $Z$ | 4 | 4 | 4 |
| $\rho_{calcd}$ (Mg m$^{-3}$) | 1.673 | 1.724 | 1.905 |
| $\mu$ (mm$^{-1}$) | 4.766 | 5.547 | 7.247 |
| R1$^a$ | 0.0153 | 0.0145 | 0.0199 |
| wR2$^b$ | 0.0355 | 0.0344 | 0.0494 |

$^a$R1 = $\Sigma||F_o| - |F_c||/\Sigma|F_o|$ for reflections with $I > 2\sigma(I)$. $^b$wR2 = $[\Sigma[w(F_o^2 - F_c^2)^2/\Sigma[w(F_o^2)^2]]^{0.5}$ for all reflections; $w^{-1} = \sigma^2(F^2) + (aP)^2 + bP$, where $P = (2F_c^2 + F_o^2)/3$ and $a$ and $b$ are constants set by the program.

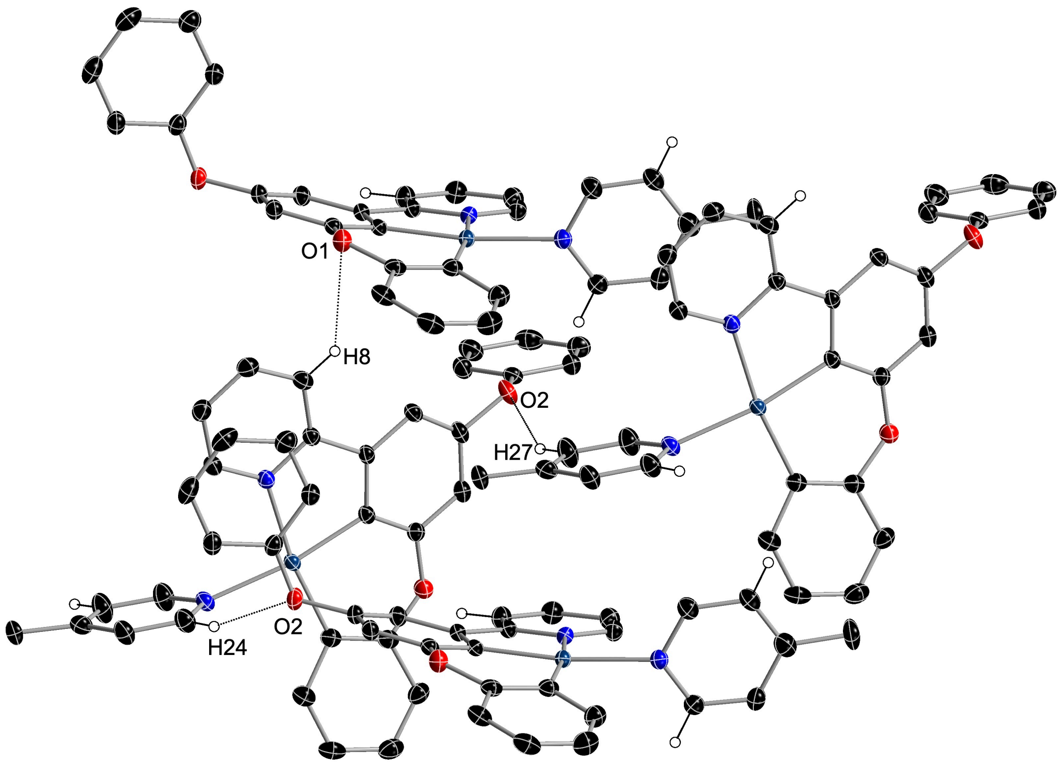


**Figure S16.** Crystal packing in the structure of **3**, showing intermolecular C–H···O contacts (thermal ellipsoids at 50% probability). Hydrogen atoms are omitted, except for those involved in the contacts. Distances: O1···H8, 2.597 Å; O2···H24, 2.477 Å; O2···H27, 2.630 Å.

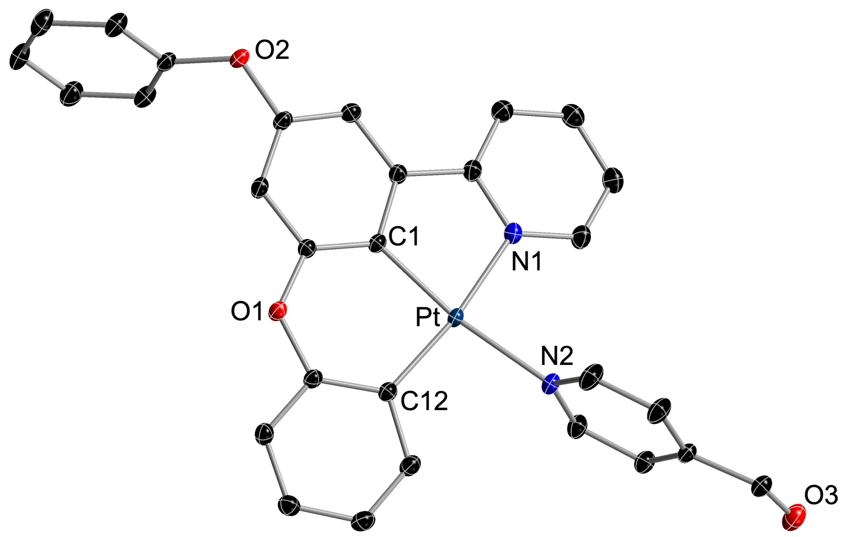


**Figure S17.** Structure of **4** in the crystal (thermal ellipsoids at 50% probability). Hydrogen atoms are omitted.

**Table S3.** Selected bond distances (Å) and angles (°) for **4**.

| | | | |
|---|---|---|---|
| Pt–C1 | 1.9593(16) | Pt–N2 | 2.1157(14) |
| Pt–C12 | 1.9952(17) | C29–O3 | 1.200(2) |
| Pt–N1 | 2.1019(15) | | |
| | | | |
| C1–Pt–N1 | 80.76(6) | C12–Pt–N2 | 94.67(6) |
| C1–Pt–C12 | 91.90(7) | C1–Pt–N2 | 170.83(6) |
| N1–Pt–N2 | 93.18(6) | C12–Pt–N1 | 171.22(6) |

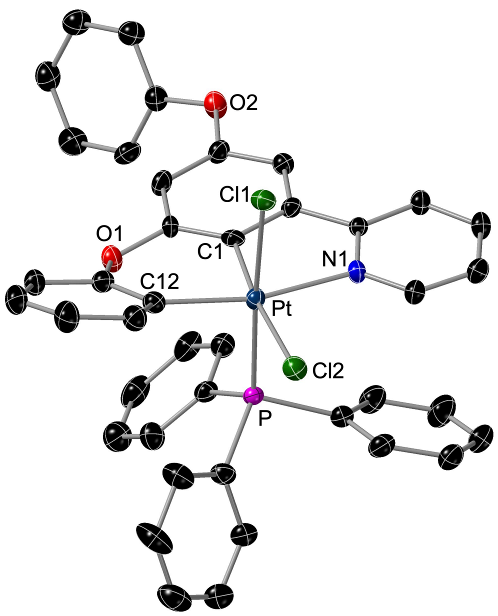


**Figure S18.** Structure of **6** in the crystal (thermal ellipsoids at 50% probability). Hydrogen atoms are omitted.

**Table S4.** Selected bond distances (Å) and angles (°) for **6**.

| | | | |
|---|---|---|---|
| Pt-C1 | 2.024(5) | Pt-P | 2.3156(12) |
| Pt-C12 | 2.047(5) | Pt-Cl1 | 2.3900(11) |
| Pt-N1 | 2.147(4) | Pt-Cl2 | 2.4288(11) |
| | | | |
| C1-Pt-C12 | 89.57(18) | N1-Pt-Cl1 | 84.39(10) |
| C1-Pt-N1 | 80.54(16) | P-Pt-Cl1 | 176.97(4) |
| C12-Pt-N1 | 167.25(16) | C1-Pt-Cl2 | 173.86(13) |
| C1-Pt-P | 92.07(12) | C12-Pt-Cl2 | 96.47(13) |
| C12-Pt-P | 94.95(13) | N1-Pt-Cl2 | 93.33(10) |
| N1-Pt-P | 93.43(10) | P-Pt-Cl2 | 88.53(4) |
| C1-Pt-Cl1 | 89.65(12) | Cl1-Pt-Cl2 | 89.50(4) |
| C12-Pt-Cl1 | 87.55(13) | | |

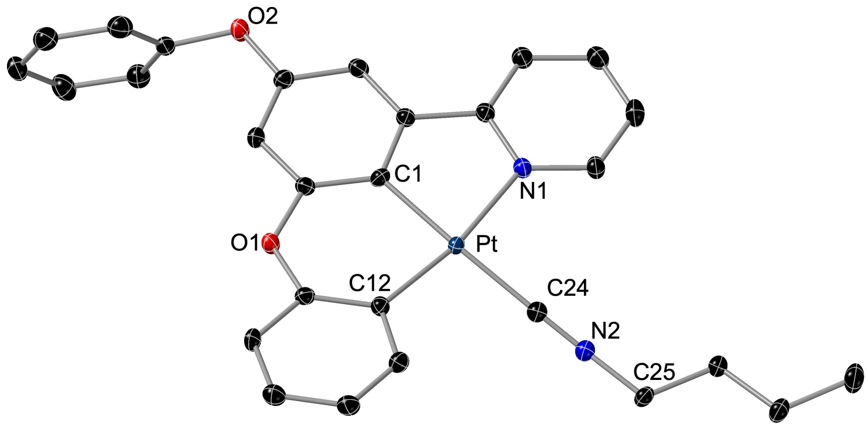


**Figure S19.** Structure of **7** in the crystal (thermal ellipsoids at 50% probability). Hydrogen atoms are omitted.

**Table S5.** Selected bond distances (Å) and angles (°) for **7**.

| | | | |
|---|---|---|---|
| Pt–C1 | 1.9928(17) | Pt–C24 | 1.9784(18) |
| Pt–C12 | 2.0126(18) | C24–N2 | 1.153(2) |
| Pt–N1 | 2.0975(15) | | |
| | | | |
| C1–Pt–N1 | 80.07(7) | C12–Pt–C24 | 93.62(7) |
| C1–Pt–C12 | 90.92(7) | C1–Pt–C24 | 173.72(7) |
| N1–Pt–C24 | 95.62(7) | C12–Pt–N1 | 170.41(6) |

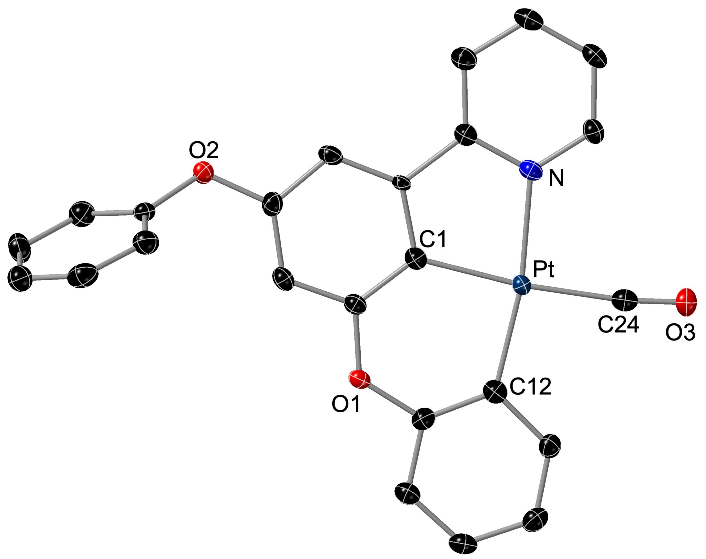


**Figure S20.** Structure of **9** in the crystal (thermal ellipsoids at 50% probability). Hydrogen atoms are omitted.

**Table S6.** Selected bond distances (Å) and angles (°) for **9**.

| | | | |
|---|---|---|---|
| Pt–C1 | 1.993(3) | Pt–C24 | 1.910(3) |
| Pt–C12 | 2.017(3) | C24–O3 | 1.129(4) |
| Pt–N | 2.108(2) | | |
| | | | |
| C1–Pt–N | 80.25(11) | C12–Pt–C24 | 94.96(13) |
| C1–Pt–C12 | 90.65(13) | C1–Pt–C24 | 174.35(13) |
| N–Pt–C24 | 94.13(12) | C12–Pt–N | 170.85(11) |

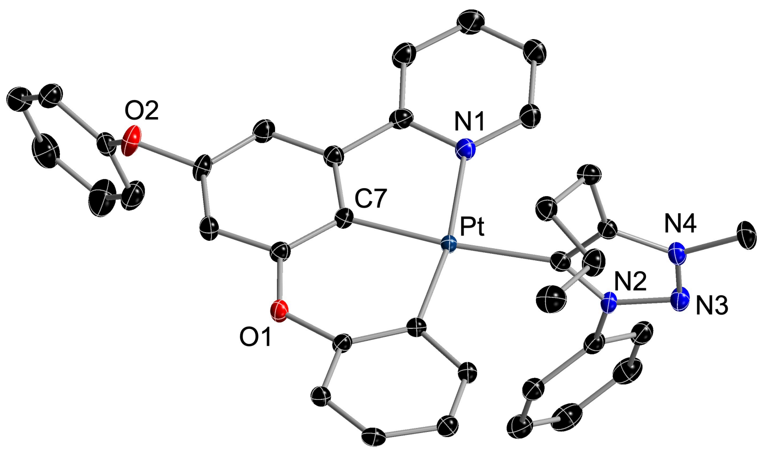


**Figure S21.** Structure of **11** in the crystal (thermal ellipsoids at 50% probability).

**Table S7.** Selected bond distances (Å) and angles (°) for **11**.

| | | | |
|---|---|---|---|
| Pt–C1 | 1.993(2) | Pt–C24 | 2.060(2) |
| Pt–C12 | 2.005(2) | Pt–N1 | 2.1026(18) |
| | | | |
| C1–Pt–N1 | 80.35(8) | C12–Pt–C24 | 95.17(8) |
| C1–Pt–C12 | 90.78(8) | C1–Pt–C24 | 174.00(8) |
| N1–Pt–C24 | 93.67(8) | C12–Pt–N1 | 170.37(8) |

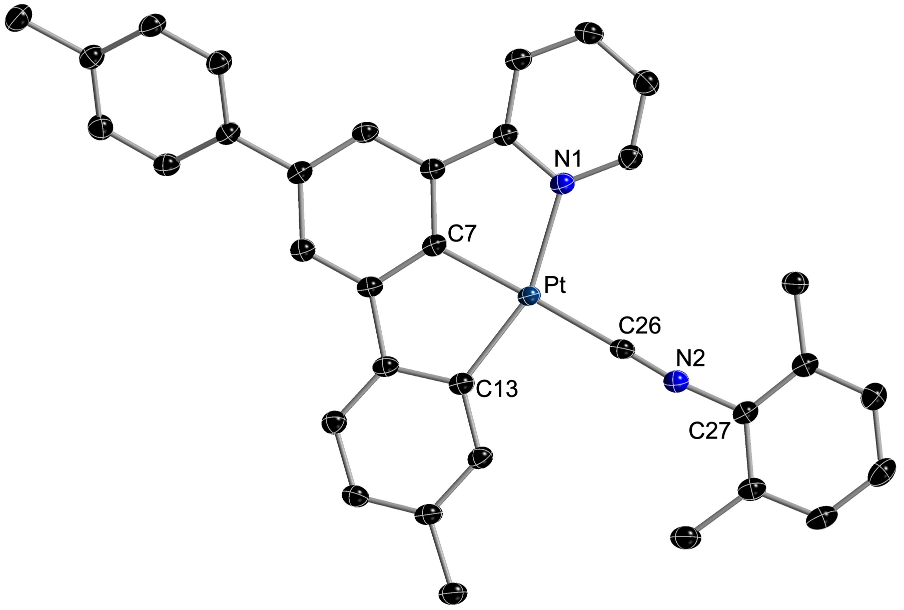


**Figure S22.** Structure of **14** in the crystal (thermal ellipsoids at 50% probability). Hydrogen atoms are omitted.

**Table S8.** Selected bond distances (Å) and angles (°) for **14**.

| | | | |
|---|---|---|---|
| Pt-C7 | 1.9662(18) | Pt-N1 | 2.1349(16) |
| Pt-C26 | 1.9758(19) | N2-C26 | 1.157(2) |
| Pt-C13 | 2.0166(19) | N2-C27 | 1.397(2) |
| | | | |
| C7-Pt-C26 | 177.63(7) | C26-Pt-N1 | 102.60(7) |
| C7-Pt-C13 | 80.30(8) | C13-Pt-N1 | 159.00(7) |
| C26-Pt-C13 | 98.21(7) | N2-C26-Pt | 178.87(17) |
| C7-Pt-N1 | 78.81(7) | C26-N2-C27 | 173.49(19) |

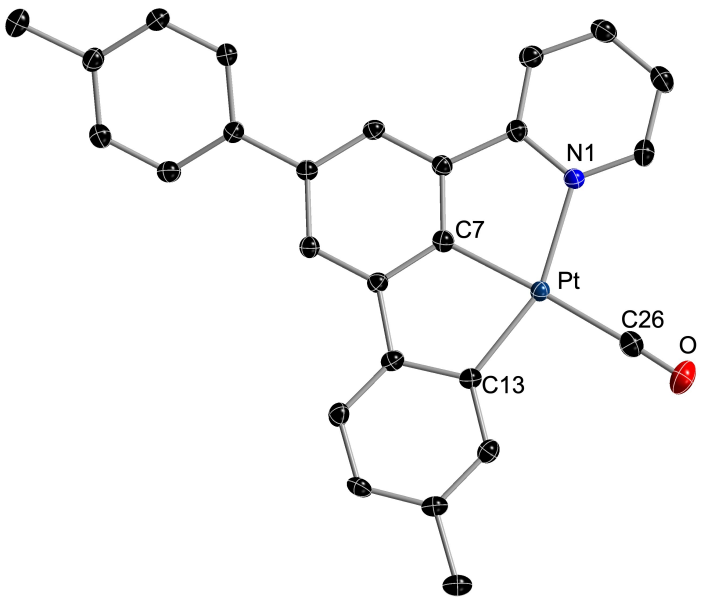


**Figure S23.** Structure of **15** in the crystal (thermal ellipsoids at 50% probability). Hydrogen atoms are omitted.

**Table S9.** Selected bond distances (Å) and angles (°) for **15**.

| | | | |
|---|---|---|---|
| Pt-C26 | 1.930(3) | Pt-N1 | 2.136(3) |
| Pt-C7 | 1.977(3) | Pt-Pt#1 | 3.2219(5) |
| Pt-C13 | 2.029(3) | C26-O1 | 1.129(4) |
| | | | |
| C26-Pt-C7 | 176.21(13) | C7-Pt-N1 | 78.43(11) |
| C26-Pt-C13 | 96.56(13) | C13-Pt-N1 | 158.49(11) |
| C7-Pt-C13 | 80.22(12) | O1-C26-Pt | 176.4(3) |
| C26-Pt-N1 | 104.67(12) | | |

**Table S10.** Characterization of the π stacking interactions observed in the crystal structures of complexes **7**, **9**, **11**, **14** and **15**.

| Complex | Angle between mean ring planes (°) | Centroid-centroid distance (Å) | Shift distance (Å) |
|---|---|---|---|
| **7** | 2.68 | 3.547 | 1.156 |
| **9** | 1.63 | 3.552 | 0.996 |
| | 1.63 | 3.549 | 1.072 |
| **11** | 0 | 3.768 | 1.649 |
| **14** | 2.50 | 3.988 | 2.038 |
| | 4.39 | 3.971 | 1.793 |
| **15** | 3.45 | 3.619 | 1.433 |

## 2. Additional photophysical data

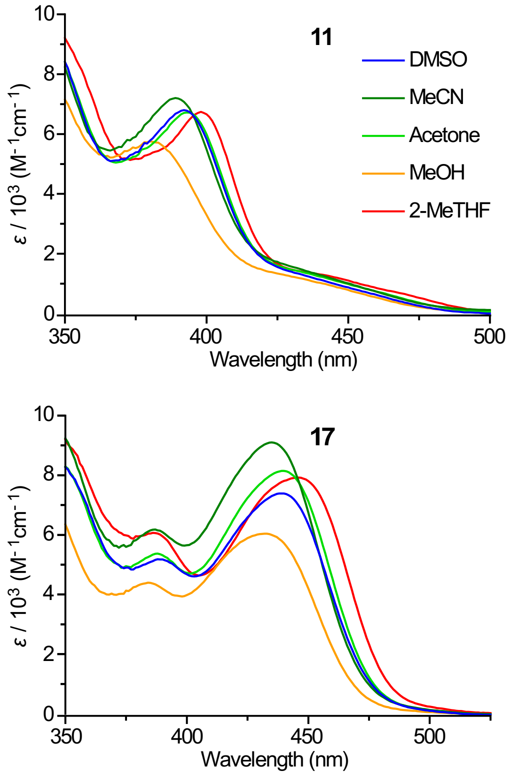


**Figure S24.** Electronic absorption spectra of complexes **11** and **17** in solvents of different polarity (*ca.* 5 × $10^{-5}$ M).

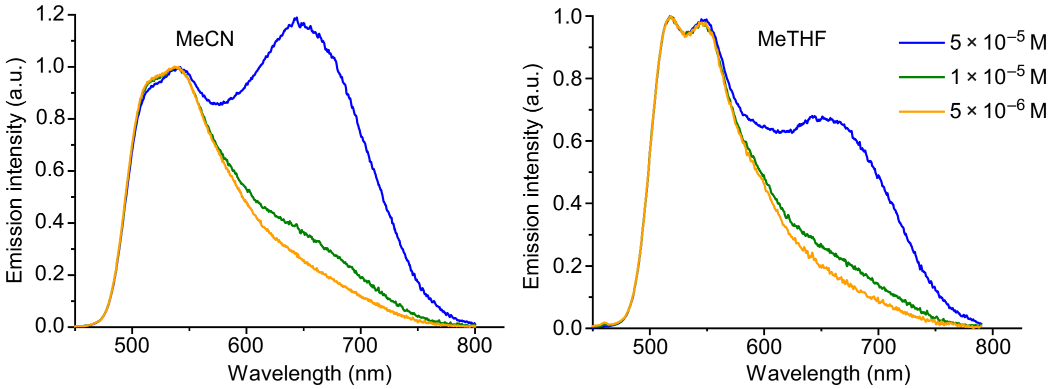


**Figure S25.** Emission spectra of complex **7** in MeCN and MeTHF solutions at different concentrations at 298 K. Emission intensities have been normalized at the most intense peak of the monomeric emission.

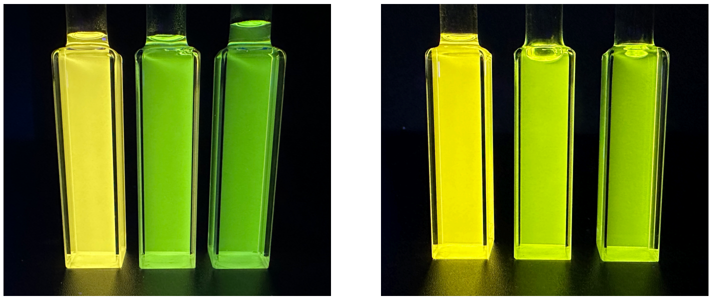

**Figure S26.** Photographs of MeCN (left) and MeTHF (right) solutions of complex **7** at $5 \times 10^{-5}$, $1 \times 10^{-5}$ and $5 \times 10^{-6}$ M concentrations (from left to right) under UV irradiation at 298 K.

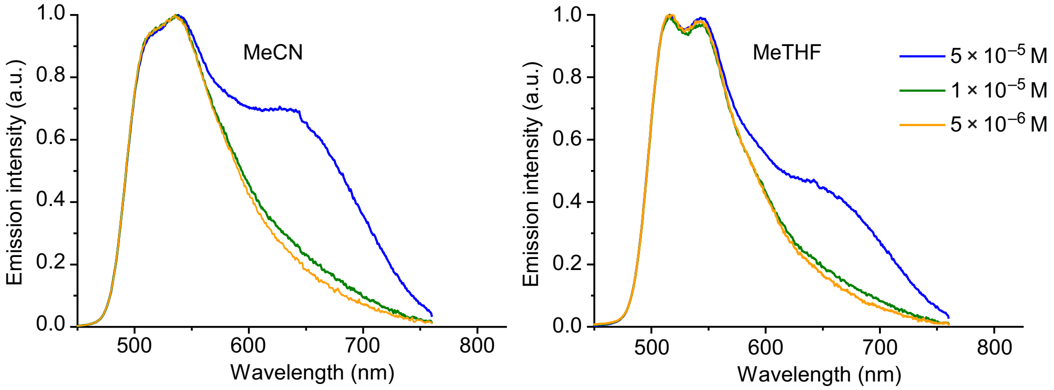


**Figure S27.** Emission spectra of complex **8** in MeCN and MeTHF solutions at different concentrations at 298 K.

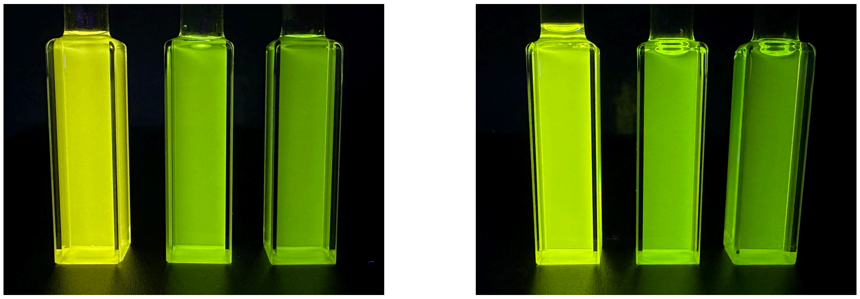

**Figure S28.** Photographs of MeCN (left) and MeTHF (right) solutions of complex **8** at 5 × 10$^{-5}$, 1 × 10$^{-5}$ and 5 × 10$^{-6}$ M concentrations (from left to right) under UV irradiation at 298 K.

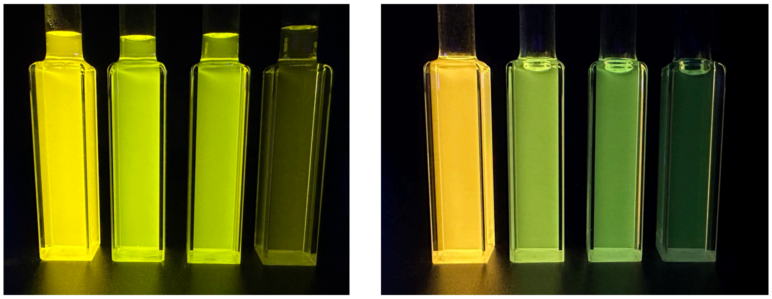

**Figure S29.** Photographs of MeCN (left) and MeTHF (right) solutions of complex **9** at 5 × 10$^{-5}$, 1 × 10$^{-5}$ and 5 × 10$^{-6}$ M and 1 × 10$^{-6}$ M concentrations (from left to right) under UV irradiation at 298 K.

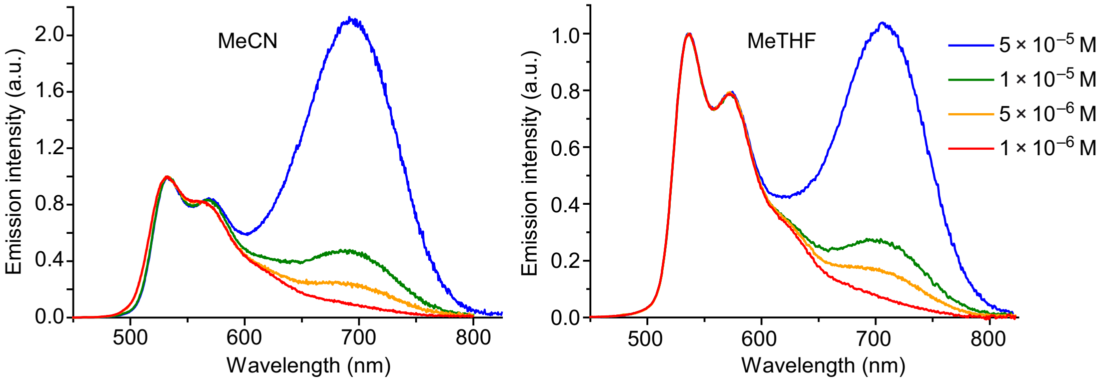


**Figure S30.** Emission spectra of complex **14** in MeCN and MeTHF solution at different concentrations at 298 K. Emission intensities have been normalized at the most intense peak of the monomeric emission.

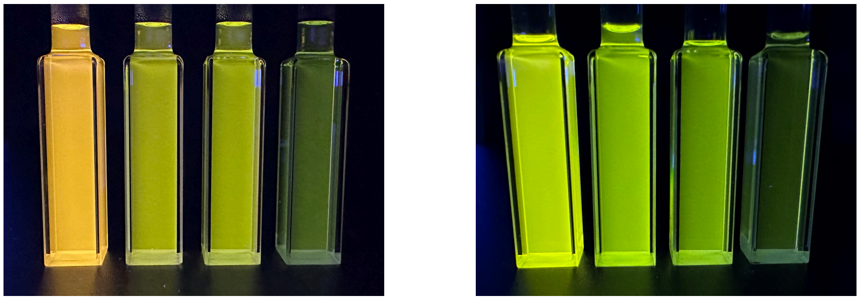

**Figure S31.** Photographs of MeCN (left) and MeTHF (right) solutions of complex **14** at $5 \times 10^{-5}$, $1 \times 10^{-5}$, $5 \times 10^{-6}$ and $1 \times 10^{-6}$ M concentrations (from left ro right) at 298 K.

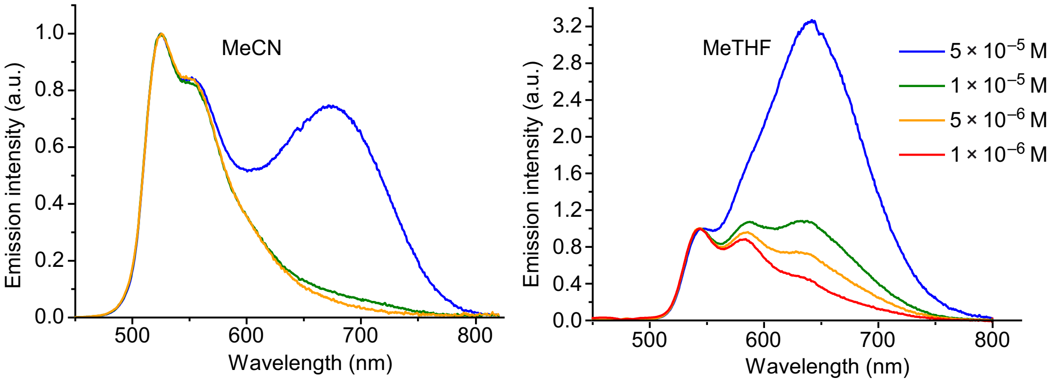


**Figure S32.** Emission spectra of complex **15** in MeCN and MeTHF solution at different concentrations at 298 K. Emission intensities have been normalized at the most intense peak of the monomeric emission.

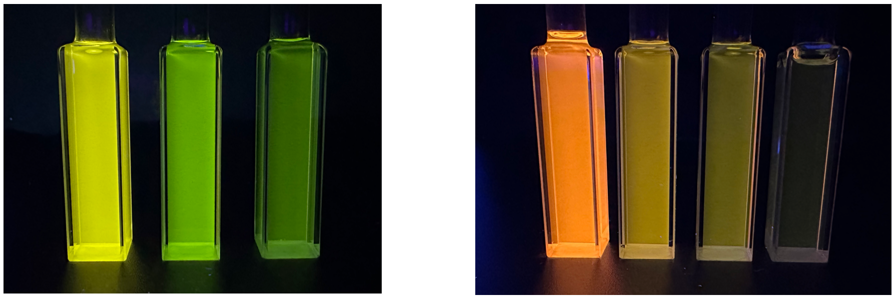

**Figure S33.** Photographs of MeCN (left) and MeTHF (right) solutions of complex **15** at $5 \times 10^{-5}$, $1 \times 10^{-5}$ and $5 \times 10^{-6}$ M concentrations (MeCN) or $5 \times 10^{-5}$, $1 \times 10^{-5}$, $5 \times 10^{-6}$ and $1 \times 10^{-6}$ M (MeTHF) concentrations (from left to right) at 298 K.

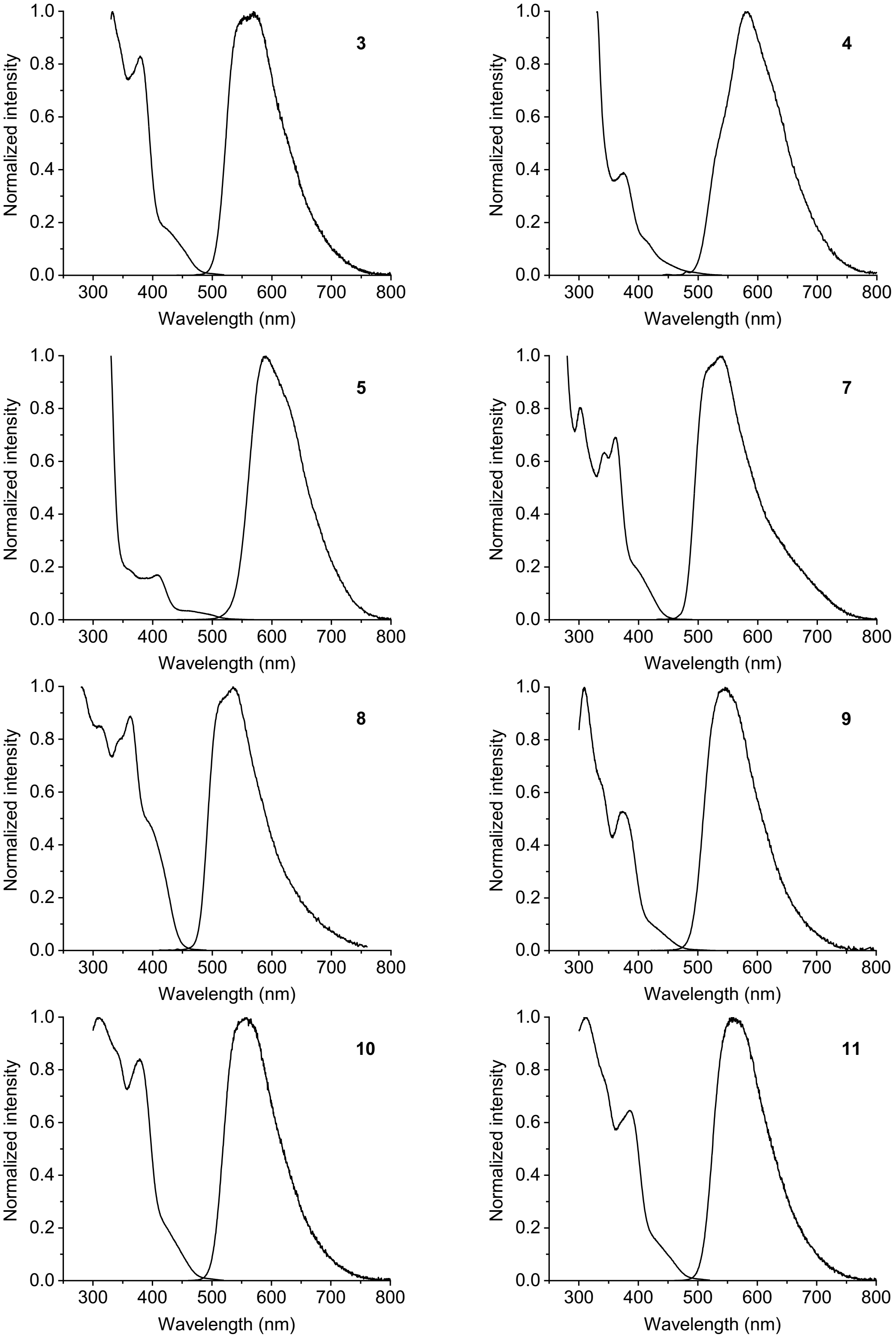


**Figure S34.** Excitation and emission spectra in acetone (complexes **3–5**) or MeCN (complexes **7–11**) solution at 298 K.

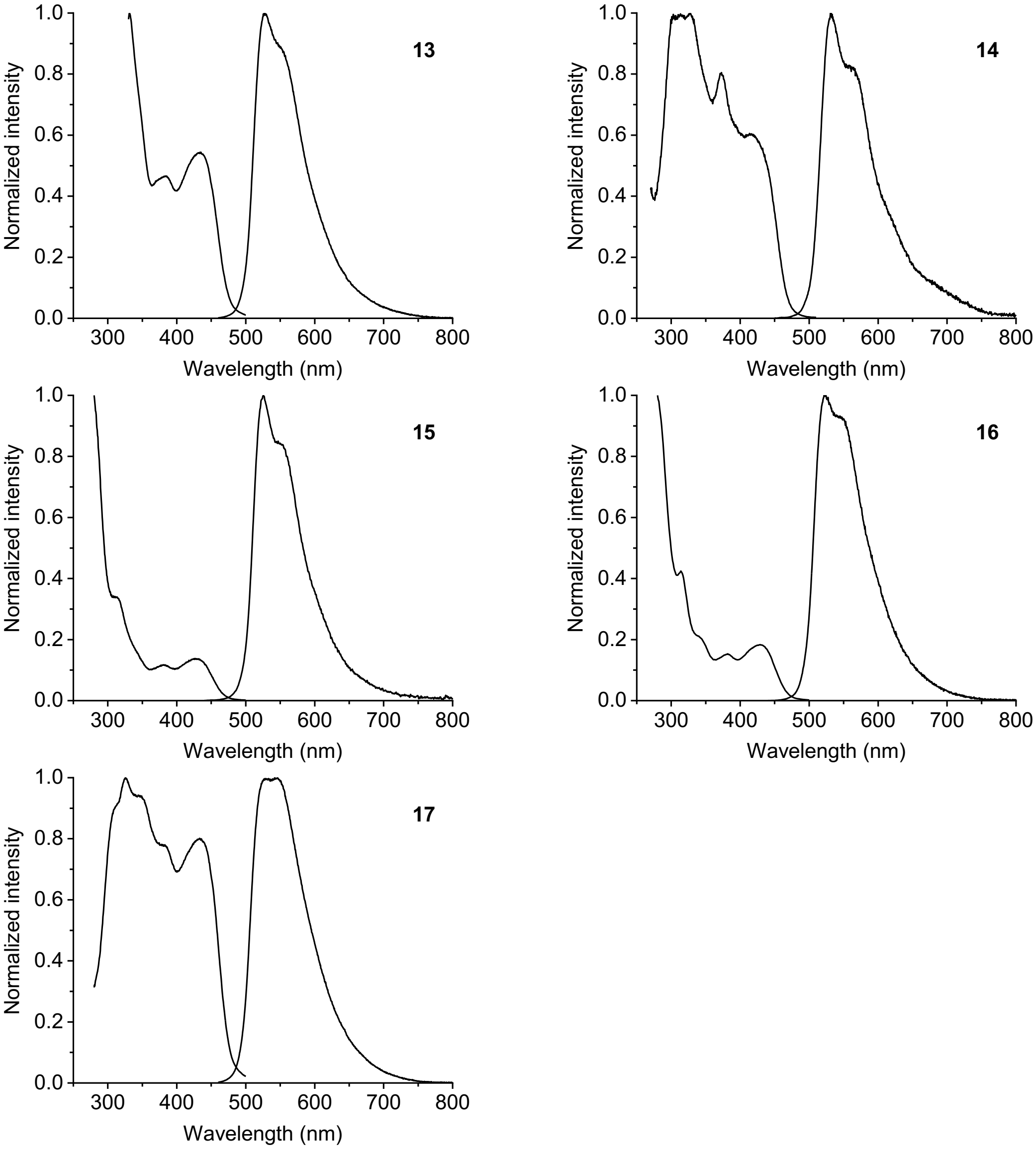


**Figure S35.** Excitation and emission spectra in acetone (complex **13**) or MeCN (complexes **14**–**17**) solution at 298 K.

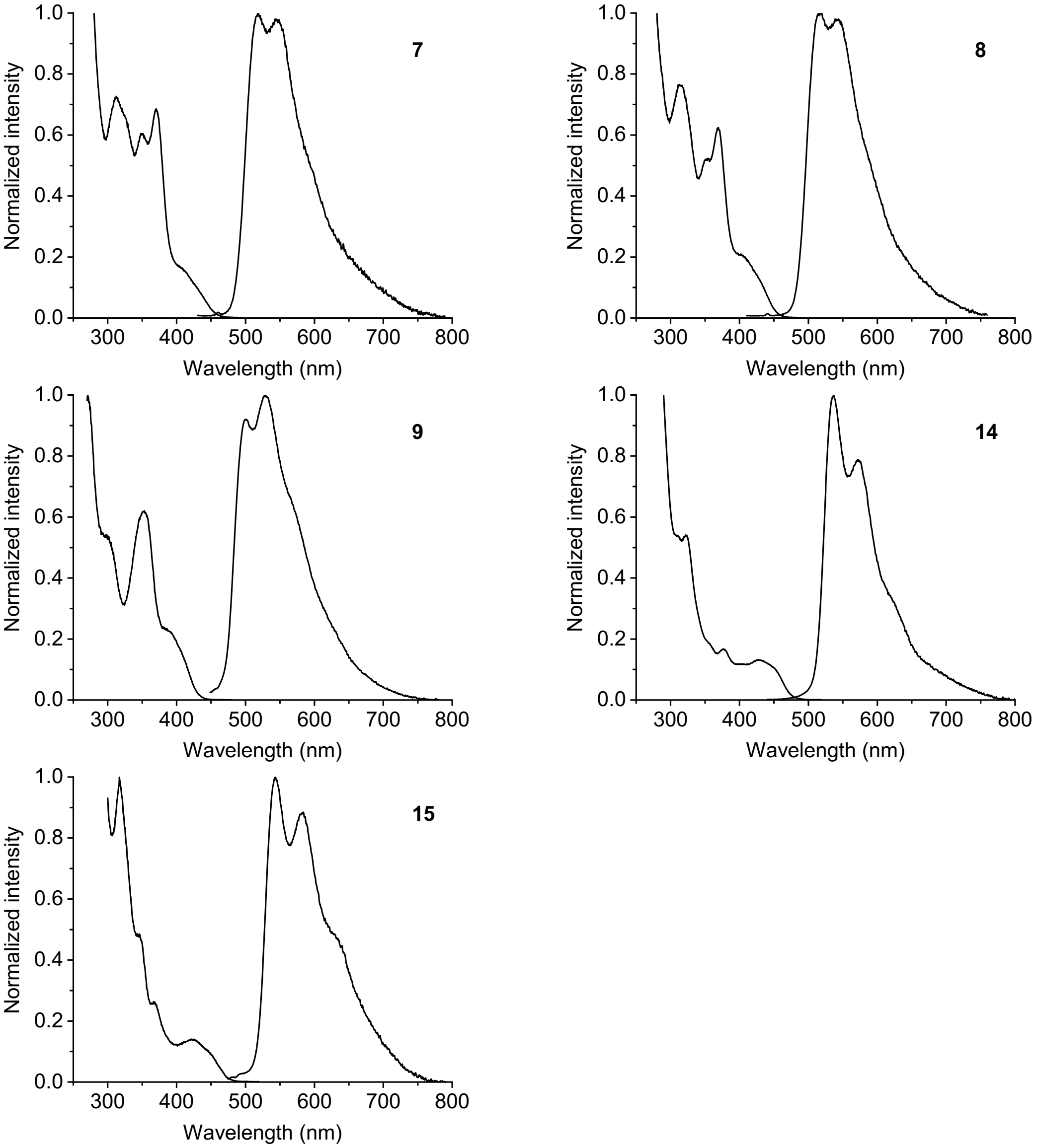


**Figure S36.** Excitation and emission spectra of complexes **7–9**, **14** and **15** in 2-methyltetrahydrofuran solution at 298 K.

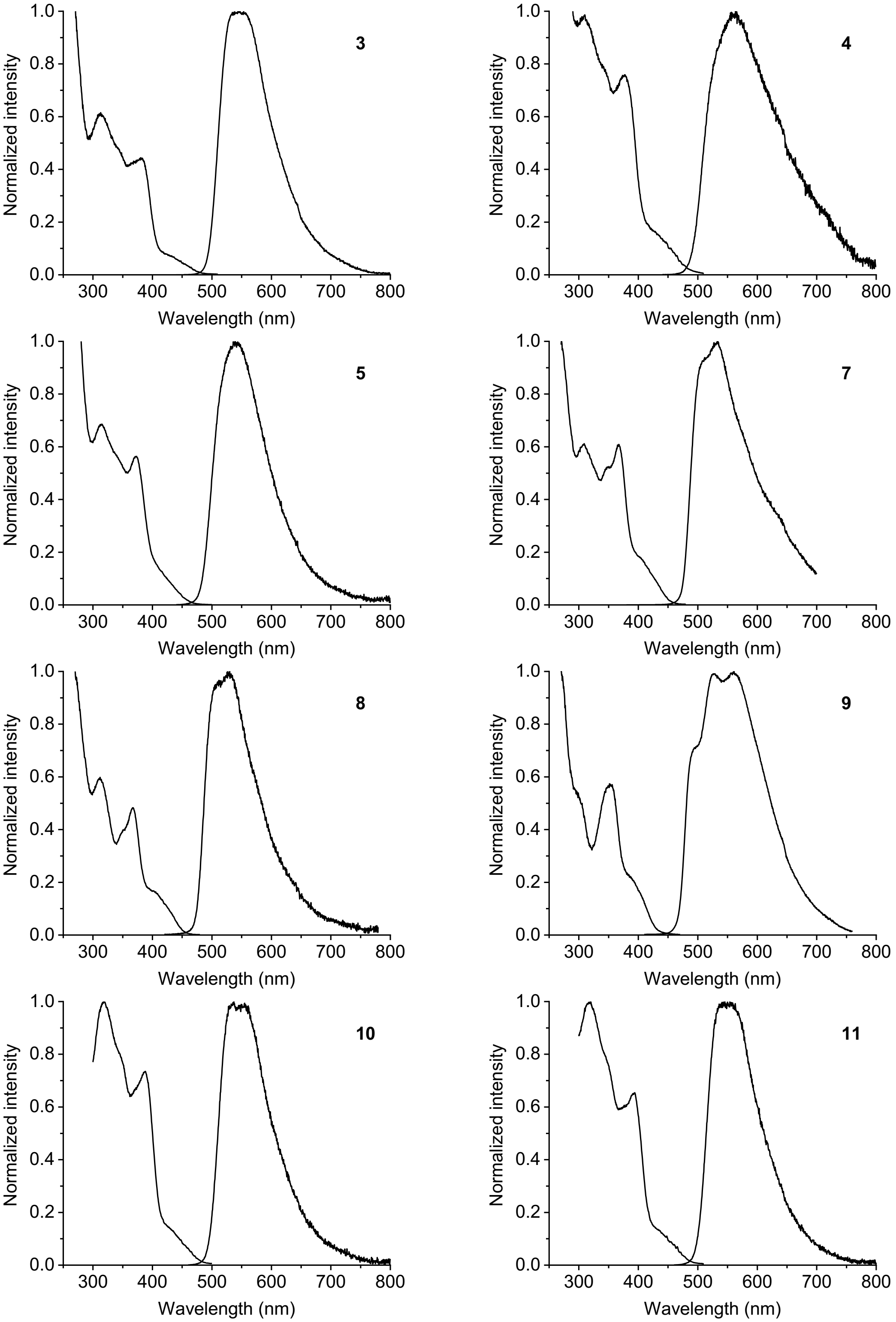


**Figure S37.** Excitation and emission spectra of complexes **3–5** and **7–11** in PMMA matrices (2 wt%) at 298 K.

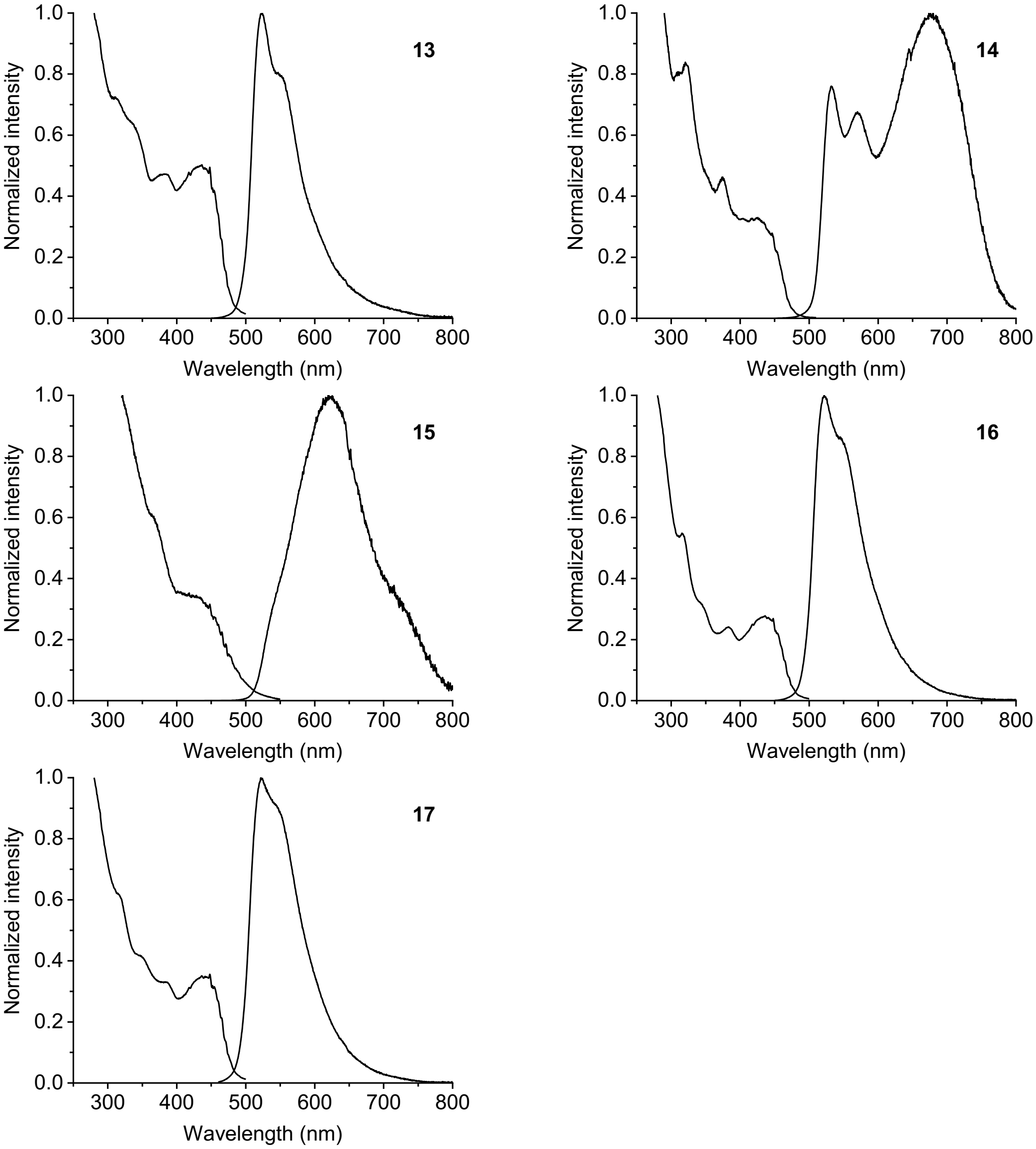


**Figure S38.** Excitation and emission spectra of complexes **13**–**17** in PMMA matrices (2 wt%) at 298 K.

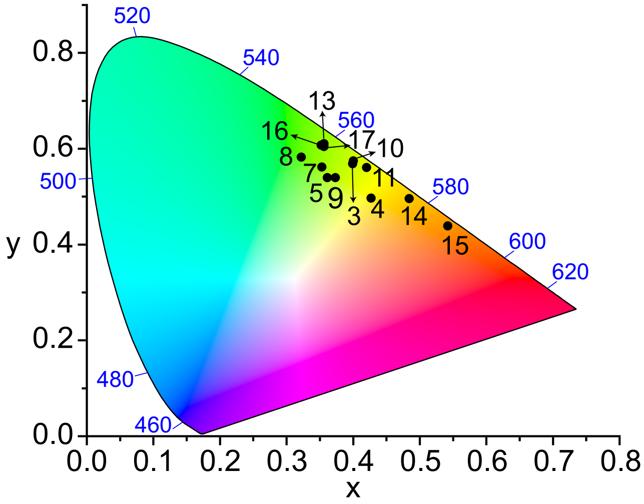


**Figure S39.** Commission Internationale de l'éclairage coordinates (CIE 1931) of the studied complexes in PMMA matrices (2 wt%).

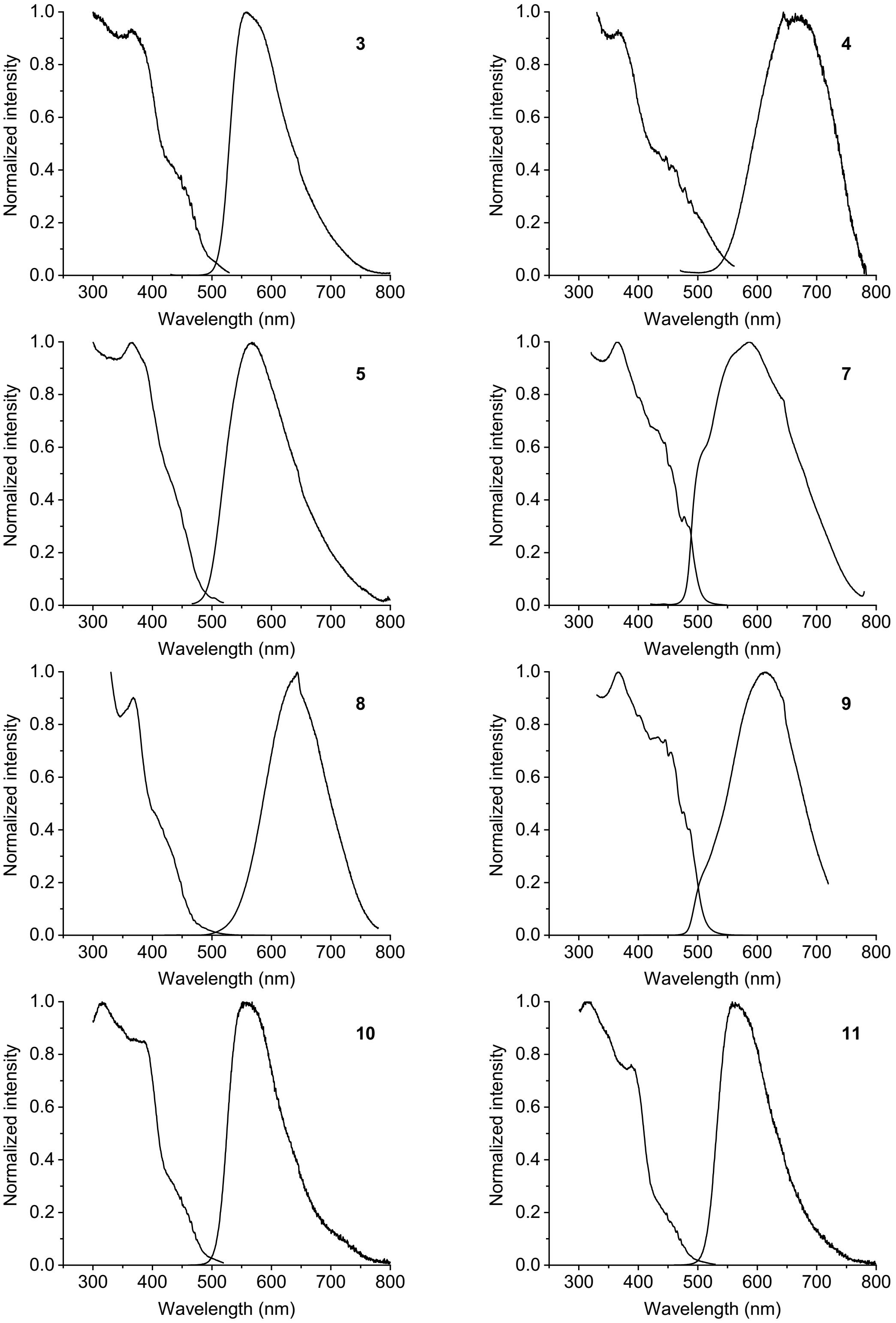


**Figure S40.** Excitation and emission spectra of complexes **3**–**5** and **7**–**11** in the solid state at 298 K.

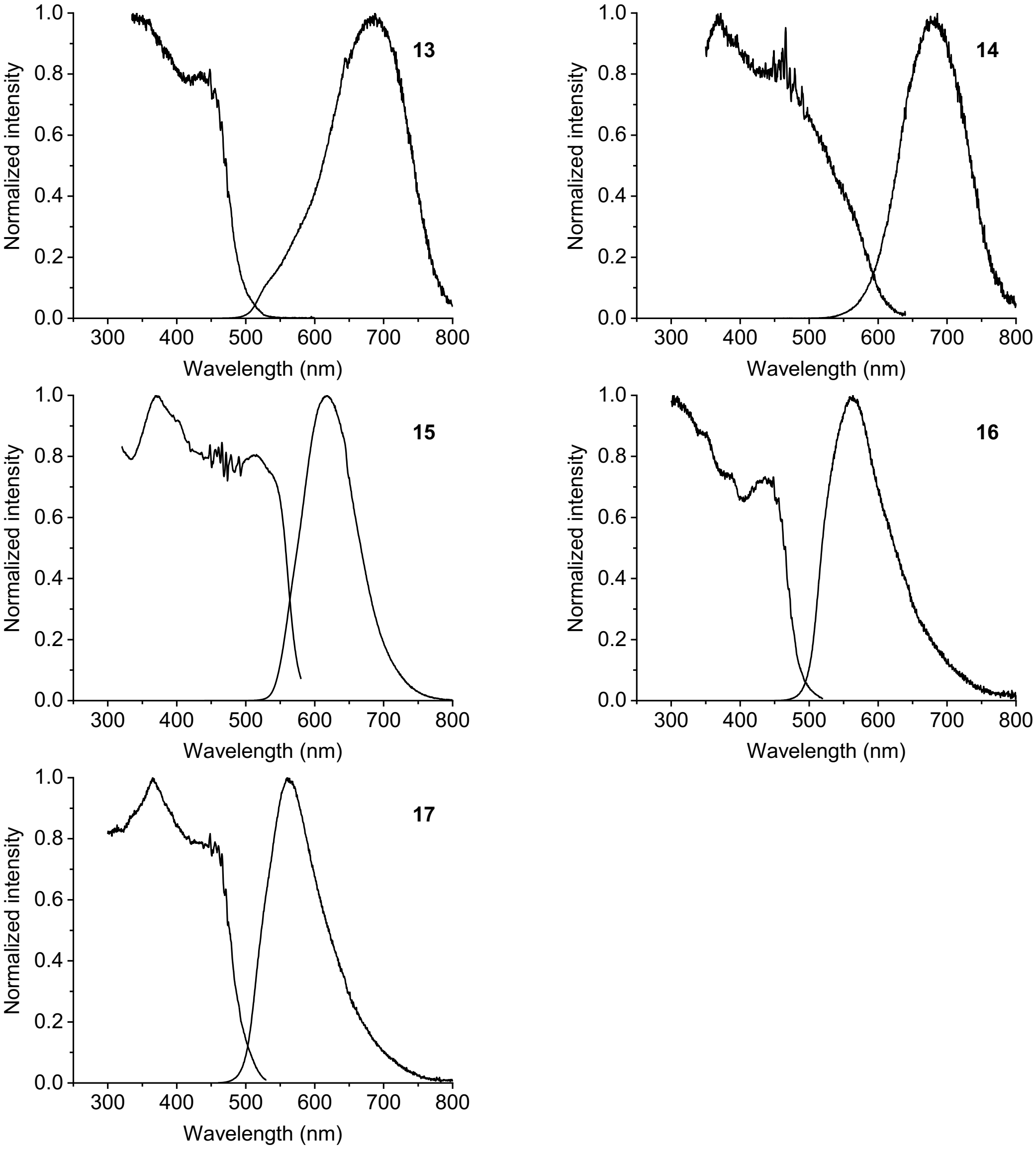

**Figure S41.** Excitation and emission spectra of complexes **13**–**17** in the solid state at 298 K.

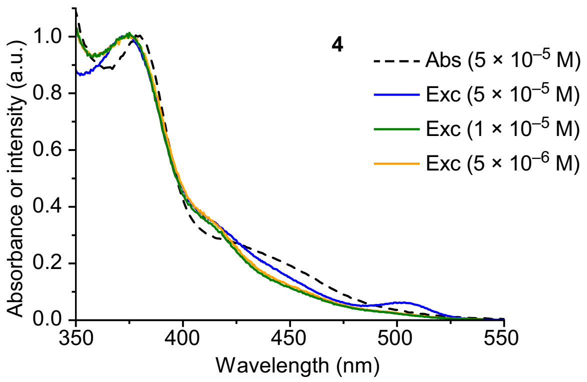


**Figure S42.** Absorption spectrum and excitation spectra at different concentrations of complex **4** in acetone solution at 298 K.

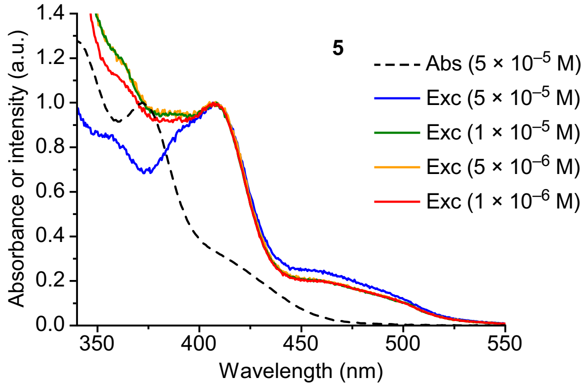


**Figure S43.** Absorption spectrum and excitation spectra at different concentrations of complex **5** in acetone solution at 298 K.

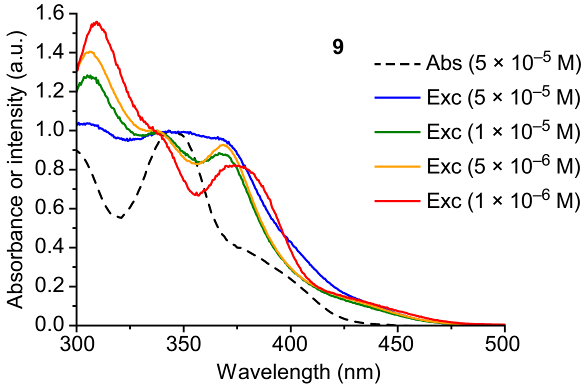


**Figure S44.** Absorption spectrum and excitation spectra at different concentrations of complex **9** in MeCN solution at 298 K.

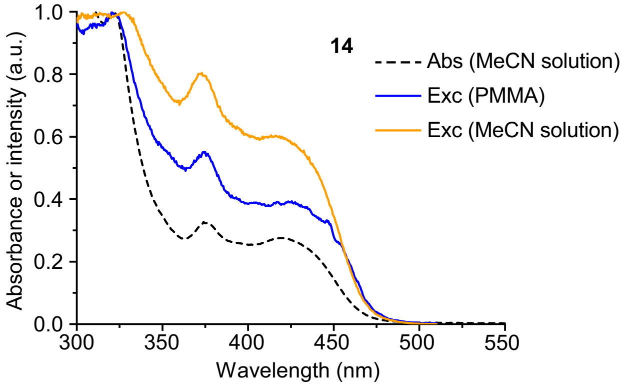


**Figure S45.** Absorption spectrum in MeCN solution and excitation spectra in MeCN solution and PMMA of complex **14** at 298 K.

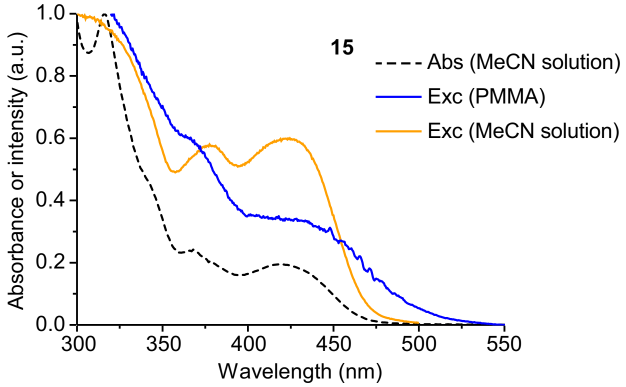


**Figure S46.** Absorption spectrum in MeCN solution and excitation spectra in MeCN solution and PMMA of complex **15** at 298 K.

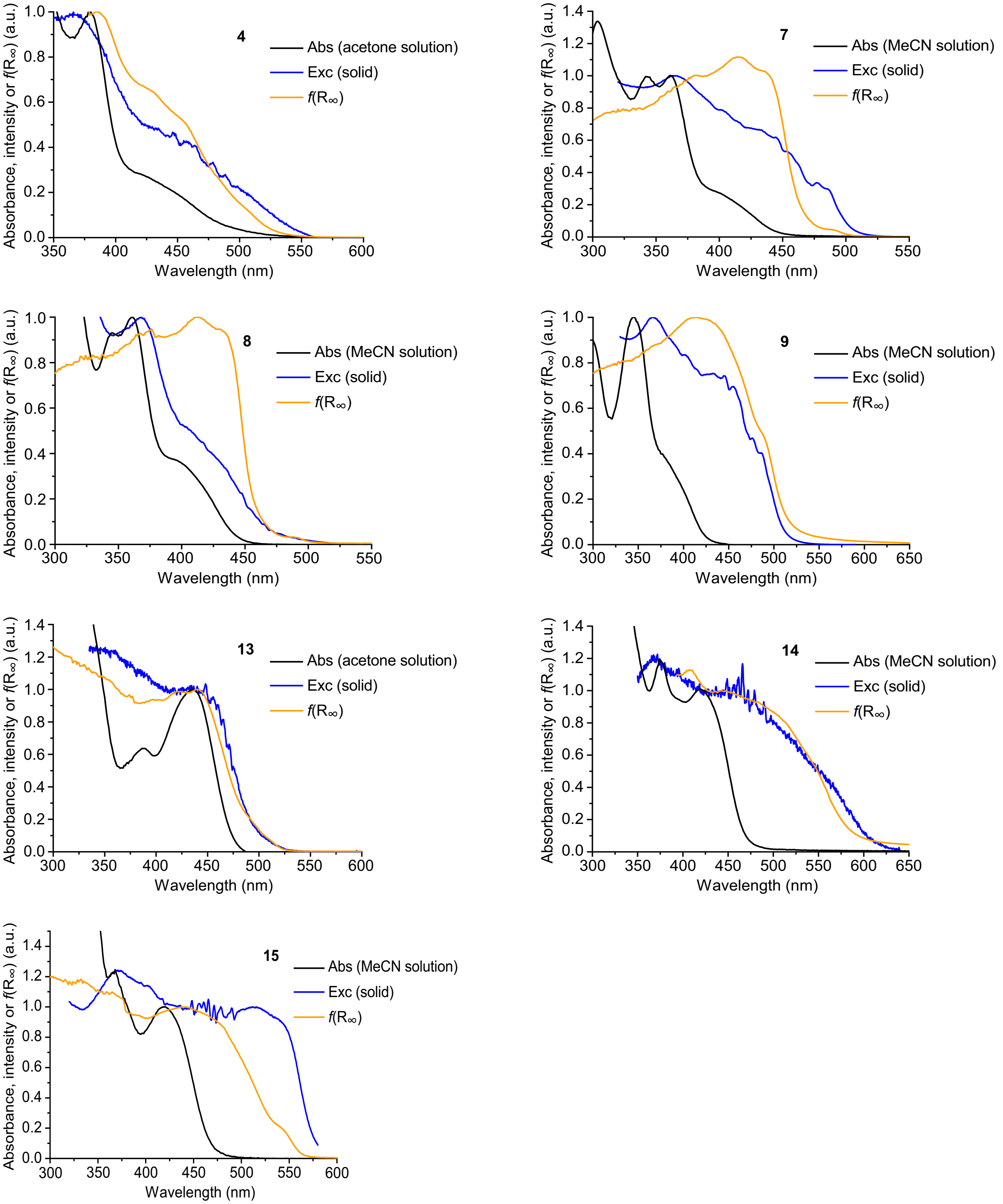


**Figure S47.** Comparisons of absorption spectra in solution, excitation spectra in the solid state and diffuse reflectance spectra (Kubelka-Munk function, $f(R_\infty)$) for complexes **4**, **7**–**9** and **13**–**15** at 298 K.

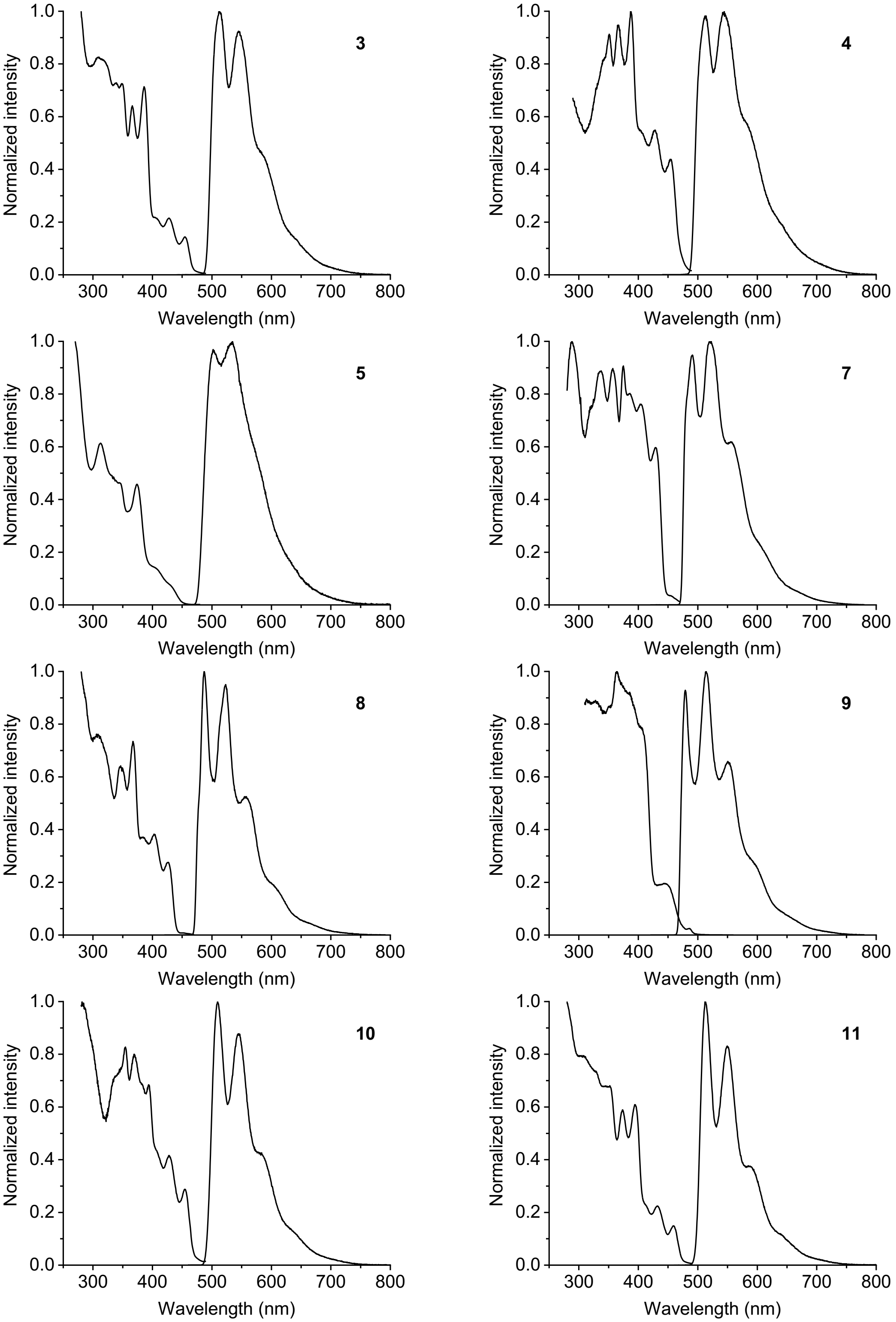


**Figure S48.** Excitation and emission spectra of complexes **3**–**5** and **7**–**11** in 2-methyltetrahydrofuran glasses at 77 K.

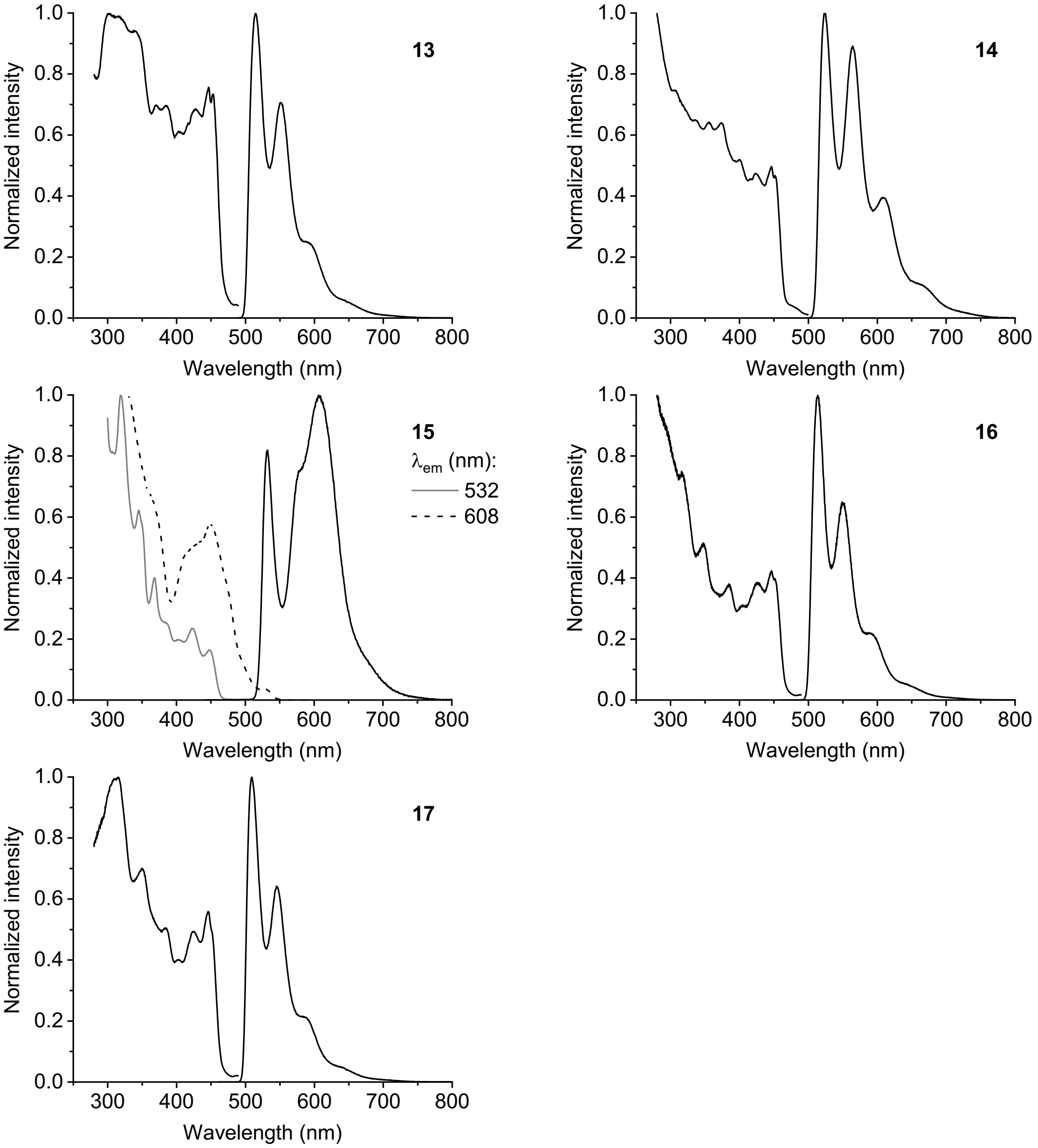


**Figure S49.** Excitation and emission spectra of complexes **13**–**17** in 2-methyltetrahydrofuran glasses at 77 K.

## 3. Electrochemical measurements

Cyclic voltammograms were registered at 298 K using a potentiostat/galvanostat AUTOLAB-100 (Echo-Chemie, Utrecht) and a three-electrode electrochemical cell equipped with a glassy carbon working electrode (Metrohm, 2 mm diameter), an Ag/AgCl/3 M KCl electrode reference, and a glassy carbon rod counter electrode. Degassed 1 mM solutions of the complexes in extra-dry MeCN (Acros Organics) and 0.1 M $(Bu_4N)PF_6$ as the electrolyte under an argon atmosphere were employed. The working electrode was polished with alumina slurry (0.05 μm) and rinsed with water and acetone before each experiment. The electrodes were activated electrochemically in the background solution by means of several voltammetric cycles at 1 V $s^{-1}$ between –2.8 V and 2.2 V. The reference electrode was checked against the $Fc^+$/Fc redox couple. Potentials are given against the standard calomel electrode (SCE).

The HOMO/LUMO energies were estimated from the onset values of the oxidation and reduction waves, respectively, referenced against $Fc^+$/Fc (0.40 V vs SCE in MeCN), using a formal potential of 5.1 eV for the $Fc^+$/Fc couple in the Fermi scale:[5]

$$E_{\mathrm{HOMO}} = -(E_{onset,ox} + 5.1 - 0.4)\ \mathrm{eV};\ E_{\mathrm{LUMO}} = -(E_{onset,red} + 5.1 - 0.4)\ \mathrm{eV}$$

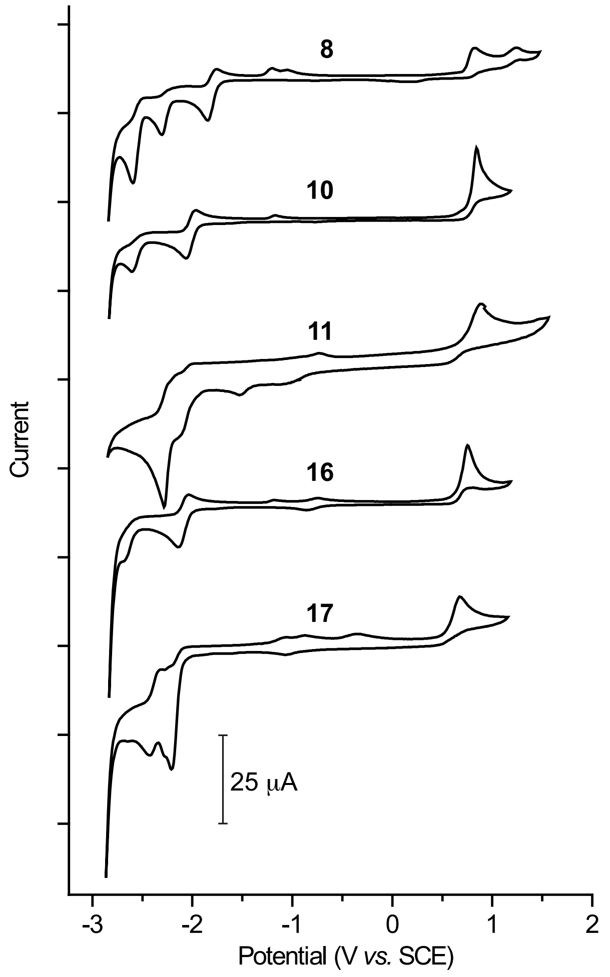


**Figure S50.** Cyclic voltammograms of complexes **8**, **10**, **11**, **16**, and **17** in MeCN at 10 mV $s^{-1}$.

## 4. Computational methods and data

DFT calculations were carried out with Gaussian 16,[7] using the B3LYP functional[8,9] together with the 6-31G**[10,11] basis set for the C, H, N, and O atoms and the LANL2DZ[12] basis set and effective core potential for the Pt atom. Geometry optimizations were performed with no restrictions on symmetry. Vertical singlet and triplet excitations were obtained from TDDFT calculations at the ground-state geometry; singlet excitations with oscillator strengths lower than 0.01 are omitted in the listings. Triplet excited-state geometries were obtained through a spin-unrestricted DFT (UB3LYP) optimization, following a previously described strategy.[13] The solvent effect (acetone or acetonitrile, as specified) was accounted for in all cases by using the SMD variation of the Polarizable Continuum Model, as implemented in Gaussian.[14] The optimized structures were confirmed as minima on the potential energy surface by performing frequency calculations (no imaginary frequencies).

## 4.1. Complex [Pt(dPhOppy)(γ-pic)] (3)

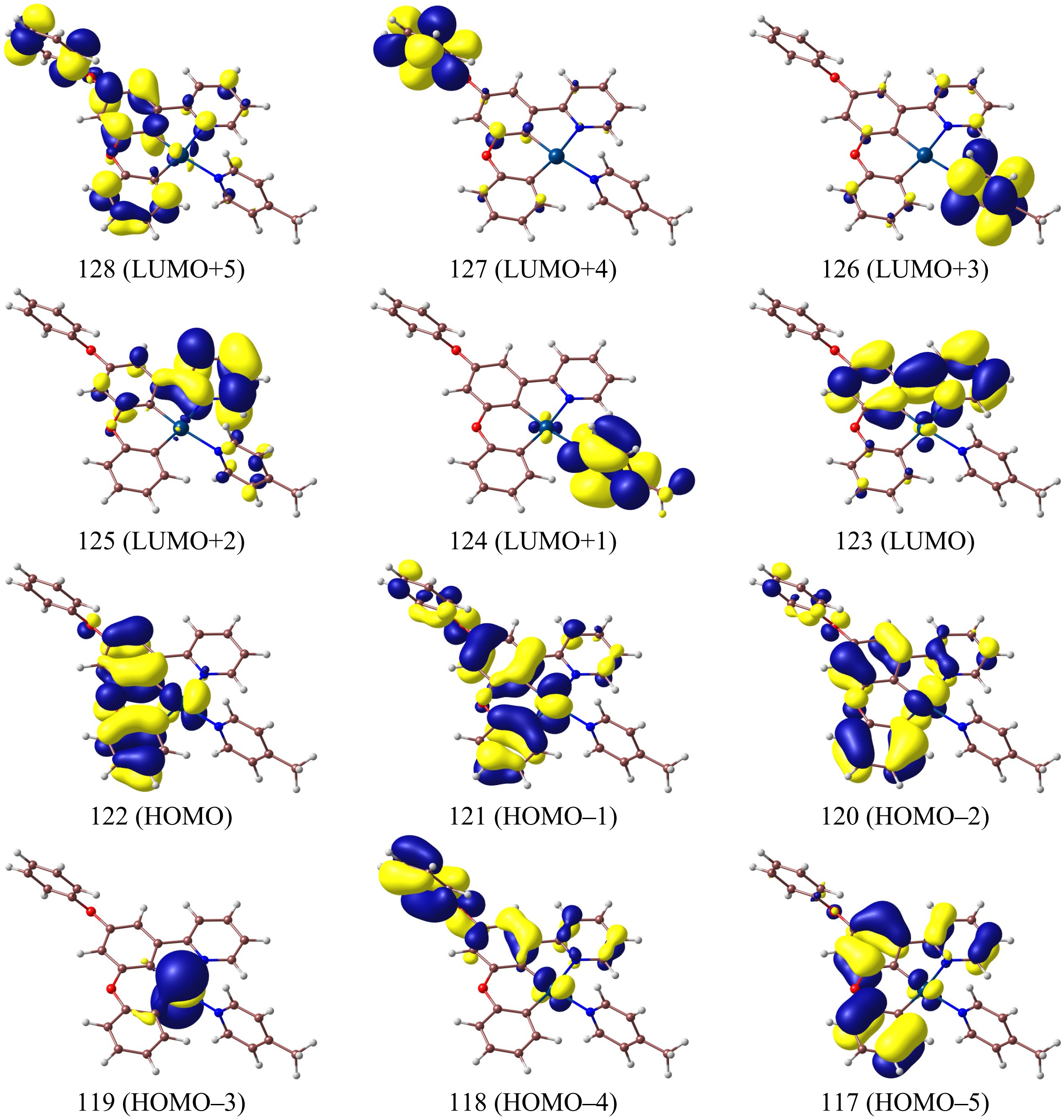


**Figure S51.** Molecular orbital isosurfaces of **3** (0.03 e $bohr^{-3}$).

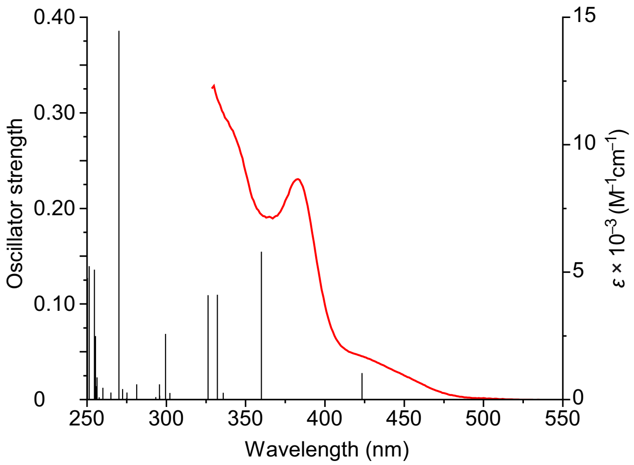


**Figure S52.** Calculated stick absorption spectrum of **3** compared with the experimental spectrum in acetone solution (*ca.* 5 × 10$^{-5}$ M) at 298 K.

**Table S11.** Selected vertical singlet excitations of **3** from TDDFT calculations at the ground state geometry in acetone solution.

| State | Monoexcitations | Coefficient (percentage) | ΔE/eV | λ/nm | Oscilator strength |
|---|---|---|---|---|---|
| S1 | 122 ->123 | 0.69389 (96%) | 2.929 | 423.4 | 0.0272 |
| S2 | 120 ->123<br>121 ->123<br>122 ->125 | 0.10695 (2%)<br>0.67247 (90%)<br>-0.15249 (5%) | 3.445 | 359.9 | 0.1541 |
| S5 | 120 ->123<br>122 ->125 | 0.61722 (76%)<br>0.29593 (18%) | 3.732 | 332.2 | 0.1090 |
| S6 | 120 ->123<br>121 ->123<br>122 ->125 | -0.28537 (16%)<br>0.18138 (7%)<br>0.60616 (73%) | 3.800 | 326.3 | 0.1086 |
| S9 | 118 ->123<br>120 ->125<br>122 ->126 | 0.62882 (79%)<br>0.10733 (2%)<br>-0.2611 (14%) | 4.141 | 299.4 | 0.0682 |
| S11 | 116 ->123<br>121 ->125 | -0.23861 (11%)<br>0.6369 (81%) | 4.194 | 295.7 | 0.0153 |
| S14 | 117 ->123<br>120 ->125 | 0.52696 (56%)<br>-0.40973 (34%) | 4.408 | 281.3 | 0.0155 |
| S17 | 117 ->123<br>120 ->125<br>120 ->126<br>121 ->126<br>122 ->127<br>122 ->128 | 0.14083 (4%)<br>0.25004 (13%)<br>0.11834 (3%)<br>0.46224 (43%)<br>-0.21533 (9%)<br>0.31935 (20%) | 4.552 | 272.4 | 0.0103 |
| S18 | 117 ->123<br>120 ->125<br>122 ->127<br>122 ->128 | 0.40088 (32%)<br>0.41034 (34%)<br>0.20285 (8%)<br>-0.28677 (16%) | 4.590 | 270.1 | 0.3853 |

**Table S12.** Lowest vertical triplet excitations of **3** from TDDFT calculations at the ground state geometry in acetone solution.

| State | Monoexcitations | Coefficient (percentage) | ΔE/eV | λ/nm |
|---|---|---|---|---|
| T1 | 118 ->123<br>120 ->123<br>121 ->123<br>122 ->123 | 0.1143 (3%)<br>0.15472 (5%)<br>-0.2248 (10%)<br>0.62157 (77%) | 2.613 | 474.4 |
| T2 | 117 ->123<br>118 ->123<br>120 ->123<br>121 ->123<br>122 ->123 | 0.19444 (8%)<br>-0.19146 (7%)<br>-0.3173 (20%)<br>0.40862 (33%)<br>0.29633 (18%) | 2.819 | 439.8 |
| T3 | 117 ->123<br>120 ->123<br>121 ->123<br>122 ->125 | -0.14029 (4%)<br>0.41749 (35%)<br>0.46688 (44%)<br>0.12655 (3%) | 3.159 | 392.5 |
| T4 | 122 ->124 | 0.69865 (98%) | 3.516 | 352.7 |
| T5 | 119 ->123 | 0.69526 (97%) | 3.556 | 348.7 |

## 4.2. Complex [Pt(dPhOppy)(CO)] (9)

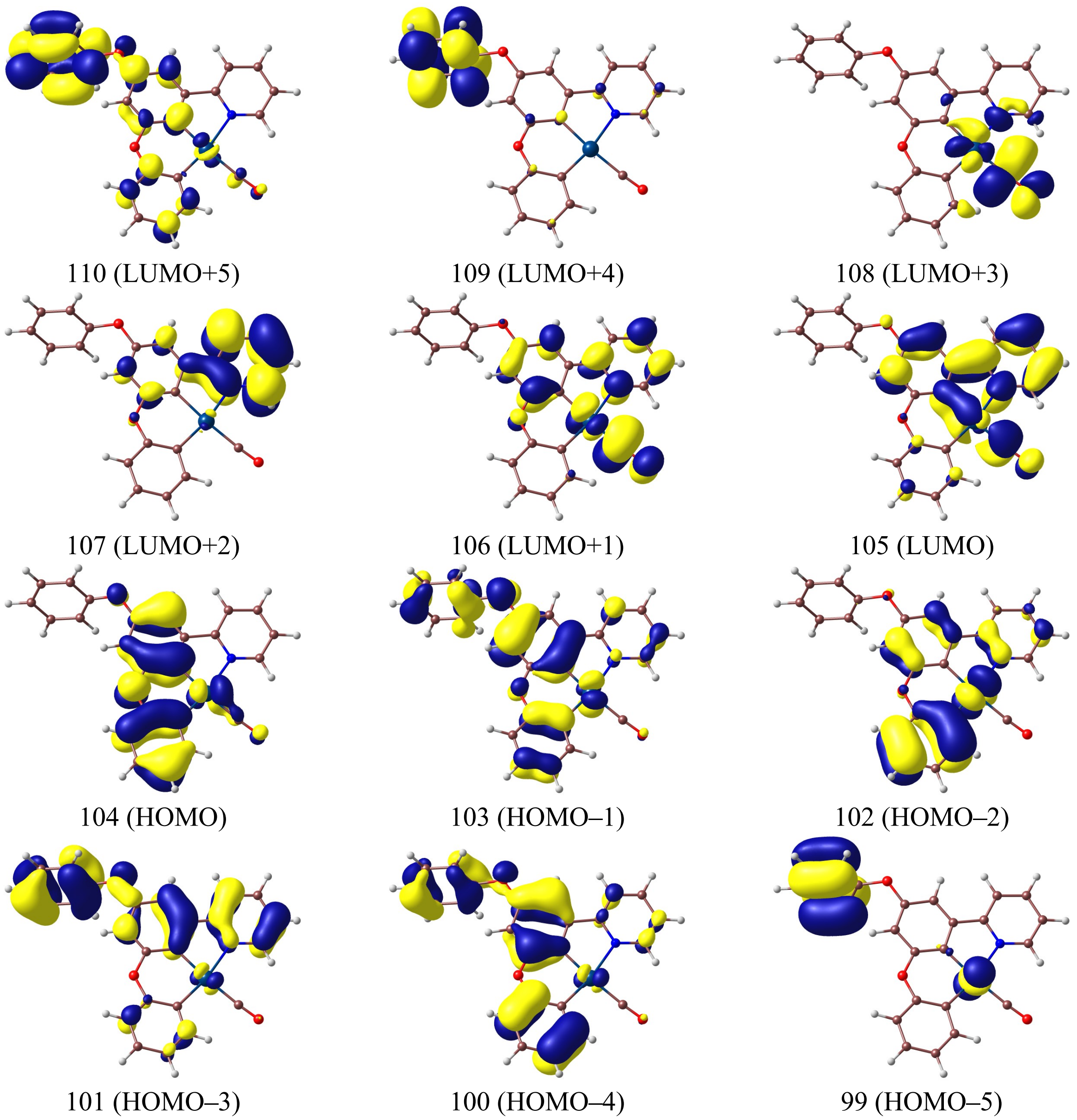


**Figure S53.** Molecular orbital isosurfaces of **9** (0.03 e bohr$^{-3}$).

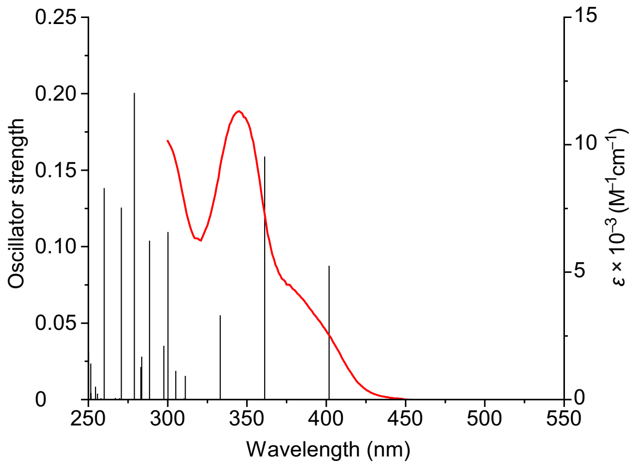


**Figure S54.** Calculated stick absorption spectrum of **9** compared with the experimental spectrum in MeCN solution (*ca.* $5 \times 10^{-5}$ M) at 298 K.

**Table S13.** Selected vertical singlet excitations of **9** from TDDFT calculations at the ground state geometry in MeCN solution.

| State | Monoexcitations | Coefficient (percentage) | ΔE/eV | λ/nm | Oscilator strength |
|---|---|---|---|---|---|
| S1 | 104 ->105 | 0.69189 (96%) | 3.086 | 401.8 | 0.0871 |
| S2 | 103 ->105 | 0.69005 (95%) | 3.434 | 361.1 | 0.1586 |
| S3 | 102 ->105 | 0.68056 (93%) | 3.721 | 333.2 | 0.0548 |
| S4 | 104 ->106<br>104 ->108 | 0.66984 (90%)<br>0.13668 (4%) | 3.985 | 311.2 | 0.0151 |
| S6 | 101 ->105<br>103 ->107<br>104 ->107 | 0.4374 (38%)<br>0.12165 (3%)<br>0.50615 (51%) | 4.063 | 305.2 | 0.0184 |
| S7 | 98 ->105<br>99 ->105<br>101 ->105<br>103 ->106<br>104 ->107 | -0.22804 (10%)<br>-0.10633 (2%)<br>-0.42741 (37%)<br>0.17183 (6%)<br>0.44689 (40%) | 4.130 | 300.2 | 0.1092 |
| S8 | 98 ->105<br>99 ->105<br>100 ->105<br>101 ->105<br>103 ->106<br>104 ->107 | 0.58594 (69%)<br>0.22254 (10%)<br>0.14285 (4%)<br>-0.19543 (8%)<br>0.1084 (2%)<br>0.12556 (3%) | 4.167 | 297.6 | 0.0348 |
| S9 | 100 ->105<br>101 ->105<br>102 ->106<br>103 ->106 | 0.34789 (24%)<br>0.22888 (10%)<br>-0.14666 (4%)<br>0.51563 (53%) | 4.296 | 288.6 | 0.1036 |
| S10 | 100 ->105<br>102 ->106<br>102 ->108<br>103 ->106 | -0.34304 (24%)<br>-0.11438 (3%)<br>0.16526 (5%)<br>0.20336 (8%) | 4.371 | 283.7 | 0.0277 |

| | | | | | |
|---|---|---|---|---|---|
| | 103 ->107<br>103 ->108 | 0.37619 (28%)<br>-0.35091 (25%) | | | |
| S11 | 100 ->105<br>102 ->108<br>103 ->106<br>103 ->107<br>103 ->108 | -0.27512 (15%)<br>-0.2218 (10%)<br>0.15876 (5%)<br>0.19803 (8%)<br>0.51937 (54%) | 4.379 | 283.2 | 0.0209 |
| S12 | 100 ->105<br>102 ->107<br>103 ->106<br>103 ->107 | 0.35983 (26%)<br>-0.10688 (2%)<br>-0.19671 (8%)<br>0.53428 (57%) | 4.443 | 279.0 | 0.2002 |
| S13 | 102 ->106<br>103 ->106 | 0.63323 (80%)<br>0.20794 (9%) | 4.578 | 270.8 | 0.1252 |
| S16 | 96 ->105<br>101 ->106<br>102 ->107<br>103 ->106 | -0.1472 (4%)<br>0.12561 (3%)<br>0.62252 (78%)<br>-0.1135 (3%) | 4.768 | 260.0 | 0.1379 |

**Table S14.** Lowest vertical triplet excitations of **9** from TDDFT calculations at the ground state geometry in MeCN solution.

| State | Monoexcitations | Coefficient (percentage) | ΔE/eV | λ/nm |
|---|---|---|---|---|
| T1 | 101 ->105<br>102 ->105<br>103 ->105<br>104 ->105 | 0.16212 (5%)<br>0.1419 (4%)<br>0.40096 (32%)<br>0.50365 (51%) | 2.720 | 455.8 |
| T2 | 100 ->105<br>101 ->105<br>102 ->105<br>103 ->105<br>104 ->105 | 0.11162 (2%)<br>-0.20784 (9%)<br>-0.15424 (5%)<br>-0.37185 (28%)<br>0.46637 (44%) | 2.762 | 448.9 |
| T3 | 100 ->105<br>101 ->105<br>102 ->105<br>103 ->105<br>104 ->106<br>104 ->107 | -0.10572 (2%)<br>0.13004 (3%)<br>0.48863 (48%)<br>-0.34353 (24%)<br>-0.13763 (4%)<br>0.10655 (2%) | 3.256 | 380.8 |
| T4 | 102 ->105<br>102 ->111<br>103 ->106<br>104 ->106<br>104 ->107<br>104 ->110<br>104 ->111<br>104 ->113 | 0.18978 (7%)<br>0.12951 (3%)<br>0.12203 (3%)<br>0.52186 (54%)<br>-0.20239 (8%)<br>0.10778 (2%)<br>-0.10363 (2%)<br>-0.13108 (3%) | 3.498 | 354.4 |

### 4.3. Complex [Pt(dPhOppy)(trz)] (11)

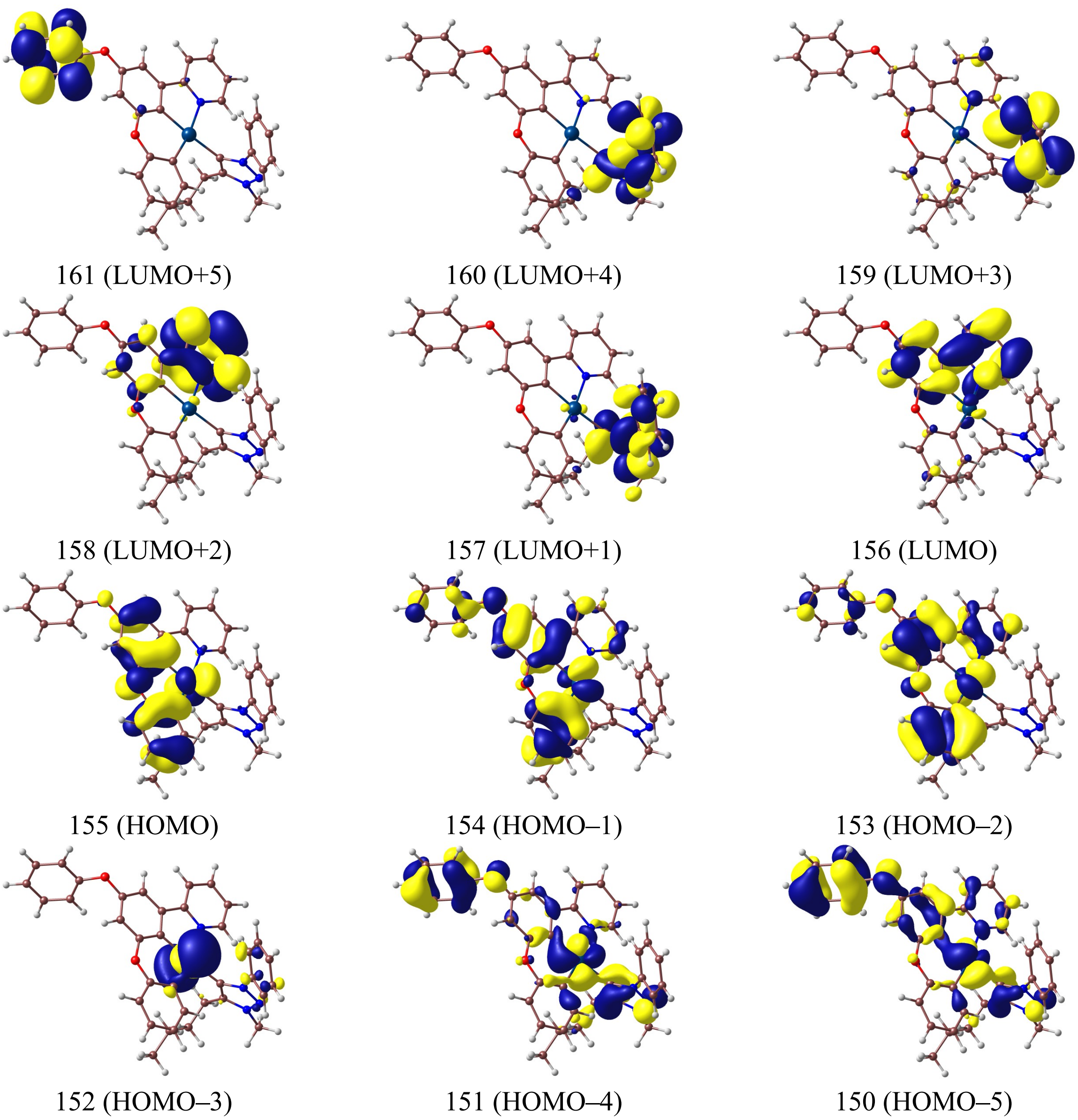


**Figure S55.** Molecular orbital isosurfaces of **11** (0.03 e bohr$^{-3}$).

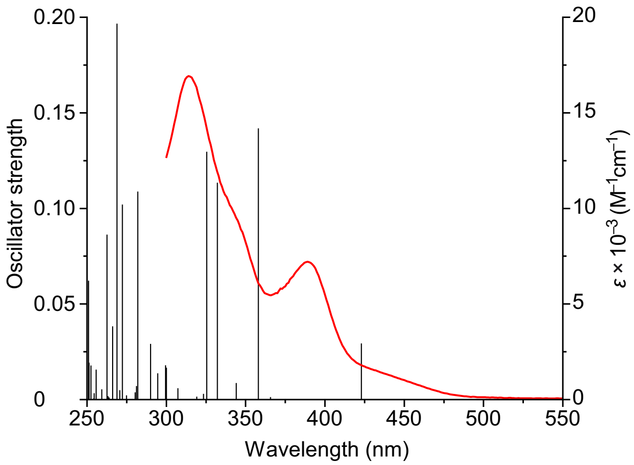


**Figure S56.** Calculated stick absorption spectrum of **11** compared with the experimental spectrum in MeCN solution (*ca.* 5 × $10^{-5}$ M) at 298 K.

**Table S15.** Selected vertical singlet excitations of **11** from TDDFT calculations at the ground state geometry in MeCN solution.

| State | Monoexcitations | Coefficient (percentage) | ΔE/eV | λ/nm | Oscilator strength |
|---|---|---|---|---|---|
| S1 | 155 ->156 | 0.69389 (96%) | 2.931 | 423.0 | 0.0291 |
| S3 | 153 ->156<br>154 ->156<br>155 ->158 | 0.10744 (2%)<br>0.66887 (89%)<br>0.16542 (5%) | 3.463 | 358.0 | 0.1416 |
| S5 | 153 ->156<br>155 ->158 | 0.62215 (77%)<br>-0.28709 (16%) | 3.732 | 332.2 | 0.1132 |
| S6 | 153 ->156<br>154 ->156<br>155 ->158 | 0.27409 (15%)<br>-0.19084 (7%)<br>0.60127 (72%) | 3.809 | 325.5 | 0.1294 |
| S10 | 150 ->156<br>151 ->156<br>153 ->157<br>154 ->157 | 0.43743 (38%)<br>-0.30867 (19%)<br>-0.40602 (33%)<br>0.11586 (3%) | 4.133 | 300.0 | 0.0164 |
| S11 | 150 ->156<br>151 ->156<br>153 ->157<br>154 ->157 | 0.34883 (24%)<br>-0.23323 (11%)<br>0.52274 (55%)<br>-0.14639 (4%) | 4.139 | 299.5 | 0.0177 |
| S12 | 154 ->158<br>155 ->159 | 0.66897 (90%)<br>0.14046 (4%) | 4.209 | 294.6 | 0.0135 |
| S13 | 154 ->158<br>155 ->159 | -0.15223 (5%)<br>0.6699 (90%) | 4.275 | 290.0 | 0.0289 |
| S14 | 146 ->157<br>149 ->156<br>150 ->157<br>151 ->157<br>153 ->158 | -0.11953 (3%)<br>-0.2173 (9%)<br>0.35333 (25%)<br>0.49047 (48%)<br>-0.21118 (9%) | 4.397 | 282.0 | 0.1085 |

| | | | | | |
|---|---|---|---|---|---|
| S18 | 146 ->156<br>147 ->156<br>149 ->156<br>153 ->158<br>154 ->159<br>155 ->160<br>155 ->161<br>155 ->162 | -0.20302 (8%)<br>-0.15533 (5%)<br>-0.34029 (23%)<br>0.40201 (32%)<br>-0.10241 (2%)<br>0.19269 (7%)<br>0.12684 (3%)<br>-0.19861 (8%) | 4.554 | 272.3 | 0.1018 |
| S20 | 146 ->156<br>147 ->156<br>149 ->156<br>153 ->158<br>155 ->161<br>155 ->162<br>155 ->163 | -0.17462 (6%)<br>-0.12346 (3%)<br>0.24794 (12%)<br>-0.28084 (16%)<br>0.38783 (30%)<br>-0.31776 (20%)<br>0.10449 (2%) | 4.611 | 268.9 | 0.1964 |

**Table S16.** Lowest vertical triplet excitations of **11** from TDDFT calculations at the ground state geometry in MeCN solution.

| State | Monoexcitations | Coefficient (percentage) | ΔE/eV | λ/nm |
|---|---|---|---|---|
| T1 | 153 ->156<br>154 ->156<br>155 ->156 | -0.15162 (5%)<br>0.20064 (8%)<br>0.63324 (80%) | 2.631 | 471.3 |
| T2 | 149 ->156<br>150 ->156<br>153 ->156<br>154 ->156<br>155 ->156 | -0.19647 (8%)<br>-0.1636 (5%)<br>-0.3508 (25%)<br>0.39456 (31%)<br>-0.26833 (14%) | 2.829 | 438.3 |
| T3 | 149 ->156<br>153 ->156<br>154 ->156<br>155 ->158 | 0.12798 (3%)<br>0.39752 (32%)<br>0.4902 (48%)<br>-0.12858 (3%) | 3.166 | 391.6 |
| T4 | 140 ->157<br>142 ->157<br>146 ->157<br>150 ->157<br>151 ->157<br>151 ->160<br>152 ->157<br>155 ->157 | -0.10342 (2%)<br>-0.11488 (3%)<br>0.20419 (8%)<br>0.284 (16%)<br>0.41849 (35%)<br>0.10766 (2%)<br>0.12575 (3%)<br>0.32587 (21%) | 3.293 | 376.5 |

### 4.4. Complex [Pt(dmtppy)(CO)] (15)

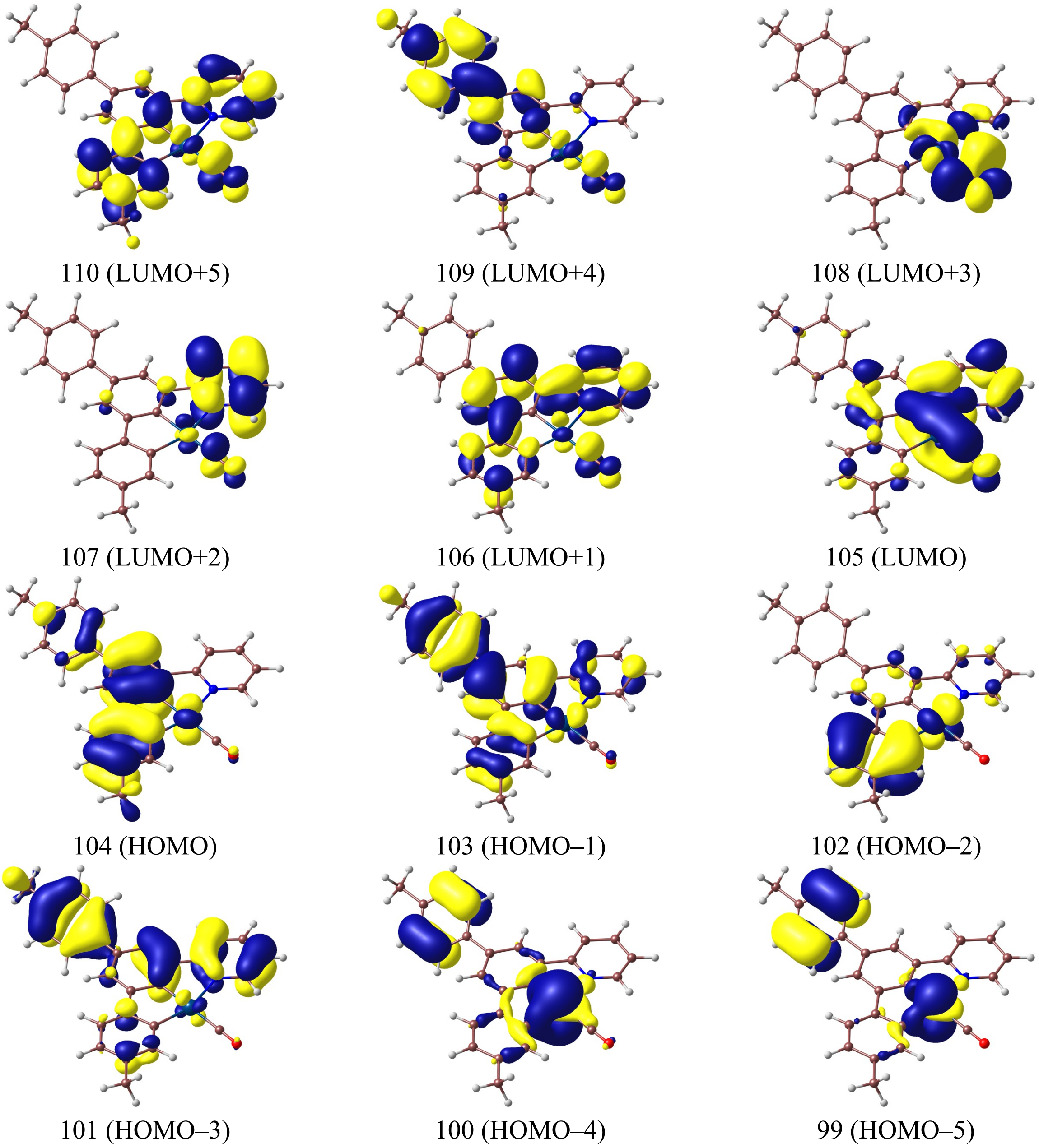


**Figure S57.** Molecular orbital isosurfaces of **15** (0.03 e bohr$^{-3}$).

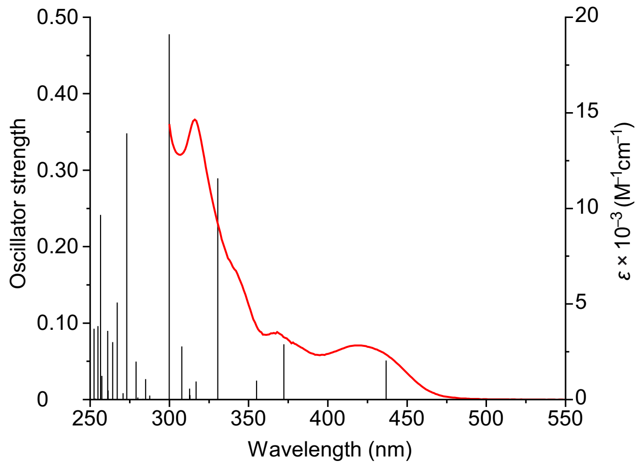


**Figure S58.** Calculated stick absorption spectrum of **15** compared with the experimental spectrum in MeCN solution (*ca.* 5 × $10^{-5}$ M) at 298 K.

**Table S17.** Selected vertical singlet excitations of **15** from TDDFT calculations at the ground state geometry in MeCN solution.

| State | Monoexcitations | Coefficient (percentage) | ΔE/eV | λ/nm | Oscilator strength |
|---|---|---|---|---|---|
| S1 | 103 ->105<br>104 ->105 | 0.10003 (2%)<br>0.69009 (95%) | 2.838 | 436.9 | 0.0504 |
| S2 | 102 ->105<br>103 ->105<br>104 ->105 | 0.11454 (3%)<br>0.67582 (91%)<br>-0.10733 (2%) | 3.331 | 372.2 | 0.0717 |
| S3 | 102 ->105<br>104 ->106 | -0.31318 (20%)<br>0.60396 (73%) | 3.493 | 355.0 | 0.0240 |
| S4 | 102 ->105<br>103 ->105<br>104 ->106 | 0.60111 (72%)<br>-0.10373 (2%)<br>0.32433 (21%) | 3.751 | 330.5 | 0.2887 |
| S5 | 99 ->105<br>100 ->105 | 0.39568 (31%)<br>0.57109 (65%) | 3.914 | 316.8 | 0.0228 |
| S7 | 103 ->106<br>104 ->107<br>104 ->108 | -0.29395 (17%)<br>0.49932 (50%)<br>0.37061 (27%) | 3.964 | 312.8 | 0.0137 |
| S8 | 101 ->105<br>102 ->106<br>103 ->106<br>104 ->107 | 0.43149 (37%)<br>0.14765 (4%)<br>0.39603 (31%)<br>0.31821 (20%) | 4.028 | 307.8 | 0.0687 |
| S9 | 101 ->105<br>103 ->106<br>104 ->107 | 0.51176 (52%)<br>-0.4097 (34%)<br>-0.17767 (6%) | 4.134 | 299.9 | 0.4770 |
| S11 | 102 ->106<br>103 ->106<br>104 ->110<br>104 ->112 | 0.62701 (79%)<br>-0.12598 (3%)<br>-0.13473 (4%)<br>0.12538 (3%) | 4.350 | 285.0 | 0.0260 |

| | | | | | |
|---|---|---|---|---|---|
| S14 | 96 ->105<br>101 ->105<br>103 ->107<br>104 ->109 | 0.11358 (3%)<br>-0.10304 (2%)<br>0.61879 (77%)<br>-0.19405 (8%) | 4.443 | 279.0 | 0.0490 |
| S15 | 102 ->106<br>103 ->107<br>104 ->109 | -0.1204 (3%)<br>0.15901 (5%)<br>0.65396 (86%) | 4.539 | 273.2 | 0.3474 |
| S17 | 96 ->105<br>97 ->105<br>101 ->106<br>102 ->106<br>103 ->107<br>104 ->110 | -0.10146 (2%)<br>-0.35676 (25%)<br>0.11789 (3%)<br>0.11537 (3%)<br>0.12436 (3%)<br>0.51175 (52%) | 4.642 | 267.1 | 0.1261 |
| S18 | 97 ->105<br>99 ->108<br>100 ->108<br>101 ->106<br>104 ->110 | -0.34338 (24%)<br>-0.20796 (9%)<br>-0.282 (16%)<br>0.38803 (30%)<br>-0.27518 (15%) | 4.692 | 264.3 | 0.0744 |
| S19 | 100 ->108<br>101 ->106<br>102 ->108<br>103 ->108 | -0.13398 (4%)<br>-0.11721 (3%)<br>0.59259 (70%)<br>-0.25182 (13%) | 4.743 | 261.4 | 0.0110 |
| S20 | 98 ->108<br>99 ->108<br>100 ->108<br>101 ->106<br>102 ->107<br>102 ->108<br>104 ->110 | 0.11832 (3%)<br>0.29324 (17%)<br>0.39225 (31%)<br>0.29997 (18%)<br>0.19025 (7%)<br>0.21443 (9%)<br>-0.11563 (3%) | 4.749 | 261.1 | 0.0890 |

**Table S18.** Lowest vertical triplet excitations of **15** from TDDFT calculations at the ground state geometry in MeCN solution.

| **State** | **Monoexcitations** | **Coefficient (percentage)** | **ΔE/eV** | **λ/nm** |
|---|---|---|---|---|
| T1 | 101 ->105<br>103 ->105<br>103 ->106<br>104 ->105<br>104 ->106<br>104 ->110 | 0.11387 (3%)<br>-0.16262 (5%)<br>-0.11967 (3%)<br>0.55927 (63%)<br>-0.27562 (15%)<br>0.11493 (3%) | 2.428 | 510.6 |
| T2 | 101 ->105<br>101 ->106<br>103 ->105<br>103 ->106<br>104 ->105<br>104 ->106 | -0.1515 (5%)<br>-0.12784 (3%)<br>0.42377 (36%)<br>0.16796 (6%)<br>0.37581 (28%)<br>0.25342 (13%) | 2.772 | 447.2 |

| | | | | |
|---|---|---|---|---|
| T3 | 101 ->105<br>103 ->105<br>104 ->105<br>104 ->106<br>104 ->110 | 0.10287 (2%)<br>-0.39592 (31%)<br>0.15184 (5%)<br>0.49096 (48%)<br>-0.11872 (3%) | 2.871 | 431.9 |
| T4 | 99 ->111<br>101 ->105<br>101 ->109<br>102 ->105<br>103 ->105<br>103 ->106<br>103 ->109<br>104 ->106<br>104 ->109 | -0.11797 (3%)<br>-0.19993 (8%)<br>-0.11738 (3%)<br>-0.2482 (12%)<br>-0.10836 (2%)<br>0.43644 (38%)<br>-0.15987 (5%)<br>-0.1843 (7%)<br>0.2125 (9%) | 3.318 | 373.7 |

### 4.5. Complex [Pt(dmtppy)(trz)] (17)

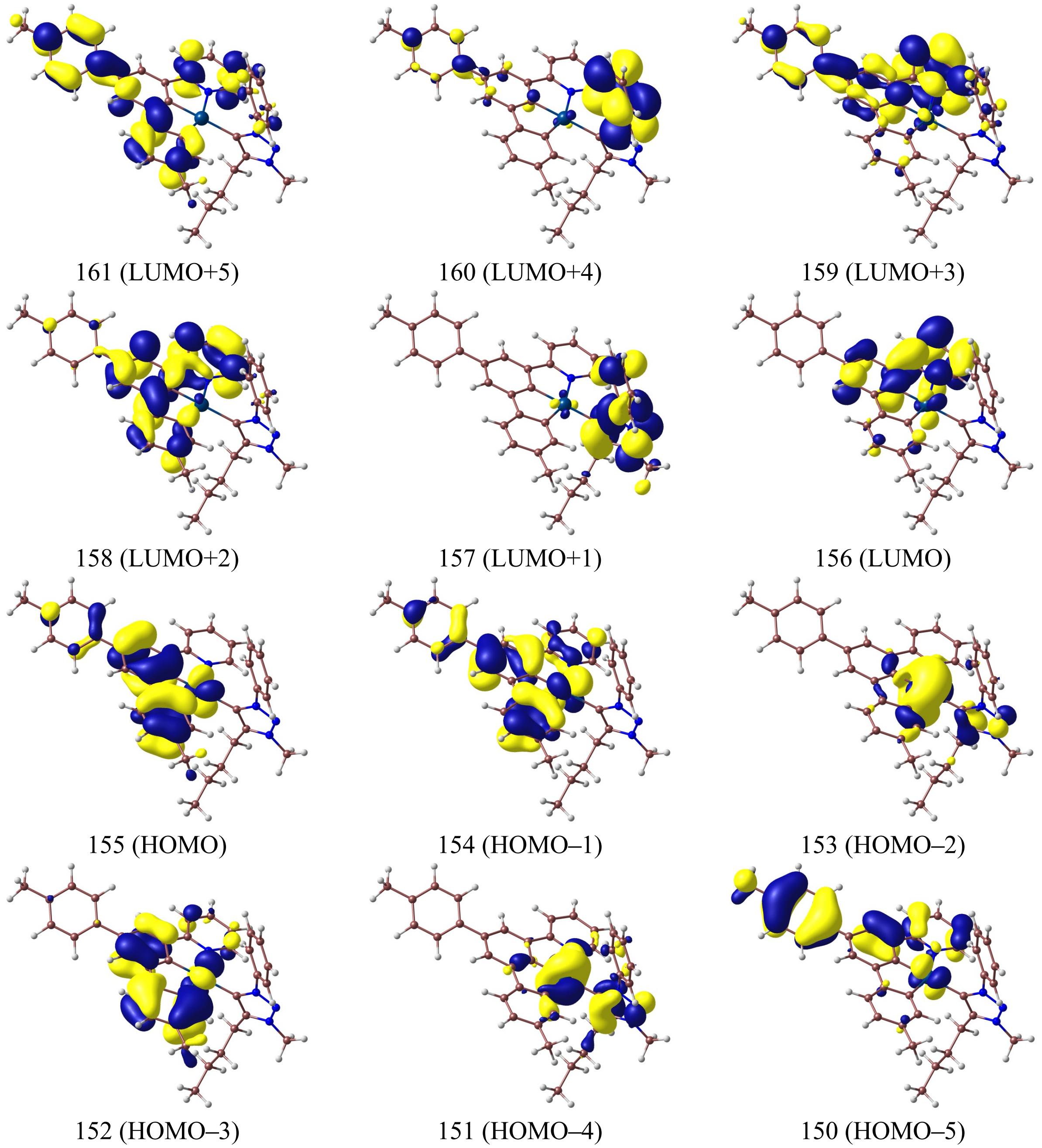


**Figure S59.** Molecular orbital isosurfaces of **17** (0.03 e $bohr^{-3}$).

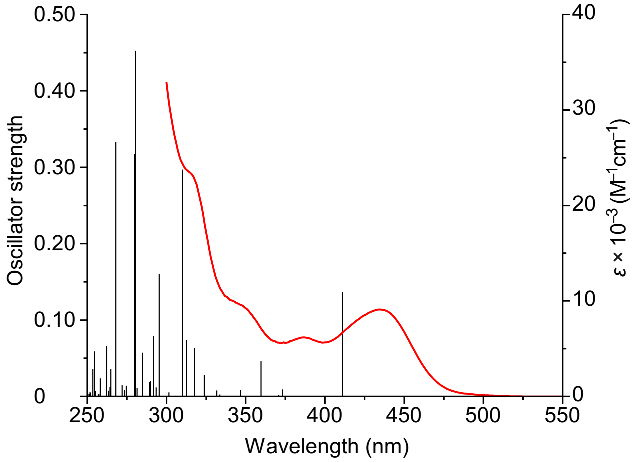


**Figure S60.** Calculated stick absorption spectrum of **17** compared with the experimental spectrum in MeCN solution (*ca.* 5 × $10^{-5}$ M) at 298 K.

**Table S19.** Selected vertical singlet excitations of **17** from TDDFT calculations at the ground state geometry in MeCN solution.

| State | Monoexcitations | Coefficient (percentage) | ΔE/eV | λ/nm | Oscilator strength |
|---|---|---|---|---|---|
| S1 | 155 ->156 | 0.69473 (97%) | 3.016 | 411.1 | 0.1357 |
| S4 | 154 ->156<br>155 ->158 | 0.21399 (9%)<br>0.64993 (84%) | 3.448 | 359.6 | 0.0454 |
| S8 | 152 ->156<br>154 ->158<br>155 ->159 | 0.26024 (14%)<br>0.56075 (63%)<br>-0.29534 (17%) | 3.828 | 323.9 | 0.0271 |
| S9 | 152 ->156<br>154 ->158<br>155 ->159 | 0.37703 (28%)<br>0.10476 (2%)<br>0.55761 (62%) | 3.903 | 317.7 | 0.0629 |
| S10 | 151 ->157<br>152 ->156<br>153 ->157 | -0.22041 (10%)<br>-0.15908 (5%)<br>0.63455 (81%) | 3.965 | 312.7 | 0.0731 |
| S11 | 152 ->156<br>152 ->158<br>153 ->157<br>154 ->158<br>155 ->159 | 0.45827 (42%)<br>-0.11499 (3%)<br>0.17937 (6%)<br>-0.34212 (23%)<br>-0.27572 (15%) | 3.998 | 310.2 | 0.2963 |
| S13 | 151 ->157<br>152 ->157<br>153 ->157<br>153 ->158<br>155 ->160 | 0.5875 (69%)<br>-0.22681 (10%)<br>0.2014 (8%)<br>0.11612 (3%)<br>0.14069 (4%) | 4.197 | 295.4 | 0.1595 |
| S14 | 151 ->157<br>152 ->157<br>154 ->157 | 0.21977 (10%)<br>0.64069 (82%)<br>-0.11164 (2%) | 4.224 | 293.5 | 0.0111 |
| S15 | 152 ->157<br>152 ->158 | 0.10191 (2%)<br>0.28746 (17%) | 4.249 | 291.8 | 0.0780 |

| | | | | | |
|---|---|---|---|---|---|
| | 155 ->161 | 0.58199 (68%) | | | |
| S16 | 151 ->157<br>154 ->159<br>155 ->160 | -0.12295 (3%)<br>0.13473 (4%)<br>0.65932 (87%) | 4.276 | 290.0 | 0.0193 |
| S17 | 151 ->158<br>155 ->161 | 0.67478 (91%)<br>0.11798 (3%) | 4.288 | 289.2 | 0.0184 |
| S18 | 150 ->156<br>152 ->156<br>152 ->158<br>154 ->159<br>154 ->161<br>155 ->160<br>155 ->161<br>155 ->164 | 0.31191 (19%)<br>-0.11625 (3%)<br>-0.13829 (4%)<br>0.53248 (57%)<br>-0.11632 (3%)<br>-0.12097 (3%)<br>0.11277 (3%)<br>0.10145 (2%) | 4.352 | 284.9 | 0.0567 |
| S19 | 146 ->156<br>147 ->156<br>149 ->156<br>150 ->156<br>154 ->159 | -0.19001 (7%)<br>0.32144 (21%)<br>0.52539 (55%)<br>0.21136 (9%)<br>-0.12772 (3%) | 4.405 | 281.4 | 0.0102 |
| S20 | 147 ->156<br>149 ->156<br>150 ->156<br>152 ->158<br>154 ->158<br>155 ->161<br>155 ->164 | -0.10054 (2%)<br>-0.10745 (2%)<br>0.34343 (24%)<br>0.46105 (43%)<br>-0.12717 (3%)<br>-0.23389 (11%)<br>-0.15287 (5%) | 4.422 | 280.4 | 0.4519 |

**Table S20.** Lowest vertical triplet excitations of **17** from TDDFT calculations at the ground state geometry in MeCN solution.

| **State** | **Monoexcitations** | **Coefficient (percentage)** | **ΔE/eV** | **λ/nm** |
|---|---|---|---|---|
| T1 | 150 ->156<br>152 ->156<br>152 ->158<br>154 ->156<br>155 ->156<br>155 ->158<br>155 ->159 | 0.1019 (2%)<br>0.13573 (4%)<br>0.10203 (2%)<br>-0.28308 (16%)<br>0.3702 (27%)<br>-0.36621 (27%)<br>0.20972 (9%) | 2.577 | 481.2 |
| T2 | 155 ->156<br>155 ->158 | 0.56239 (63%)<br>0.35231 (25%) | 2.676 | 463.4 |
| T3 | 148 ->156<br>150 ->156<br>154 ->156<br>155 ->156<br>155 ->158 | 0.1159 (3%)<br>-0.10283 (2%)<br>0.54865 (60%)<br>0.1535 (5%)<br>-0.29261 (17%) | 2.884 | 430.0 |

| | | | | |
|---|---|---|---|---|
| T4 | 143 ->157<br>144 ->157<br>146 ->157<br>151 ->157<br>151 ->162<br>153 ->157<br>155 ->157 | -0.2038 (8%)<br>0.10425 (2%)<br>-0.11234 (3%)<br>0.39112 (31%)<br>0.10131 (2%)<br>0.36854 (27%)<br>0.27047 (15%) | 3.202 | 387.2 |

**Table S21.** Energies, free energies, enthalpies and entropies of the optimized structures.[a]

| Complex | $E_0$ [b] | ZPE [c] | G [d] | H [e] | S [f] |
|---|---|---|---|---|---|
| **3** | -1497.559550 | -1497.123095 | -1497.187722 | -1497.093701 | 197.883 |
| **3-T1** | -1497.471490 | -1497.038269 | -1497.103527 | -1497.008414 | 200.181 |
| **9** | -1323.256676 | -1322.929420 | -1322.985155 | -1322.905388 | 167.884 |
| **9-T1** | -1323.162649 | -1322.839450 | -1322.896067 | -1322.814868 | 170.897 |
| **11** | -1879.790600 | -1879.189542 | -1879.266054 | -1879.150761 | 242.655 |
| **11-T1** | -1879.702490 | -1879.104704 | -1879.182494 | -1879.065424 | 246.396 |
| **15** | -1251.476071 | -1251.102430 | -1251.160224 | -1251.076442 | 176.333 |
| **15-T1** | -1251.391629 | -1251.021451 | -1251.079467 | -1250.995235 | 177.282 |
| **17** | -1808.008186 | -1807.360547 | -1807.438510 | -1807.319882 | 249.673 |
| **17-T1** | -1807.917997 | -1807.274397 | -1807.354093 | -1807.233211 | 254.417 |

[a] Thermal corrections from vibrational calculations at 298.15 K. [b] Electronic energy (Hartrees). [c] Sum of electronic and zero-point energies (Hartrees). [d] Free Energy (Hartrees). [e] Enthalpy (Hartrees). [f] Entropy (cal $mol^{-1}$ $K^{-1}$).

**Table S22.** Cartesian coordinates (Å) of the optimized structures.

**3**

| | | | |
|---|---|---|---|
| C | 4.890367384 | -0.533539342 | 8.155908842 |
| H | 4.966665150 | -1.352842346 | 8.876778486 |
| H | 5.529496902 | -0.790193731 | 7.301572124 |
| H | 3.860757847 | -0.474992595 | 7.793114022 |
| C | 7.794011666 | 6.855988264 | 11.130729496 |
| C | 7.479195918 | 7.654024545 | 12.234846184 |
| C | 8.132779352 | 8.869365083 | 12.486122614 |
| C | 9.139187059 | 9.291422569 | 11.621957963 |
| C | 9.493006345 | 8.532555036 | 10.509315753 |
| C | 8.818720492 | 7.327897845 | 10.265159508 |
| C | 9.133514438 | 6.474638585 | 9.104629774 |
| C | 10.104028635 | 6.758759194 | 8.135676756 |
| C | 10.312278615 | 5.873328179 | 7.082941062 |
| C | 9.545839455 | 4.709882011 | 7.009013783 |
| C | 8.596473191 | 4.481021133 | 7.998206032 |
| C | 5.722154463 | 5.183488770 | 12.233097676 |
| C | 4.784745960 | 4.151621107 | 12.489315881 |
| C | 3.912209000 | 4.151962362 | 13.576982301 |
| C | 3.938002067 | 5.216196491 | 14.482881786 |
| C | 4.839428470 | 6.252747209 | 14.277438456 |
| C | 5.711599441 | 6.233861508 | 13.175464988 |
| C | 10.385618401 | 10.838090138 | 12.971794825 |
| C | 5.186282133 | 3.171939909 | 8.978013110 |
| C | 4.733608335 | 1.986012942 | 8.412891126 |
| C | 5.333027678 | 0.768965188 | 8.762723114 |
| C | 6.381567260 | 0.824748937 | 9.691537456 |
| C | 6.781133655 | 2.046982390 | 10.215681806 |
| H | 7.848796910 | 9.464792308 | 13.347406213 |
| H | 10.278661995 | 8.899383033 | 9.858825307 |
| H | 10.691948368 | 7.666258171 | 8.204394285 |
| H | 11.064029614 | 6.089971863 | 6.330171661 |
| H | 9.674309032 | 3.991907650 | 6.206606551 |
| H | 7.976115792 | 3.592663561 | 7.985003218 |
| H | 4.737764440 | 3.311737140 | 11.805932413 |
| H | 3.216996939 | 3.327608335 | 13.714402806 |
| H | 3.267556787 | 5.239812624 | 15.337704024 |
| H | 4.890077782 | 7.096606853 | 14.959663661 |
| H | 4.732738891 | 4.123797528 | 8.723127374 |
| H | 3.912031868 | 2.015818845 | 7.703978242 |
| H | 6.887657353 | -0.080693430 | 10.011872311 |
| H | 7.587975445 | 2.111265577 | 10.937879632 |
| N | 8.392559342 | 5.333577387 | 9.016274424 |
| N | 6.196366495 | 3.212759165 | 9.870269559 |
| O | 6.520677376 | 7.354209173 | 13.161783216 |
| O | 9.756929847 | 10.523011019 | 11.784231965 |
| Pt | 6.958214249 | 5.132160307 | 10.636255162 |
| H | 10.090873923 | 12.926584461 | 12.567119224 |
| C | 11.733678551 | 11.656907897 | 15.279379801 |
| C | 11.596520836 | 10.298090685 | 14.985895929 |
| C | 10.927857385 | 9.878272359 | 13.833189498 |
| H | 12.255590885 | 11.973406534 | 16.177553775 |
| H | 12.016939565 | 9.551374913 | 15.654065499 |
| H | 10.833085021 | 8.821744370 | 13.607103175 |
| C | 10.517843497 | 12.202081698 | 13.253824127 |
| C | 11.193973268 | 12.605352545 | 14.405121041 |
| H | 11.293500502 | 13.665724267 | 14.620386906 |

**3-T1**

<$S^2$> = 2.009755

| | | | |
|---|---|---|---|
| C | 4.497809112 | -0.327153789 | 8.078039458 |
| H | 4.564460881 | -1.177103476 | 8.763251974 |
| H | 5.057049127 | -0.592704279 | 7.171928778 |
| H | 3.452816932 | -0.183272606 | 7.790696106 |
| C | 8.095776050 | 6.679196760 | 11.133357390 |
| C | 7.912064813 | 7.453066998 | 12.311117453 |
| C | 8.666302945 | 8.593059152 | 12.594043710 |
| C | 9.652428347 | 8.979475068 | 11.659962732 |
| C | 9.890478732 | 8.273299407 | 10.476417015 |
| C | 9.140891327 | 7.122390961 | 10.178170442 |
| C | 9.303480137 | 6.322233050 | 9.019201251 |
| C | 10.241180053 | 6.552110610 | 7.970555568 |

| | | | |
|---|---|---|---|
| C | 10.309578435 | 5.705455058 | 6.892582458 |
| C | 9.428048674 | 4.584845619 | 6.831058988 |
| C | 8.533023516 | 4.393931660 | 7.863754475 |
| C | 5.961843922 | 5.136789550 | 12.264527101 |
| C | 4.947172223 | 4.180154307 | 12.512250423 |
| C | 4.131380341 | 4.199454417 | 13.643349647 |
| C | 4.294159306 | 5.200763904 | 14.606780436 |
| C | 5.274119714 | 6.163729012 | 14.410676556 |
| C | 6.082240901 | 6.122066781 | 13.262026003 |
| C | 10.521342152 | 10.733204350 | 13.057986951 |
| C | 5.137988052 | 3.317928191 | 8.966181818 |
| C | 4.563684070 | 2.188320770 | 8.397292357 |
| C | 5.073121494 | 0.917243532 | 8.694923496 |
| C | 6.160212523 | 0.861213398 | 9.578322153 |
| C | 6.683860399 | 2.031939050 | 10.110268957 |
| H | 8.475264508 | 9.160858296 | 13.494200405 |
| H | 10.668269486 | 8.639341258 | 9.815756259 |
| H | 10.903647896 | 7.409417971 | 8.034399427 |
| H | 11.025831007 | 5.885132060 | 6.096265051 |
| H | 9.449115326 | 3.891403698 | 5.997737327 |
| H | 7.846629982 | 3.554257935 | 7.852716791 |
| H | 4.787701653 | 3.387479428 | 11.791078831 |
| H | 3.370535071 | 3.433833502 | 13.771950832 |
| H | 3.668282175 | 5.230892859 | 15.493978586 |
| H | 5.436170885 | 6.960550672 | 15.130494830 |
| H | 4.758228978 | 4.310940665 | 8.750740425 |
| H | 3.719473777 | 2.303719595 | 7.724882437 |
| H | 6.600721838 | -0.091117263 | 9.856359235 |
| H | 7.523286490 | 2.011458509 | 10.797011960 |
| N | 8.444438398 | 5.211976720 | 8.936059303 |
| N | 6.184121069 | 3.248953894 | 9.813671459 |
| O | 6.991893470 | 7.163900418 | 13.255382554 |
| O | 10.412962154 | 10.097676338 | 11.824032921 |
| Pt | 7.130708887 | 5.081603401 | 10.604260682 |
| H | 9.778196845 | 12.573730606 | 12.236982208 |
| C | 10.885308042 | 12.123394153 | 15.436421141 |
| C | 11.227032082 | 10.770193469 | 15.356313601 |
| C | 11.052121485 | 10.065358584 | 14.163939249 |
| H | 11.027597613 | 12.666031713 | 16.366262774 |
| H | 11.639315611 | 10.259272835 | 16.221637838 |
| H | 11.327680695 | 9.018544066 | 14.086670957 |
| C | 10.179945726 | 12.083610713 | 13.118103762 |
| C | 10.365431136 | 12.777078024 | 14.316102950 |
| H | 10.100544532 | 13.828986508 | 14.371078381 |

**9**

| | | | |
|---|---|---|---|
| C | -6.309696312 | 0.667488000 | 1.582206266 |
| C | -7.364022765 | 0.878155166 | 0.688133368 |
| C | -7.283590416 | 0.366150974 | -0.609305633 |
| H | -8.242043764 | 1.434808722 | 1.002016759 |
| H | -8.098369178 | 0.523180528 | -1.310368522 |
| H | -6.368205482 | 1.055660774 | 2.595156811 |
| C | -0.015746726 | -0.225173814 | -0.156087479 |
| C | -1.012555170 | 0.743403286 | -0.245508008 |
| C | -2.374826053 | 0.417801544 | -0.359700943 |
| C | -2.748486967 | -0.920861289 | -0.372058692 |
| C | -1.785810308 | -1.931719282 | -0.284211021 |
| C | -0.437510658 | -1.583169644 | -0.180576920 |
| C | 0.623832940 | -2.601135318 | -0.077426743 |
| C | 0.413198775 | -3.984478441 | -0.100093010 |
| C | 1.495402713 | -4.852620499 | -0.000145070 |
| C | 2.781503568 | -4.326863466 | 0.121254759 |
| C | 2.931620692 | -2.946118355 | 0.139334082 |
| C | 1.701413803 | 2.094081329 | -0.054734974 |
| C | 2.802537882 | 2.986441100 | -0.013994771 |
| C | 2.676334548 | 4.370962150 | -0.081013791 |
| C | 1.405102431 | 4.942116824 | -0.194308494 |
| C | 0.295847724 | 4.111101237 | -0.240012095 |
| C | 0.440499936 | 2.713487527 | -0.173372251 |
| C | -5.109024487 | -0.548217306 | -0.115239234 |
| C | -5.178653635 | -0.050439540 | 1.189579068 |
| C | -6.153768838 | -0.346249244 | -1.018194453 |
| C | 3.890537694 | 0.179000757 | 0.286079551 |
| H | -3.107507700 | 1.212463079 | -0.437828713 |
| H | -2.119164251 | -2.962904132 | -0.297924257 |
| H | -0.591220080 | -4.377704921 | -0.197900789 |
| H | 1.335025266 | -5.926026825 | -0.018079851 |
| H | 3.654859250 | -4.964211143 | 0.200083574 |
| H | 3.913176479 | -2.497278065 | 0.230809344 |
| H | 3.806164938 | 2.583633468 | 0.071475692 |
| H | 3.563558299 | 4.996826692 | -0.044743143 |
| H | 1.279594452 | 6.019790154 | -0.247146301 |
| H | -0.707389647 | 4.516802416 | -0.329643079 |
| H | -4.363002425 | -0.225635076 | 1.883848389 |
| H | -6.073541651 | -0.749556628 | -2.022836423 |
| N | 1.886267497 | -2.104910254 | 0.045036103 |
| O | -0.786222166 | 2.088753890 | -0.236177791 |
| O | -4.050733544 | -1.339756724 | -0.536755581 |
| O | 5.023730587 | 0.148626257 | 0.470815572 |
| Pt | 1.965578868 | 0.076854783 | 0.033860886 |

**9-T$_1$**

$\langle S^2 \rangle = 2.01142$

| | | | |
|---|---|---|---|
| C | 6.191564317 | 18.303145540 | -0.256383496 |
| C | 5.247955497 | 19.057989104 | -0.958717005 |
| C | 4.000298851 | 19.322235827 | -0.386001097 |
| H | 5.485990330 | 19.443348095 | -1.945548964 |
| H | 3.264691642 | 19.911124484 | -0.925772144 |
| H | 7.166164397 | 18.104512457 | -0.692372951 |
| C | 4.535598077 | 13.748325290 | 4.280393231 |
| C | 4.547917403 | 14.017701646 | 2.878118448 |
| C | 4.493395435 | 15.300008824 | 2.360310297 |
| C | 4.425382532 | 16.390445698 | 3.274497931 |
| C | 4.405136967 | 16.207829169 | 4.659733111 |
| C | 4.471091540 | 14.915703954 | 5.195356258 |
| C | 4.489624068 | 14.615205996 | 6.582929893 |
| C | 4.433895179 | 15.570651977 | 7.635636747 |
| C | 4.452585111 | 15.166721669 | 8.948293987 |
| C | 4.529411021 | 13.778719961 | 9.243547851 |
| C | 4.584450493 | 12.881841519 | 8.197368557 |
| C | 4.595106424 | 11.012499868 | 3.378194192 |
| C | 4.555155558 | 9.600673330 | 3.287756980 |
| C | 4.517641754 | 8.905944090 | 2.078917200 |
| C | 4.518806595 | 9.610164865 | 0.872350537 |

| | | | |
|---|---|---|---|
| C | 4.551719107 | 10.997744501 | 0.908223145 |
| C | 4.584611246 | 11.674317882 | 2.138179760 |
| C | 4.639879349 | 18.070483290 | 1.562236461 |
| C | 5.897147135 | 17.806115492 | 1.014698317 |
| C | 3.688765626 | 18.830276831 | 0.882468178 |
| C | 4.866502681 | 10.333563976 | 6.207211453 |
| H | 4.476351694 | 15.456159558 | 1.290384619 |
| H | 4.353242655 | 17.090449459 | 5.286673005 |
| H | 4.375151630 | 16.624869632 | 7.387866434 |
| H | 4.409065775 | 15.897671928 | 9.749735629 |
| H | 4.545314766 | 13.415389349 | 10.264721508 |
| H | 4.642271484 | 11.816594805 | 8.390262978 |
| H | 4.550298976 | 9.010422359 | 4.197932884 |
| H | 4.488602396 | 7.819895313 | 2.083881808 |
| H | 4.492180878 | 9.089381849 | -0.080117409 |
| H | 4.549065998 | 11.586227386 | -0.004152993 |
| H | 6.629977296 | 17.235487842 | 1.575589529 |
| H | 2.726854359 | 19.024459111 | 1.345857117 |
| N | 4.568388910 | 13.256064028 | 6.895960673 |
| O | 4.608756541 | 13.045200653 | 1.944905268 |
| O | 4.321284900 | 17.675241544 | 2.860789851 |
| O | 5.053306177 | 9.408977284 | 6.865183513 |
| Pt | 4.631690919 | 11.986443186 | 5.176463974 |

**11**

| | | | |
|---|---|---|---|
| C | 7.020646263 | 18.318337108 | 0.439537749 |
| C | 6.569695435 | 19.388748221 | -0.336147684 |
| C | 5.260996875 | 19.852902340 | -0.168826278 |
| H | 7.228880844 | 19.856069995 | -1.061583776 |
| H | 4.896204977 | 20.684856342 | -0.764951225 |
| H | 8.036865922 | 17.951521017 | 0.323383576 |
| C | 4.342490091 | 13.800719606 | 4.081950048 |
| C | 4.200783626 | 14.002465310 | 2.707748134 |
| C | 4.100574515 | 15.279511937 | 2.136814689 |
| C | 4.159651001 | 16.394886024 | 2.968432881 |
| C | 4.300315807 | 16.256953752 | 4.346448850 |
| C | 4.382045540 | 14.966614298 | 4.893301932 |
| C | 4.515511276 | 14.741320048 | 6.344979840 |
| C | 4.561575367 | 15.755370398 | 7.310874901 |
| C | 4.684940993 | 15.429207185 | 8.657338735 |
| C | 4.760688573 | 14.086164958 | 9.027115785 |
| C | 4.709347823 | 13.122789203 | 8.026607292 |
| C | 4.388077870 | 11.010269809 | 3.300116412 |
| C | 4.468361017 | 9.594725113 | 3.239507498 |
| C | 4.398477626 | 8.855934829 | 2.058926137 |
| C | 4.238837238 | 9.519761494 | 0.839685764 |
| C | 4.157225872 | 10.905775771 | 0.838997352 |
| C | 4.232686034 | 11.634864331 | 2.039839047 |
| C | 4.872963994 | 18.178287385 | 1.528023204 |
| C | 6.181915934 | 17.708242995 | 1.376256243 |
| C | 4.409940044 | 19.251337136 | 0.757692067 |
| H | 3.979447780 | 15.389257424 | 1.064071798 |
| H | 4.335775334 | 17.152465435 | 4.956703293 |
| H | 4.499173378 | 16.794018758 | 7.009589263 |
| H | 4.720939106 | 16.213911533 | 9.406920108 |
| H | 4.856884176 | 13.783609294 | 10.063951343 |
| H | 4.765394372 | 12.066880668 | 8.263033527 |
| H | 4.593895503 | 9.047200231 | 4.166318005 |
| H | 4.467921789 | 7.771632495 | 2.093165540 |
| H | 4.180124694 | 8.969787314 | -0.095617975 |
| H | 4.035490381 | 11.459357405 | -0.087749895 |
| H | 6.542099952 | 16.882620228 | 1.980122342 |
| H | 3.390679648 | 19.598692363 | 0.896554532 |
| N | 4.591034850 | 13.432372182 | 6.723290084 |
| O | 4.133700122 | 12.990629461 | 1.787985481 |
| O | 3.985229915 | 17.674705434 | 2.455788108 |
| Pt | 4.494065696 | 12.034209635 | 5.047965968 |
| C | 4.728777042 | 10.325210913 | 6.252225069 |
| C | 5.903966537 | 9.776619421 | 6.779249308 |
| N | 3.766759616 | 9.470824664 | 6.754287488 |
| C | 7.327400577 | 10.203063945 | 6.598755362 |
| N | 5.541873723 | 8.679521634 | 7.508939595 |
| C | 2.346631674 | 9.498496612 | 6.542698187 |
| N | 4.240809630 | 8.475835225 | 7.512719773 |
| C | 8.062892288 | 9.455495722 | 5.466562328 |
| H | 7.321155810 | 11.275357501 | 6.379203902 |
| H | 7.874243404 | 10.071560594 | 7.540303613 |
| C | 6.396439205 | 7.765910131 | 8.265986918 |
| C | 1.647243875 | 10.699717802 | 6.676571088 |
| C | 1.687779408 | 8.308113819 | 6.224494964 |
| C | 9.510199314 | 9.930981377 | 5.295217487 |
| H | 7.512998120 | 9.599926555 | 4.528122506 |
| H | 8.052618418 | 8.377194311 | 5.672733700 |
| H | 7.086967095 | 7.264594170 | 7.585856567 |
| H | 6.956750809 | 8.329992547 | 9.013825815 |
| H | 5.753327290 | 7.034928228 | 8.753328260 |
| C | 0.265995329 | 10.705263526 | 6.474975141 |
| C | 0.305709670 | 8.326686035 | 6.033476159 |
| H | 2.252633242 | 7.387952795 | 6.121810606 |
| C | 10.248221127 | 9.200798161 | 4.169838028 |
| H | 9.513022369 | 11.011212327 | 5.095728120 |
| H | 10.051566849 | 9.791465700 | 6.240930628 |
| C | -0.406138849 | 9.522983760 | 6.154269228 |
| H | -0.283971977 | 11.635638306 | 6.579516700 |
| H | -0.211273907 | 7.405487084 | 5.782274926 |
| H | 10.290620501 | 8.121152110 | 4.357254427 |
| H | 9.748421841 | 9.350065251 | 3.205373010 |
| H | 11.278056622 | 9.561223060 | 4.069619663 |
| H | -1.481032010 | 9.533700869 | 6.000086080 |
| H | 2.174591160 | 11.608030758 | 6.942785292 |

**11-T$_1$**

<S$^2$> = 2.009644

| | | | |
|---|---|---|---|
| C | 6.407105937 | 18.398717068 | -0.371636774 |
| C | 5.415950705 | 19.115742821 | -1.048497905 |
| C | 4.194517405 | 19.375911334 | -0.421442396 |
| H | 5.596691459 | 19.473080130 | -2.057909519 |
| H | 3.421523088 | 19.934838346 | -0.940773658 |
| H | 7.361180396 | 18.200641473 | -0.851540874 |
| C | 4.693845422 | 13.884444548 | 4.258163611 |
| C | 4.662506635 | 14.167296297 | 2.868391747 |
| C | 4.678555750 | 15.462355165 | 2.341757571 |
| C | 4.734018564 | 16.533582640 | 3.255440930 |
| C | 4.762627468 | 16.343421261 | 4.643147511 |

| | | | |
|---|---|---|---|
| C | 4.743157449 | 15.043248795 | 5.177447942 |
| C | 4.767822560 | 14.734908642 | 6.562955463 |
| C | 4.816314246 | 15.685750040 | 7.623368407 |
| C | 4.832410511 | 15.280313058 | 8.934579353 |
| C | 4.799217254 | 13.883307929 | 9.228125985 |
| C | 4.755164344 | 12.990998139 | 8.177223149 |
| C | 4.614456584 | 11.154516384 | 3.346687106 |
| C | 4.599739122 | 9.741497345 | 3.225100351 |
| C | 4.559278235 | 9.064928886 | 2.006460457 |
| C | 4.531346863 | 9.785634802 | 0.807715430 |
| C | 4.547221175 | 11.171302997 | 0.865904078 |
| C | 4.589722824 | 11.830148675 | 2.107905868 |
| C | 4.954272147 | 18.199196184 | 1.534084813 |
| C | 6.185218529 | 17.938644441 | 0.927819996 |
| C | 3.956620243 | 18.917360144 | 0.876318048 |
| H | 4.636828298 | 15.619655801 | 1.272773479 |
| H | 4.801654147 | 17.226002551 | 5.271984759 |
| H | 4.839615021 | 16.743091216 | 7.379239892 |
| H | 4.869243221 | 16.010848918 | 9.737325840 |
| H | 4.808145951 | 13.516962647 | 10.248687385 |
| H | 4.730465673 | 11.922200222 | 8.360430452 |
| H | 4.623914336 | 9.143293739 | 4.128102062 |
| H | 4.549775122 | 7.978038505 | 1.993852978 |
| H | 4.499024881 | 9.278025389 | -0.151920211 |
| H | 4.529159403 | 11.774464888 | -0.037010769 |
| H | 6.952343451 | 17.392683171 | 1.467406469 |
| H | 3.015143314 | 19.110140104 | 1.380881697 |
| N | 4.740509695 | 13.361665687 | 6.875447646 |
| O | 4.605760339 | 13.201298310 | 1.926246928 |
| O | 4.721173712 | 17.837850410 | 2.858466816 |
| Pt | 4.670298257 | 12.116243751 | 5.141817332 |
| C | 4.723752197 | 10.322767808 | 6.274965245 |
| C | 5.827891804 | 9.680349485 | 6.842520968 |
| N | 3.683483088 | 9.502762363 | 6.655648849 |
| C | 7.281512496 | 10.031110232 | 6.782395531 |
| N | 5.354671662 | 8.566798572 | 7.478205139 |
| C | 2.285748180 | 9.630058913 | 6.349452710 |
| N | 4.047452258 | 8.441432883 | 7.382730663 |
| C | 8.044933018 | 9.321381145 | 5.644318099 |
| H | 7.350989183 | 11.114622134 | 6.643776810 |
| H | 7.756797858 | 9.799995455 | 7.743279390 |
| C | 6.102215437 | 7.568373238 | 8.241492943 |
| C | 1.651584478 | 10.864372890 | 6.504360513 |
| C | 1.584203037 | 8.502065060 | 5.916666261 |
| C | 9.526734145 | 9.712091970 | 5.603220130 |
| H | 7.569830011 | 9.570972463 | 4.687265642 |
| H | 7.954326019 | 8.233731448 | 5.764223684 |
| H | 6.845303170 | 7.098263759 | 7.595714821 |
| H | 6.597149997 | 8.053281053 | 9.084969146 |
| H | 5.392880641 | 6.824787921 | 8.600898595 |
| C | 0.291720452 | 10.967698876 | 6.206247557 |
| C | 0.223629227 | 8.618375256 | 5.630073600 |
| H | 2.100295235 | 7.555312509 | 5.800071315 |
| C | 10.294667808 | 9.020632093 | 4.473473409 |
| H | 9.608639988 | 10.801608076 | 5.489650385 |
| H | 9.992440816 | 9.467607530 | 6.567668898 |
| C | -0.423052535 | 9.849171527 | 5.770086545 |
| H | -0.208928265 | 11.923612014 | 6.326671683 |
| H | -0.326790891 | 7.746637337 | 5.289503235 |
| H | 10.259677603 | 7.929633021 | 4.578428567 |
| H | 9.871849312 | 9.272955606 | 3.493763027 |
| H | 11.348622461 | 9.319977450 | 4.467513270 |
| H | -1.481169702 | 9.935918124 | 5.541691845 |
| H | 2.210212917 | 11.721380578 | 6.861702638 |

**15**

| | | | |
|---|---|---|---|
| C | 6.211304957 | 1.475706392 | -0.688806657 |
| C | 7.035491431 | 0.576837085 | 0.003999119 |
| C | 8.534143785 | 0.753362899 | 0.032058764 |
| H | 6.662521961 | 2.295848777 | -1.242467614 |
| H | 9.048284462 | -0.206437817 | 0.143226053 |
| H | 8.841548246 | 1.385420942 | 0.875595922 |
| H | 8.897245633 | 1.234851536 | -0.881335913 |
| C | -2.132211423 | -3.559335883 | -0.061667882 |
| C | -1.603722174 | -4.845804892 | -0.087507776 |
| C | -0.217128747 | -4.993274621 | -0.094643032 |
| C | 0.590531626 | -3.858829473 | -0.075670822 |
| C | 0.007268627 | -2.588642877 | -0.050031868 |
| C | 0.749638460 | -1.312034880 | -0.027079844 |
| C | -0.054268434 | -0.168379715 | -0.009704349 |
| C | 0.495831874 | 1.122002927 | 0.008112545 |
| C | 1.886885422 | 1.259144921 | 0.011493524 |
| C | 2.722332201 | 0.119018606 | -0.006866415 |
| C | 2.146739540 | -1.164233293 | -0.028382575 |
| C | -0.544958373 | 2.171877158 | 0.032780841 |
| C | -1.891452344 | 1.683192499 | 0.030551195 |
| C | -2.932435085 | 2.611886914 | 0.053027439 |
| C | -2.697704255 | 4.003002253 | 0.077265398 |
| C | -1.374781818 | 4.453840594 | 0.079790911 |
| C | -0.308420208 | 3.547556646 | 0.057510539 |
| C | -3.857676713 | 4.969722773 | 0.095236565 |
| C | 4.201656763 | 0.276444208 | -0.001969061 |
| C | 4.824528067 | 1.331438751 | -0.692737417 |
| C | 6.415538006 | -0.477554368 | 0.690331322 |
| C | 5.029150669 | -0.625474385 | 0.690784848 |
| C | -3.981342187 | -0.507619471 | 0.005750974 |
| H | -3.203242516 | -3.390319141 | -0.055126625 |
| H | -2.268802195 | -5.701921054 | -0.101198683 |
| H | 0.233043664 | -5.980973204 | -0.114425028 |
| H | 1.670133865 | -3.954893522 | -0.080298917 |
| H | 2.342688198 | 2.244416442 | 0.050544995 |
| H | 2.796515909 | -2.032752671 | -0.067413499 |
| H | -3.965081660 | 2.269358053 | 0.052523520 |
| H | -1.171851789 | 5.521913581 | 0.099181758 |
| H | 0.711560162 | 3.924674765 | 0.060064403 |
| H | 4.220215237 | 2.032911450 | -1.260775644 |
| H | 7.026837743 | -1.189941708 | 1.239565505 |
| H | 4.582960049 | -1.441799788 | 1.251768200 |
| H | -3.513042875 | 6.005601536 | 0.163740070 |
| H | -4.524196197 | 4.777975471 | 0.944703545 |
| H | -4.467012583 | 4.877875573 | -0.812443380 |
| N | -1.357078749 | -2.462239731 | -0.043740725 |
| O | -5.127649436 | -0.582228020 | 0.013623998 |
| Pt | -2.037554060 | -0.362838608 | -0.004230684 |

**15-T$_1$**

<S$^2$> = 2.015629

| | | | |
|---|---|---|---|
| C | -0.819557592 | 3.148117333 | 3.424929381 |
| C | -0.670072544 | 4.033971394 | 2.344679571 |
| C | -1.443567965 | 3.837335907 | 1.065553992 |
| H | -1.480173584 | 2.290270667 | 3.323861204 |
| H | -0.971919263 | 4.359961677 | 0.228186388 |
| H | -2.466290832 | 4.225488493 | 1.160719138 |
| H | -1.526829015 | 2.776281562 | 0.808283375 |
| C | 6.491263746 | 6.725137336 | 8.855566681 |
| C | 7.282389396 | 7.244046694 | 7.844859196 |
| C | 6.814230623 | 7.188347349 | 6.522545848 |
| C | 5.574454743 | 6.615346921 | 6.272068370 |
| C | 4.809373282 | 6.102747948 | 7.327359619 |
| C | 3.493622044 | 5.478164698 | 7.201457186 |
| C | 2.933393203 | 5.037094916 | 8.436063957 |
| C | 1.636786973 | 4.389476829 | 8.474318921 |
| C | 0.936656590 | 4.197998926 | 7.275780236 |
| C | 1.480233159 | 4.629743747 | 6.054816555 |
| C | 2.769926419 | 5.274245817 | 6.039693410 |
| C | 1.269415362 | 4.044806610 | 9.810811838 |
| C | 2.264027508 | 4.403618218 | 10.827234702 |
| C | 1.963899408 | 4.085801284 | 12.163012383 |
| C | 0.774252029 | 3.457160292 | 12.531445005 |
| C | -0.179417886 | 3.118621978 | 11.519953280 |
| C | 0.062125809 | 3.404469367 | 10.197592093 |
| C | 0.472595777 | 3.129606401 | 13.966429292 |
| C | 0.754302110 | 4.426119390 | 4.786555228 |
| C | -0.128593241 | 3.337197624 | 4.616840279 |
| C | 0.209063434 | 5.114776231 | 2.507605451 |
| C | 0.907551423 | 5.308923072 | 3.696881824 |
| C | 4.895254162 | 5.567902364 | 11.774078377 |
| H | 6.815508110 | 6.748124107 | 9.889987920 |
| H | 8.243906415 | 7.682552332 | 8.088298564 |
| H | 7.409421993 | 7.585914194 | 5.706671531 |
| H | 5.189194534 | 6.560116956 | 5.259547268 |
| H | -0.046540279 | 3.738990945 | 7.287747580 |
| H | 3.180953353 | 5.574668531 | 5.081343686 |
| H | 2.675817870 | 4.334345667 | 12.945826986 |
| H | -1.103905239 | 2.628286196 | 11.813038946 |
| H | -0.672018148 | 3.139908149 | 9.441690387 |
| H | -0.249489577 | 2.612879883 | 5.416435696 |
| H | 0.344445840 | 5.819995315 | 1.691194660 |
| H | 1.557799151 | 6.173710618 | 3.785592585 |
| H | -0.445981486 | 3.631698146 | 14.296382154 |
| H | 1.286947676 | 3.431003769 | 14.630172452 |
| H | 0.302064818 | 2.053010719 | 14.094138601 |
| N | 5.282442550 | 6.164392177 | 8.624300824 |
| O | 5.474794421 | 5.723400252 | 12.765621972 |
| Pt | 3.914566649 | 5.304467293 | 10.096164426 |

**17**

| | | | |
|---|---|---|---|
| C | -0.712747551 | 2.906537378 | 3.274036033 |
| C | -0.583944438 | 3.779155308 | 2.183021938 |
| C | -1.340408305 | 3.539600715 | 0.898876139 |
| H | -1.344015844 | 2.025537603 | 3.181558462 |
| H | -0.853590970 | 4.029970846 | 0.050322076 |
| H | -2.362836965 | 3.934919240 | 0.961495043 |
| H | -1.423069967 | 2.470639679 | 0.676679835 |
| C | 6.511635165 | 6.521130616 | 8.767013948 |
| C | 7.366440153 | 6.919479691 | 7.744431038 |
| C | 6.929289579 | 6.783120292 | 6.426229556 |
| C | 5.664471868 | 6.257601616 | 6.180038360 |
| C | 4.844696731 | 5.871848441 | 7.248258310 |
| C | 3.489151184 | 5.303846357 | 7.107935744 |
| C | 2.863875008 | 4.989558305 | 8.321989058 |
| C | 1.570929388 | 4.432886212 | 8.351620709 |
| C | 0.905170909 | 4.197796336 | 7.145915853 |
| C | 1.519733531 | 4.509456779 | 5.908960806 |
| C | 2.813137773 | 5.060006518 | 5.898321909 |
| C | 1.112041715 | 4.187628115 | 9.737791199 |
| C | 2.056397353 | 4.555916578 | 10.759873348 |
| C | 1.679125682 | 4.344167006 | 12.090556006 |
| C | 0.430780110 | 3.796052699 | 12.452113715 |
| C | -0.464095071 | 3.449185427 | 11.435062560 |
| C | -0.125410562 | 3.643399986 | 10.091030665 |
| C | 0.080983559 | 3.589469998 | 13.907569012 |
| C | 0.799533567 | 4.258790522 | 4.631416022 |
| C | -0.037509771 | 3.138795156 | 4.471403807 |
| C | 0.253884658 | 4.892568508 | 2.338211607 |
| C | 0.928588140 | 5.131073800 | 3.535598045 |
| H | 6.804221976 | 6.606712283 | 9.808118591 |
| H | 8.344035217 | 7.323966799 | 7.982570749 |
| H | 7.565398521 | 7.082438026 | 5.598628142 |
| H | 5.307800793 | 6.145593208 | 5.162247775 |
| H | -0.103940853 | 3.793451253 | 7.141324023 |
| H | 3.286403059 | 5.272582380 | 4.944288562 |
| H | 2.369251340 | 4.610104297 | 12.889031557 |
| H | -1.432229067 | 3.024795338 | 11.691451636 |
| H | -0.838491522 | 3.364993379 | 9.317815920 |
| H | -0.142648815 | 2.427426589 | 5.285709696 |
| H | 0.373161940 | 5.590076002 | 1.512191893 |
| H | 1.550174088 | 6.017458387 | 3.626089735 |
| H | -0.928991029 | 3.184662155 | 14.022998763 |
| H | 0.132174947 | 4.530156516 | 14.469526781 |
| H | 0.777713938 | 2.894042074 | 14.392202819 |
| N | 5.289070659 | 6.012827399 | 8.539599073 |
| Pt | 3.795451104 | 5.329171457 | 10.040234888 |
| C | 4.843702328 | 5.648156804 | 11.853557783 |
| C | 5.840812883 | 4.861819007 | 12.444573733 |
| N | 4.706773370 | 6.667069658 | 12.775976383 |
| C | 6.441512036 | 3.570957843 | 11.982047946 |
| N | 6.183580779 | 5.464070221 | 13.622293420 |
| C | 3.810235025 | 7.787070871 | 12.738166086 |
| N | 5.508372041 | 6.572880764 | 13.843924282 |
| C | 5.776757565 | 2.319634570 | 12.593865218 |
| H | 6.340461618 | 3.536312862 | 10.892618996 |
| H | 7.515527005 | 3.560090807 | 12.204358483 |
| C | 7.182414796 | 5.044785237 | 14.603840010 |
| C | 3.642700805 | 8.502841522 | 11.550634871 |
| C | 3.129271485 | 8.149611474 | 13.903469000 |
| C | 6.396885037 | 1.015958314 | 12.077972171 |
| H | 4.704568453 | 2.336260954 | 12.360303685 |

| | | | |
|---|---|---|---|
| H | 5.859884061 | 2.358520858 | 13.687979822 |
| H | 6.888605744 | 4.087999094 | 15.038904779 |
| H | 8.152253392 | 4.945717615 | 14.113242005 |
| H | 7.231398193 | 5.809223700 | 15.377642301 |
| C | 2.768070483 | 9.590667012 | 11.532329201 |
| C | 2.264840869 | 9.244349342 | 13.873114070 |
| H | 3.270890196 | 7.576163680 | 14.813045102 |
| C | 5.742355487 | -0.233199025 | 12.674179137 |
| H | 6.313921263 | 0.986732987 | 10.982995009 |
| H | 7.471775636 | 1.011581055 | 12.305011595 |
| C | 2.079257953 | 9.963428144 | 12.689397852 |
| H | 2.634669984 | 10.151528537 | 10.612178496 |
| H | 1.730065385 | 9.527408317 | 14.774730024 |
| H | 5.838950761 | -0.249870557 | 13.766396799 |
| H | 4.672864248 | -0.273007139 | 12.435696626 |
| H | 6.203566798 | -1.148148141 | 12.286008046 |
| H | 1.401668917 | 10.811843105 | 12.669226091 |
| H | 4.194279386 | 8.216641457 | 10.662683543 |

**17-T$_1$**

$\langle S^2 \rangle$ = 2.011276

| | | | |
|---|---|---|---|
| C | -0.815738297 | 2.975918892 | 3.350233112 |
| C | -0.564459957 | 3.754678505 | 2.209939354 |
| C | -1.260712857 | 3.475746156 | 0.902559519 |
| H | -1.535914502 | 2.163360717 | 3.292874765 |
| H | -0.636372335 | 2.849480640 | 0.251572894 |
| H | -1.467848951 | 4.400835936 | 0.354990305 |
| H | -2.205051825 | 2.945483580 | 1.057291952 |
| C | 6.493775380 | 6.524459194 | 8.806490253 |
| C | 7.353972102 | 6.926489649 | 7.803880557 |
| C | 6.932666682 | 6.796373589 | 6.450189379 |
| C | 5.681069195 | 6.274399078 | 6.192018351 |
| C | 4.827925168 | 5.872902933 | 7.244548936 |
| C | 3.507519916 | 5.324772618 | 7.097660127 |
| C | 2.843661977 | 4.997909711 | 8.347734272 |
| C | 1.524822846 | 4.433595707 | 8.373312795 |
| C | 0.854260813 | 4.189366309 | 7.178704416 |
| C | 1.491192192 | 4.510303214 | 5.942757610 |
| C | 2.801194635 | 5.068483102 | 5.922184945 |
| C | 1.079174067 | 4.200694274 | 9.741563651 |
| C | 2.037545879 | 4.571223290 | 10.761820350 |
| C | 1.658471370 | 4.358048775 | 12.097316721 |
| C | 0.414310072 | 3.817574587 | 12.453100842 |
| C | -0.497678818 | 3.468572745 | 11.432836840 |
| C | -0.168958402 | 3.657001582 | 10.097925422 |
| C | 0.042673233 | 3.606245234 | 13.898867380 |
| C | 0.795996096 | 4.253691164 | 4.668295278 |
| C | -0.157302135 | 3.218863124 | 4.552015642 |
| C | 0.375964002 | 4.792295632 | 2.323168039 |
| C | 1.044322040 | 5.035592936 | 3.518052739 |
| H | 6.780063129 | 6.608718711 | 9.850853040 |
| H | 8.327478902 | 7.331655903 | 8.058505779 |
| H | 7.582194978 | 7.102348320 | 5.635778131 |
| H | 5.334269921 | 6.167063410 | 5.168102666 |
| H | -0.157444108 | 3.799802031 | 7.172449700 |
| H | 3.259299770 | 5.263347750 | 4.957438690 |
| H | 2.348838629 | 4.618752556 | 12.895841168 |
| H | -1.463848572 | 3.048099727 | 11.701034824 |
| H | -0.881171456 | 3.380625450 | 9.324492655 |
| H | -0.363982191 | 2.579842564 | 5.404426812 |
| H | 0.579027524 | 5.424341763 | 1.462022323 |
| H | 1.740821786 | 5.866464115 | 3.569409211 |
| H | -0.854564132 | 4.179399705 | 14.163571630 |
| H | 0.850290592 | 3.910842035 | 14.570203017 |
| H | -0.187254143 | 2.552253035 | 14.097876603 |
| N | 5.262617163 | 6.008162595 | 8.581399789 |
| Pt | 3.766272043 | 5.332007318 | 10.022868085 |
| C | 4.838434882 | 5.656956598 | 11.841184772 |
| C | 5.851080457 | 4.879183700 | 12.414524403 |
| N | 4.691503749 | 6.662431155 | 12.775048894 |
| C | 6.467803520 | 3.602786659 | 11.933787034 |
| N | 6.192842651 | 5.473416920 | 13.596948740 |
| C | 3.780010511 | 7.771497026 | 12.754154436 |
| N | 5.501722082 | 6.568859917 | 13.835468189 |
| C | 5.822698987 | 2.335717160 | 12.534278273 |
| H | 6.361984157 | 3.579937002 | 10.844560441 |
| H | 7.542704649 | 3.604799702 | 12.151508652 |
| C | 7.206375889 | 5.059591246 | 14.565833653 |
| C | 3.622125182 | 8.517554925 | 11.584148354 |
| C | 3.077782660 | 8.092320576 | 13.918681681 |
| C | 6.459089664 | 1.046505023 | 12.002150120 |
| H | 4.749556890 | 2.339962998 | 12.304248151 |
| H | 5.908848779 | 2.363940898 | 13.628504585 |
| H | 6.938080219 | 4.087402278 | 14.982707848 |
| H | 8.176025516 | 4.994540159 | 14.069207632 |
| H | 7.239920170 | 5.809482468 | 15.354550805 |
| C | 2.734406703 | 9.594984886 | 11.582978343 |
| C | 2.199820114 | 9.176821510 | 13.905516916 |
| H | 3.214094639 | 7.495751175 | 14.814163783 |
| C | 5.823667699 | -0.217957443 | 12.586651686 |
| H | 6.373087936 | 1.028200005 | 10.907192484 |
| H | 7.534612788 | 1.054510385 | 12.225917006 |
| C | 2.023314260 | 9.926135489 | 12.739320698 |
| H | 2.608446780 | 10.180510516 | 10.677276400 |
| H | 1.648519928 | 9.428666382 | 14.806421550 |
| H | 5.923916572 | -0.245163753 | 13.678329375 |
| H | 4.754073910 | -0.270003275 | 12.350924283 |
| H | 6.296252458 | -1.122150160 | 12.187144400 |
| H | 1.335795674 | 10.766762125 | 12.732341538 |
| H | 4.191697504 | 8.263654305 | 10.697868168 |

## 5. References


(1) Fanizzi, F. P.; Intini, F. P.; Natile, G. Nucleophilic Attack of Methanol on Bis(Benzonitrile)Dichloroplatinum: Formation of Mono- and Bis-Imido Ester Derivatives. *J. Chem. Soc., Dalton Trans.* **1989**, 947.

(2) Li, Q.; Shi, C.; Zhang, X.; Tao, P.; Zhao, Q.; Yuan, A. Comparison of Structural and Optical Properties for N-Embedded Polycyclic and Non-Embedded Cationic Phosphorescent Iridium(III) Complexes. *Eur. J. Inorg. Chem.* **2019**, 1343–1348.

(3) Poveda, D.; Vivancos, Á.; Bautista, D.; González-Herrero, P. Photochemically Induced Cyclometalations at Simple Platinum(II) Precursors. *Inorg. Chem.* **2023**, *62*, 6207–6213.

(4) Poulain, A.; Canseco-Gonzalez, D.; Hynes-Roche, R.; Müller-Bunz, H.; Schuster, O.; Stoeckli-Evans, H.; Neels, A.; Albrecht, M. Synthesis and Tunability of Abnormal 1,2,3-Triazolylidene Palladium and Rhodium Complexes. *Organometallics* **2011**, *30*, 1021–1029.

(5) Sheldrick, G. M. A Short History of SHELX. *Acta Crystallogr., Sect. A Found. Crystallogr.* **2008**, *64*, 112–122.

(6) Sheldrick, G. M. SHELXT – Integrated Space-Group and Crystal-Structure Determination. *Acta Crystallogr. Sect. A Found. Adv.* **2015**, *71*, 3–8.

(7) Frisch, M. J.; Trucks, G. W.; Schlegel, H. B.; Scuseria, G. E.; Robb, M. A.; Cheeseman, J. R.; Scalmani, G.; Barone, V.; Petersson, G. A.; Nakatsuji, H.; Li, X.; Caricato, M.; Marenich, A. V.; Bloino, J.; Janesko, B. G.; Gomperts, R.; Mennucci, B.; Hratchian, H. P.; Ortiz, J. V.; et al. Gaussian 16 (Revision A.03); Gaussian Inc.: Wallingford CT, 2016.

(8) Becke, A. Density Functional Thermochemistry III The Role of Exact Exchange. *J. Chem. Phys.* **1993**, *98*, 5648–5652.

(9) Lee, C. T.; Yang, W. T.; Parr, R. G. Development of The Colle-Salvetti Correlation-Energy Formula into a Functional of the Electron-Density. *Phys. Rev. B* **1988**, *37*, 785–789.

(10) Hariharan, P. C.; Pople, J. A. Influence of Polarization Functions on Molecular-Orbital Hydrogenation Energies. *Theor. Chim. Acta* **1973**, *28*, 213–222.

(11) Francl, M. M.; Pietro, W. J.; Hehre, W. J.; Binkley, J. S.; Gordon, M. S.; Defrees, D. J.; Pople, J. A. Self-Consistent Molecular-Orbital Methods. 23. A Polarization-Type Basis Set for 2nd-Row Elements. *J. Chem. Phys.* **1982**, *77*, 3654–3665.

(12) Hay, P. J.; Wadt, W. R. Ab Initio Effective Core Potentials for Molecular Calculations–Potentials for K to Au Including the Outermost Core Orbitals. *J. Chem. Phys.* **1985**, *82*, 299–310.

(13) Escudero, D.; Thiel, W. Exploring the Triplet Excited State Potential Energy Surfaces of a Cyclometalated Pt(II) Complex: Is There Non-Kasha Emissive Behavior? *Inorg Chem* **2014**, *53*, 11015–11019.

(14) Marenich, A. V.; Cramer, C. J.; Truhlar, D. G. Universal Solvation Model Based on Solute Electron Density and on a Continuum Model of the Solvent Defined by the Bulk Dielectric Constant and Atomic Surface Tensions. *J. Phys. Chem. B* **2009**, *113*, 6378–6396.
